# Three-component coupling of aryl iodides, allenes, and aldehydes catalyzed by a Co/Cr-hybrid catalyst

**DOI:** 10.3762/bjoc.14.118

**Published:** 2018-06-11

**Authors:** Kimihiro Komeyama, Shunsuke Sakiyama, Kento Iwashita, Itaru Osaka, Ken Takaki

**Affiliations:** 1 Department of Applied Chemistry, Graduate School of Engineering, Hiroshima University, 1-4-1 Higashi-Hiroshima City 739-8527, Japan

**Keywords:** chromium, cobalt, diastereoselective, homoallyl alcohols, hybrid catalyst, three-component coupling

## Abstract

The cobalt/chromium-catalyzed three-component coupling of aryl iodides, allenes, and aldehydes has been developed to afford multi-substituted homoallylic alcohols in a diastereoselective manner. Control experiments for understanding the reaction mechanism reveal that the cobalt catalyst is involved in the oxidative addition and carbometalation steps in the reaction, whereas the chromium salt generates highly nucleophilic allylchromium intermediates from allylcobalt species, without the loss of stereochemical information, to allow the addition to aldehydes.

## Introduction

**Scheme 1:**
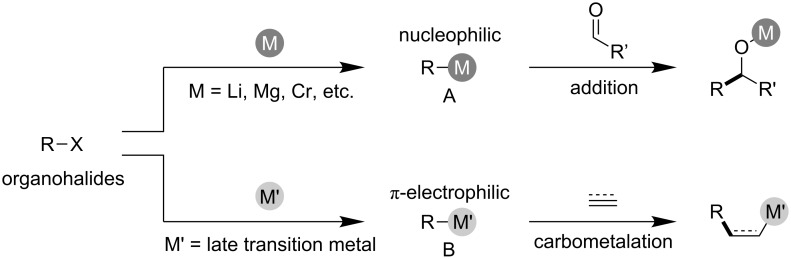
Nucleophilic and π-electrophilic characters of organometallics depending on the central metals.

Carbon–carbon bond formation is the fundamental and central transformation of synthetic organic chemistry. The elaboration and extension of a carbon framework via a series of carbon–carbon bond-forming reactions are extremely important for medicinal chemistry and agrochemical and natural product synthesis. In these bond formations, organometallics play an essential role because they possess various reactivities depending on the central metal ions that they own. For example, carbon has strong nucleophilicity when bonded to metals with low electronegativity, as demonstrated in the reaction of organolithium, organomagnesium, organozinc, and organochromium **A**, to facilitate addition reactions of appropriate carbon electrophiles such as aldehydes (Scheme 1, top) [1]. In contrast, π-electrophilic carbon-connected late transition metals **B** facilitate the carbometalation of carbon–carbon multiple bonds, leading to multi-substituted carbon frameworks (Scheme 1, bottom) [2–3]. The nucleophilic and π-electrophilic organometallic intermediates are used properly in their favorable circumstances.

**Scheme 2:**
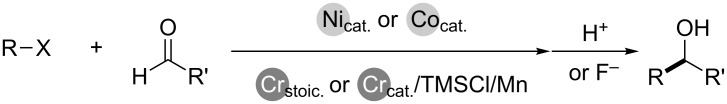
Ni/Cr or Co/Cr-catalyzed NHK reaction.

Transmetalation is one of the most vital elemental processes used to drastically change the reactivity of organometallics, involving a wide range of transition metal-catalyzed reactions. For example, transmetalation between an organonickel (or organocobalt) complex and chromium salt results in the formation of a highly nucleophilic organochromium species, which enables efficient addition to aldehydes to give substituted secondary alcohols, as demonstrated in the Nozaki–Hiyama–Kishi (NHK) reaction (Scheme 2) [4–9]. Although the catalyst combination allows the use of organic halides as carbon nucleophiles, a multicomponent coupling reaction using a similar catalyst combination has had limited success [10–12].

**Scheme 3:**
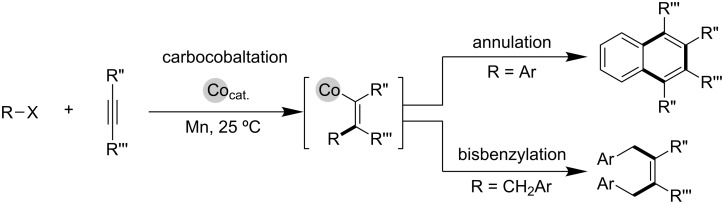
Functionalization of alkynes via carbocobaltation.

**Scheme 4:**
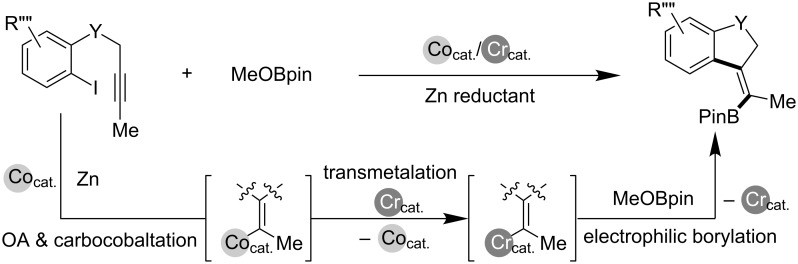
Cyclization/borylation of alkynyl iodoarenes using the Co/Cr catalyst.

**Scheme 5:**
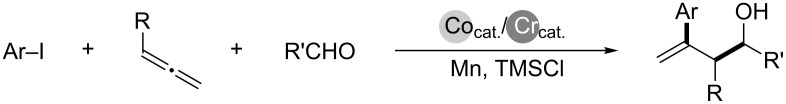
Three-component coupling of aryl iodides, arenes, and aldehydes using Co/Cr catalyst (this work).

We have recently demonstrated the high π-electron affinity of an organocobalt species that enabled a variety of alkyne functionalization reactions to proceed via carbocobaltation (Scheme 3) [13–15]. Furthermore, a combination of the cobalt and chromium catalyst could be applied to alkynyl iodoarene cyclization/borylation to form cyclized vinylboronic esters, in which transmetalation between the generated vinylcobalt and chromium salt was a critical step (Scheme 4) [16]. As part of our continuing work on the cobalt-catalyzed functionalization of carbon–carbon unsaturated bonds, a three-component coupling method is herein reported for the direct synthesis of highly diastereoselective multi-substituted homoallyl alcohols employing a cobalt/chromium hybrid catalyst (Scheme 5).

## Results and Discussion

Initially, suitable reaction conditions were investigated for the three-component coupling reaction between iodobenzene (**1a**), 5-phenylpenta-1,2-diene (**2a**), and 4-methylbenzaldehyde (**3a**) in the presence of CoBr_2_ (10 mol %), CrCl_3_ (20 mol %), and manganese powder (2.0 equiv), using trimethylsilyl chloride (TMSCl, 1.2 equiv) as a trapping reagent [7]. These results are summarized in Table 1. The absence of a ligand afforded the homoallyl alcohol **4a** in 25% yield as a *syn*/*anti* (80:20) mixture of diastereomers, the ratio of which was determined by the coupling constant between the two protons at the C1- and C2-positions of **4a**; a coupling value of ^3^*J* = ca*.* 5.0 Hz indicated the *syn*-form, and a coupling value of ^3^*J* = ca*.* 8.0 Hz indicated the *anti*-form (Table 1, entry 1) [17]. The addition of PPh_3_ (20 mol %) resulted in an increase in the product yield to 45% with a similarly diastereomer ratio (Table 1, entry 2). The use of the chromium catalyst, Mn reductant, and TMSCl was crucial for the coupling (Table 1, entries 3–5). Thus, the removal of CrCl_3_ resulted in the formation of a trace amount of **4a** with the complete consumption of allene **2a**, whereas most of iodoarene **1a** and aldehyde **3a** remained unreacted after the reaction was completed (Table 1, entry 3). Using a Zn reductant instead of Mn resulted in a negligible amount of coupling product (Table 1, entry 4), wherein **1a** was completely consumed [18]. Reaction conditions without the use of TMSCl produced **4a** in a catalytic amount (Table 1, entry 5). Other ligands were also tested (Table 1, entries 5–12), and after the screening of several phosphines and pyridine-type ligands, the latter ligands were found to be the most effective for use in the coupling reaction. Consequently, we found that iminopyridine **L3** was the best choice of ligand, and when used, it resulted in the three-component product **4a** being obtained in 69% yield with a diastereoselectivity ratio of 92:8 (Table 1, entry 13). Additionally, preformed CoBr_2_(**L3**) gave a similar result (Table 1, entry 14). During the transformation, the chromium catalyst ligands inhibited the reaction (Table 1, entries 15–17). Also, the reaction was highly dependent on the solvent used; for example, dimethylformamide (DMF), tetrahydrofuran (THF), 1,4-dioxane, and toluene did not result in any formation of **4a**.

**Table 1:** Screening of the reaction conditions.^a^

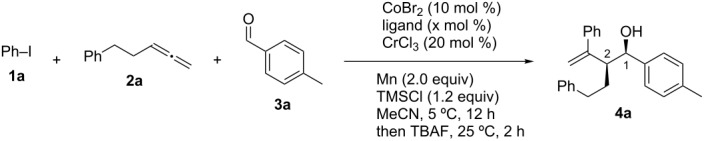				

entry	ligand (x mol %)	Cr catalyst	NMR yield (%)	isomer ratio^b^

1	none	CrCl_3_	25	80:20
2	PPh_3_ (20)	CrCl_3_	45	85:15
3	PPh_3_ (20)	none	5	82:18
4^c^	PPh_3_ (20)	CrCl_3_	trace	–
5^d^	PPh_3_ (20)	CrCl_3_	18	86:14
6	dppe (10)	CrCl_3_	8	91:9
7	dppb (10)	CrCl_3_	trace	–
8	dppf (10)	CrCl_3_	trace	–
9	xantphos (10)	CrCl_3_	trace	–
10	2,2′-bpy (10)	CrCl_3_	45	92:8
11	**L1** (10)	CrCl_3_	52	93:7
12	**L2** (10)	CrCl_3_	64	91:9
13	**L3** (10)	CrCl_3_	69	92:8
14^e^	**–**	CrCl_3_	65	91:9
15	**L3** (10)	CrCl_3_(bpy)	48	94:6
16	**L3** (10)	CrCl_3_(**L3**)	24	92:8
17	**L3** (10)	Cr(salen)Cl	21	91:9

^a^Reaction conditions: **1a** (0.25 mmol), **2a** (0.38 mmol), and **3a** (0.25 mmol). ^b^The ratio (*syn*:*anti*) was determined from the ^1^H NMR of the crude product. ^c^Zn was used instead of Mn. ^d^Without the presence of TMSCl. ^e^CoBr_2_(**L3**) was used instead of CoBr_2_. 
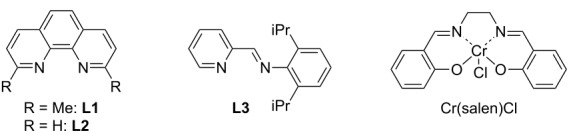

**Scheme 6:**
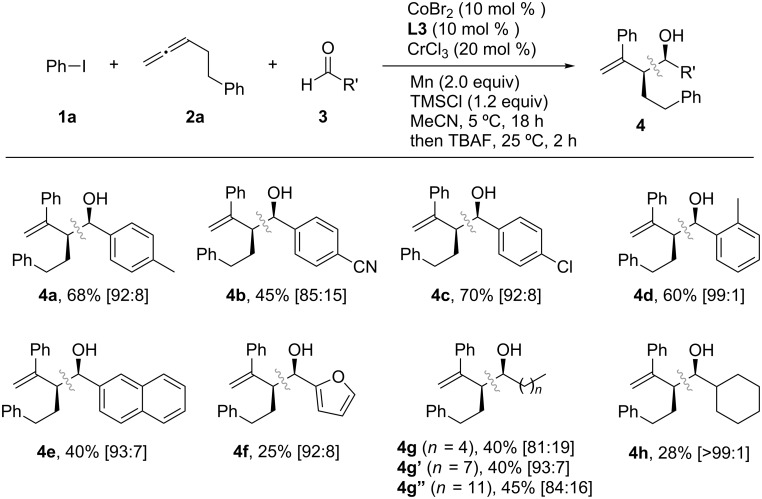
Screening of aldehydes in the Co/Cr-catalyzed three-component coupling reaction. All yields are determined after isolation. The values in brackets indicate the diastereomer ratio of the *syn* and *anti* products, determined from ^1^H NMR spectra.

**Scheme 7:**
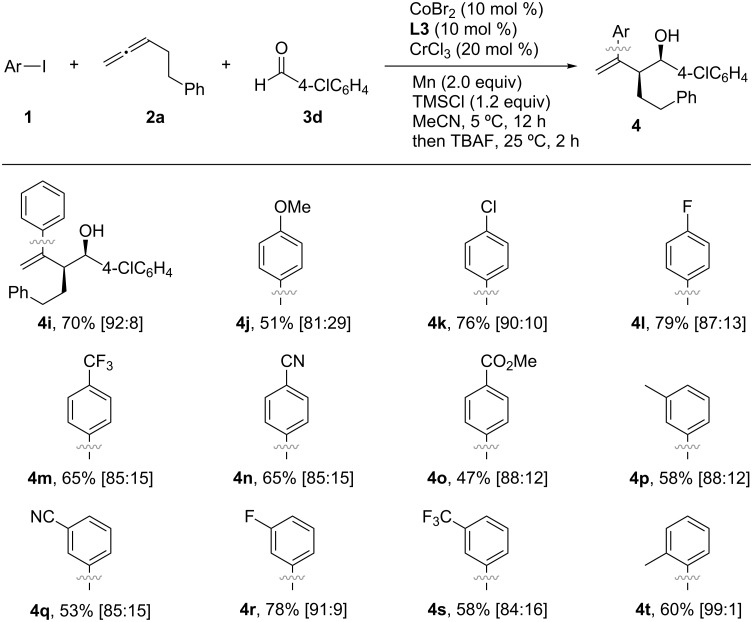
Screening of aryl iodides in the Co/Cr-catalyzed three-component coupling reaction. All yields are determined after isolation. The values in brackets indicate the diastereomer ratio of the *syn* and *anti* products, determined from ^1^H NMR spectra.

**Scheme 8:**
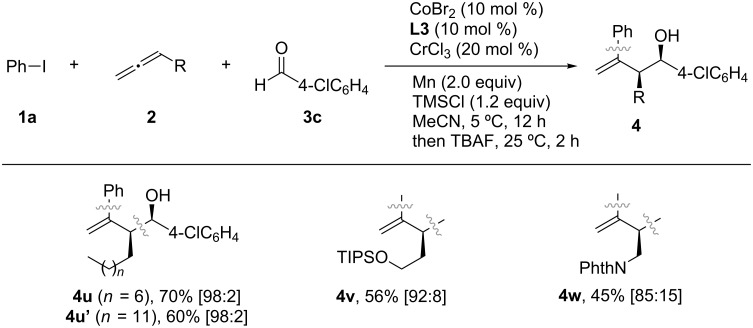
Screening of allenes in the Co/Cr-catalyzed three-component coupling reaction. All yields are determined after isolation. The values in brackets indicate the diastereomer ratio of the *syn* and *anti* products, determined from ^1^H NMR.

With the optimized conditions in hand, the use of aldehydes in the Co/Cr-catalyzed three-component coupling reaction was explored, as shown in Scheme 6. Electron-rich and electron-deficient aryl aldehydes, as well as 2-furylaldehyde, were well tolerated in the reaction, leading to the formation of the corresponding homoallylic alcohols with similar diastereomer ratios (**4a**–**f**). Additionally, alkyl aldehydes were also successfully used in the coupling reaction, albeit resulting in slightly lower yields (**4g**, **4g’**, **4g”** and **4h**). Next, the generality of the reaction was investigated using aryl iodides (Scheme 7) and allenes (Scheme 8). Although aryl bromides and chlorides did not participate in the coupling, a diverse set of functional groups such as methoxy (**4j**), halogens (**4k** and **4l**), trifluoromethyl (**4m**), cyano (**4n**), and ester (**4o**) substituents at the *para*-position of the phenyl ring were successfully used in the reaction, giving rise to the desired coupling products in good yields (47–79%). The homoallylic alcohols **4p**–**t** were formed with a high degree of *syn*-selectivity because of *meta*- and *ortho*-substituted aryl iodides also being well tolerated in the reaction. The protocol was not only limited to the coupling of simple allenes; it was also used with heteroatom-containing functionalized allenes to afford the *syn*-homoallylic alcohols **4u**, **4u’** and **4w** in reasonably good yields (Scheme 8).

**Scheme 9:**
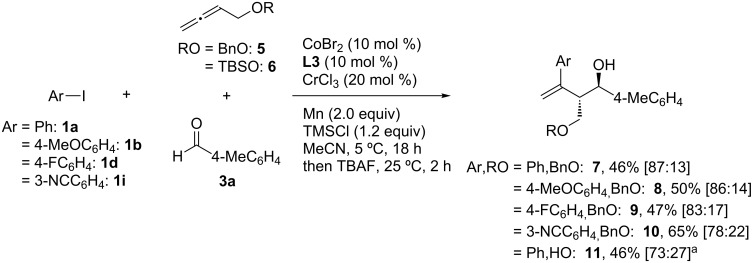
Reversed diastereoselectivity using allenyl ethers **5** and **6**. ^a^4-chlorobenzaldehyde was used instead of **3a**.

During the investigation of allenes, it was observed that, when oxygen substituents were present at the allenyl position, coupling products with reversed diastereoselectivity were obtained (Scheme 9). Thus, treatment of 4-benzyloxybuta-1,2-diene (**5**) with **1** and **3a** under identical reaction conditions mainly afforded the *anti*-configured homoallylic alcohols **7**–**10** in 46–65% yield. A similar reversed diastereoselectivity was observed in the coupling reaction of silyloxy allene **6**, leading to **11** in 46% yield (*anti*/*syn* = 73:27).

**Scheme 10:**
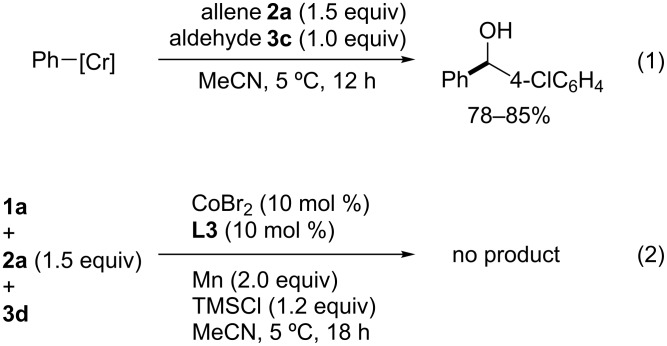
Stoichiometric reaction of phenylchromium(II or III) reagents (reaction 1) and the three-component coupling without CrCl_3_ catalyst (reaction 2).

**Scheme 11:**
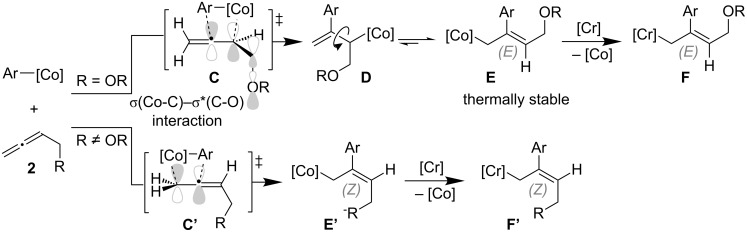
The origin of the diastereoselectivity in the present three-component coupling.

A stoichiometric reaction using phenylchromium(II or III) reagents, generated from the reaction of CrCl_2_ or CrCl_3_(thf)_2_ with phenyllithium [19–20], in the presence of allene **2a** and aldehyde **3c** provided diarylmethanes in 78–85% yields without the visible consumption of allene **2a** (Scheme 10, reaction 1). Furthermore, in the absence of the CrCl_3_ catalyst, allene **2a** was utterly consumed, whereas most of iodide **1a** and aldehyde **3d** were recovered unreacted (Scheme 10, reaction 2). These results indicate that the arylcobalt, rather than the arylchromium intermediate, promoted the allene carbometalation. Additionally, it is thought that the vinylcobalt generated was converted to vinylchromium, which would be inactive in the oligomerization, but is a highly nucleophilic species. According to these findings, the reversed diastereoselectivity in Scheme 9 might be due to stereoelectronic effects [21–22]. Thus, the σ*(C–O) bond stabilizes the forming σ(Co–C) bond in the transition state **C** of the carbocobaltation step (Scheme 11), facilitating the further selective formation of the branched allylcobalt species **D** which could be converted into the thermodynamically more stable (*E*)-allylcobalt species **E** because of the flexible C(vinyl)–C(allyl) single bond. The generated compound **E** then undergoes transmetalation with the chromium salt to give the (*E*)-allylchromium species **F**. In contrast, the carbometalation of a simple allene produces (*Z*)-allylcobalt species **E′** and the corresponding (*Z*)-allylchromium product **F′** would be provided through a transition state such as **C′**, in which the cobalt center is connected to a less sterically hindered terminal allene carbon [23].

**Scheme 12:**
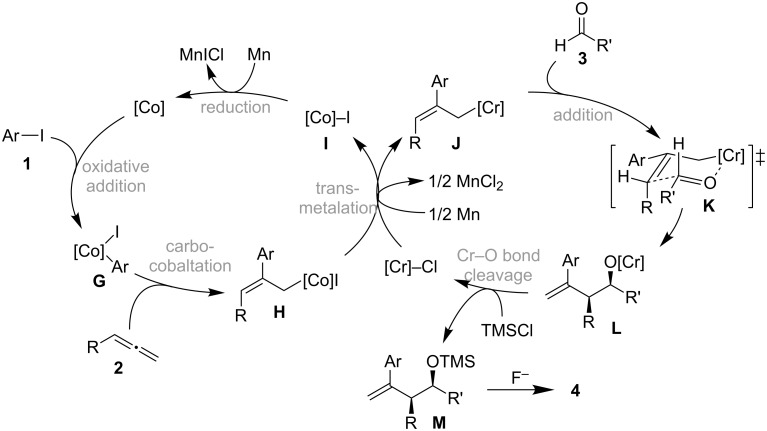
Plausible reaction mechanism of the three-component coupling.

On the basis of these results, a plausible catalytic cycle for the cobalt/chromium-catalyzed three-component coupling reaction is shown in Scheme 12. The three-component coupling starts with the oxidative addition of an aryl iodide **1** to a low-valent cobalt species to form an arylcobalt species **G** that reacts with an allene **2** to stereoselectively generate an allylcobalt **H** via carbocobaltation. Rapid transmetalation between **H** and the chromium salt [8–9] triggers the transfer of the cobalt allyl group to the chromium to afford **I** and the highly nucleophilic allylchromium species **J**, which retains the same stereochemical information on the olefinic moiety as that of **H**. The allylchromium species **J** reacts with the aldehyde **3** at the γ-position of the allyl metal unit via a cyclic six-membered transition state **K** to give the chromium alkoxide **L** [24]. Finally, the Cr–O bond is cleaved by TMSCl, generating the active chromium salt for the transmetalation and the silyl ether **M**, the desilylation of which with a fluoride anion results in the formation of a homoallylic alcohol **4**.

## Conclusion

The cobalt/chromium-catalyzed three-component coupling reaction of aryl iodides, allenes, and aldehydes to produce highly substituted homoallylic alcohols in a diastereoselective manner has been demonstrated. In the coupling reaction, two catalysts played individual roles; the cobalt catalyst activated aryl iodides to form arylcobalt species, which then performed allene carbocobaltation to form stereo-defined substituted (*Z*)-allylcobalt intermediates. The chromium catalyst transformed the generated allylcobalt intermediates into highly nucleophilic allylchromium species, without the isomerization of the olefinic moiety, via transmetalation between the generated allylcobalt intermediate and the chromium salt. Moreover, it was found that an oxygen atom present at the allenyl position resulted in a reversed diastereoselectivity of the homoallylic alcohol products; thus, the allene carbocobaltation regioselectivity could be controlled by the stereoelectronic interaction between the forming σ(C–Co) bond and a neighboring σ*(C–O) bond. Further mechanistic studies and expansion of the substrate scope, including synthetic applications of this three-component coupling, are currently in progress.

## Supporting Information

File 1 Experimental part.

## References

[1] Takai K (2015). Bull Chem Soc Jpn.

[2] Flynn A B, Ogilvie W W (2007). Chem Rev.

[3] Tamaru Y (2005). Modern Organonickel Chemistry.

[4] Okude Y, Hirano S, Hiyama T, Nozaki H (1977). J Am Chem Soc.

[5] Jin H, Uenishi J, Christ W J, Kishi Y (1986). J Am Chem Soc.

[6] Takai K, Tagashira M, Kuroda T, Oshima K, Utimoto K, Nozaki H (1986). J Am Chem Soc.

[7] Fürstner A, Shi N (1996). J Am Chem Soc.

[8] Takai K, Nitta K, Fujimura O, Utimoto K (1989). J Org Chem.

[9] Usanov D L, Yamamoto H (2010). Angew Chem, Int Ed.

[10] Takai K, Matsukawa N, Takahashi A, Fujii T (1998). Angew Chem, Int Ed.

[11] Takai K, Toratsu C (1998). J Org Chem.

[12] Xiong Y, Zhang G (2018). J Am Chem Soc.

[13] Komeyama K, Kashihara T, Takaki K (2013). Tetrahedron Lett.

[14] Komeyama K, Okamoto Y, Takaki K (2014). Angew Chem, Int Ed.

[15] Komeyama K, Asakura R, Fukuoka H, Takaki K (2015). Tetrahedron Lett.

[16] Komeyama K, Kiguchi S, Takaki K (2016). Chem Commun.

[17] Hopkins C D, Malinakova H C (2004). Org Lett.

[18] Fillon H, Gosmini C, Périchon J (2003). J Am Chem Soc.

[19] Daly J J, Sneeden R P A, Zeiss H H (1966). J Am Chem Soc.

[20] Kanno K-i, Liu Y, Iesato A, Nakajima K, Takahashi T (2005). Org Lett.

[21] Ito H, Ito S, Sasaki Y, Matsuura K, Sawamura M (2007). J Am Chem Soc.

[22] Ohmiya H, Makida Y, Li D, Tanabe M, Sawamura M (2010). J Am Chem Soc.

[23] Yoshida Y, Murakami K, Yorimitsu H, Oshima K (2010). J Am Chem Soc.

[24] Hiyama T, Kimura K, Nozaki H (1981). Tetrahedron Lett.

