# Supplementary material for: Three-component coupling of aryl iodides, allenes, and aldehydes catalyzed by a Co/Cr-hybrid catalyst
Source: Beilstein J Org Chem. 2018 Jun 11;14:1413–20. doi: 10.3762/bjoc.14.118 (PMC6009127; doi:10.3762/bjoc.14.118)

Supporting Information

for

Three-component coupling of aryl iodides, allenes, and aldehydes catalyzed by a Co/Cr-hybrid catalyst

Kimihiro Komeyama*[1], Shunsuke Sakiyama[1], Kento Iwashita[1], Itaru Osaka[1] and Ken Takaki[1]

Address: [1]Department of Applied Chemistry, Graduate School of Engineering, Hiroshima University, 1-4-1 Higashi-Hiroshima City 739-8527, Japan

Email: Kimihiro Komeyama - kkome@hiroshima-u.ac.jp

* Corresponding author

Experimental part

1. General Information

All reactions were performed on oven- and flame-dried glassware under argon using standard Schlenk techniques. Flash column chromatography was performed with silica gel 60 (KANTO Chemical Co. Inc., 40–50 nm). Preparative recycling gel permeation chromatography (GPC) was performed with GL Science PU 614 equipped with Shodex GPC H-2001L and H-2002L column (chloroform as an eluent). TLC monitoring was carried

out with silica gel aluminum sheets (Merck, type 60 F_{254}). Gas chromatography (GC) monitoring was carried out on Shimadzu GC-2014. Nuclear magnetic resonance (NMR) spectra were recorded with Varian-400 (^{1}H NMR: 400 MHz; ^{13}C NMR: 101 MHz) spectrometer or Varian-500 (^{1}H NMR: 500 MHz; ^{13}C NMR: 126 MHz) spectrometers, calibrated from residual deuterated chloroform as an internal standard at 7.26 ppm for ^{1}H NMR spectra and at 77.0 ppm for ^{13}C NMR spectra, respectively. Low-resolution mass spectrum (LRMS) was recorded on Shimadzu GCMS-QP2010SE (EI, 70 eV). High-resolution mass spectrum (HRMS) was performed by the Natural Science Center for Basic Research and Development (N-BARD) of Hiroshima University using LTQ Orbitrap XL from Thermo Fisher Scientific.

2. Materials

Ligand **L3** was prepared according to the literature [1]. $CrCl_3$(bpy), $CrCl_3$(**L3**), CrCl(salen) and $CoBr_2$(**L3**) were synthesized based on the reported methods [2,3]. Acetonitrile and *N*,*N*-dimethylformamide (DMF) were dried over activated MS 4 Å, distilled and stored with activated MS 4 Å under argon. Tetrahydrofuran (THF), 1,4-dioxane, and toluene were dried over Na/benzophenone ketyl and distilled prior to use. All allenes were prepared from the reaction of the corresponding terminal alkynes by means of Cu-catalyzed homologation [4]. Unless otherwise noted, commercially available reagents were used as received without further purification.

3. Representative procedure of the Co/Cr-catalyzed three-component coupling (Table 1, entry 13)

In an oven-dried Schlenk tube, Mn powder (27.5 mg, 0.5 mmol) was added and heated at 400 ºC for 15 min under vacuum. After cooling, the Schlenk tube was charged with $CrCl_3$ (4.0 mg, 0.025 mmol) and $CoBr_2$ (5.5 mg, 0.025 mmol) and heated again under vacuum at ca. 80 ºC. After cooling, L3 ligand (6.7 mg, 0.025 mmol), dry MeCN (1 mL), and TMSCl (38 μL, 0.3 mmol) were successively added and then followed by stirring for 10 min until the color of the solution turned to black. After cooling to 5 ºC, the iodide (**1a**, 51 mg, 0.25 mmol), allene (**2a**, 54 mg, 0.375 mmol), and aldehyde (**3a**, 30.4 mg, 0.25 mmol) were added into the solution. The reaction mixture was stirred for 12 h at 5 ºC. The obtained mixture was filtrated, diluted with EtOAc. The EtOAc solution was added 2.0 mL of TBAF solution (1.0 mol/L) and stirred for 2 h at 25 ºC. The aqueous phase was extracted with ethyl acetate. The

combined organic phase was dried over $MgSO_4$. After filtration and removal of the solvent, the residue was purified by silica-gel column chromatography to get **4a** in 61.5 mg (69%) as a diastereo-mixture (*syn/anti* = 92:8).

4. Stoichiometric reaction of Ph-[Cr] species in the presence of allene 2a and aldehyde 3c (Scheme 10, reaction 1)

In an oven-dried Schlenk tube filled with argon, THF (1.0 mL) and bromobenzene (39.3 mg, 0.25 mmol) were added. After cooling to −78 ºC, *n*-butyllithium (156 µL, 0.25 mmol) was slowly added to the tube. The reaction mixture was stirred for 2 hours with keeping −78 ºC. 0.25 mmol of $CrCl_2$, or $CrCl_3(thf)_2$, was placed in another Schlenk tube, poured THF (5 mL), cooled to −78 ºC. The above-prepared phenyl lithium solution was added to the chromium solution via cannula, followed by stirring for overnight with keeping −78 ºC to get Ph-CrCl, or Ph-$CrCl_2$, THF-solution. The Ph-chromium solution was added to a solution of allene **2a** (54.2 mg, 0.38 mmol) and aldehyde **3c** (35.6, 0.25 mmol) in MeCN (1 mL) at −78 ºC. The obtained mixture was warmed to 5 ºC, stirred for 12 hours at 5 ºC. The mixture was quenched with water. The aqueous phase was extracted Et_2O. The ethereal solution was washed with brine, dried over $MgSO_4$. Filtration and evaporation afforded the crude product. ^{1}H NMR using dimethyl terephthalate as an internal standard estimated the yield and the conversion.

4. Spectral data for products

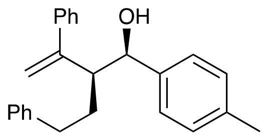

4a

Isolated as a mixture of diastereomers; ^{1}H NMR (500 MHz, $CDCl_3$) ***Syn***: δ 7.34 – 7.21 (m, 7H), 7.19 (d, *J* = 7.3 Hz, 1H), 7.14 (d, *J* = 8.0 Hz, 2H), 7.09 (dd, *J* = 7.6, 3.2 Hz, 4H), 5.50 (d, *J* = 0.9 Hz, 1H), 5.25 (s, 1H), 4.66 (d, *J* = **5.0** Hz, 1H), 3.02 (ddd, *J* = 10.8, 5.0, 3.5 Hz, 1H), 2.79 (ddd, *J* = 14.4, 10.2, 4.6 Hz, 1H), 2.51 (ddd, *J* = 13.9, 10.0, 7.2 Hz, 1H), 2.33 (s, 3H), 2.08 (dddd, *J* = 13.7, 10.4, 7.1, 3.4 Hz, 1H), 2.04 – 1.92 (m, 2H), ***Anti*** (assignable peaks only): δ 5.56 (d, *J* = 1.0 Hz, 1H), 5.33 (s, 1H), 2.36 (s, 3H); ^{13}C NMR (126 MHz, $CDCl_3$) ***Syn***: δ 149.63, 143.06, 142.32, 139.49, 136.67, 128.70, 128.40, 128.28, 128.25, 127.40, 126.66, 126.18, 125.70, 114.50, 75.29, 51.21, 33.42, 29.37, 21.10, ***Anti*** (assignable peaks only): δ

143.20, 129.71, 129.01, 127.08, 127.03, 114.50, 52.84, 33.42, 33.30, 29.72, 29.37; HRMS calcd for $C_{25}H_{24}O$ $[M+H]^+$: 343.2062, found 343.2060.

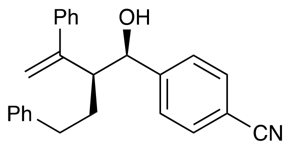

4b

Isolated as a mixture of diastereomers; ^{1}H NMR (500 MHz, $CDCl_3$) ***Syn***: δ 7.52 (d, *J* = 8.3 Hz, 2H), 7.36 – 7.25 (m, 5H), 7.27 – 7.19 (m, 2H), 7.21 – 7.15 (m, 2H), 7.01 (d, *J* = 7.1 Hz, 3H), 5.55 (s, 1H), 5.26 (s, 1H), 4.63 (dd, *J* = **4.6, 1.9** Hz, 1H), 2.94 (dt, *J* = 11.0, 3.7 Hz, 1H), 2.76 (ddd, *J* = 13.9, 9.5, 4.4 Hz, 1H), 2.47 (dt, *J* = 13.9, 8.5 Hz, 1H), 2.10 (brs, 1H), 1.97 (dddd, *J* = 13.8, 11.1, 9.2, 4.5 Hz, 1H), 1.84 (dddd, *J* = 14.0, 9.4, 7.9, 3.3 Hz, 1H), ***Anti***: δ 7.55 (d, *J* = 8.3 Hz, 2H), 7.37 (d, *J* = 8.2 Hz, 2H), 6.97 – 6.92 (m, 2H), 4.71 (dd, *J* = **7.7, 2.7** Hz, 1H), 2.67 (ddd, *J* = 14.3, 9.8, 5.2 Hz, 1H), 2.43 – 2.36 (m, 1H), 1.77 – 1.61 (m, 2H); ^{13}C NMR (126 MHz, $CDCl_3$) ***Syn***: δ 148.78, 147.80, 142.37, 141.71, 131.80, 128.49, 128.35, 128.32, 127.79, 126.92, 126.51, 125.92, 118.90, 110.77, 74.53, 50.74, 33.13, 28.51, ***Anti***: δ 148.93, 147.53, 142.30, 131.92, 128.42, 128.34, 128.24, 127.71, 127.67, 126.82, 118.78, 116.29, 111.39, 52.32, 33.20, 29.69; HRMS calcd for $C_{25}H_{24}NO$ $[M+H]^+$: 354.1858, found 354.1855.

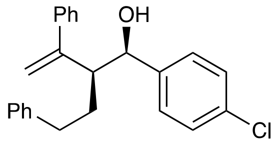

4c

Isolated as a mixture of diastereomers; ^{1}H NMR (500 MHz, $CDCl_3$) ***Syn***: δ 7.36 – 7.17 (m, 9H), 5.43 (s, 1H), 5.14 (s, 1H), 4.62 (d, ***J* = 4.9 Hz**, 1H), 2.94 (dt, *J* = 9.7, 5.0 Hz, 1H), 2.08 (s, 1H), 1.63 (dq, *J* = 16.1, 9.1, 7.4 Hz, 2H), 1.44 (p, *J* = 10.9, 9.2 Hz, 1H), 1.37 – 1.06 (m, 11H), 0.89 (t, *J* = 7.0 Hz, 3H), ***Anti*** (assignable peaks only): δ 7.42 – 7.39 (m, 2H), 7.38 – 7.35 (m, 2H), 5.49 (d, *J* = 1.0 Hz, 1H), 5.25 (d, *J* = 1.0 Hz, 1H), 4.60 (d, *J* = 8.4 Hz, 1H); ^{13}C NMR (101 MHz, $CDCl_3$) ***Syn***: δ 149.61, 143.11, 141.18, 132.67, 128.42 – 128.17 (m), 128.05, 127.70, 127.41, 126.53, 114.34, 75.02, 51.95, 31.84, 29.78, 29.40, 29.23, 27.63, 27.35, 22.63, 14.08, ***Anti*** (assignable peaks only): δ150.17, 142.83, 141.09, 133.31, 128.38, 126.91, 115.48, 53.45, 31.77, 31.13, 29.49, 29.29, 29.12, 27.02, 22.60, 14.17; HRMS calcd for $C_{25}H_{24}ClO$ $[M+H]^+$: 363.1516, found 363.1514.

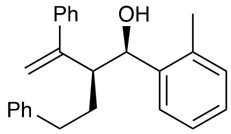

4d

Isolated as a mixture of diastereomers; ^{1}H NMR (500 MHz, $CDCl_3$) ***Syn***: δ 7.53 – 7.45 (m, 1H), 7.35 – 7.24 (m, 5H), 7.25 – 7.18 (m, 3H), 7.19 – 7.12 (m, 2H), 7.12 – 7.04 (m, 3H), 5.49 (s, 1H), 5.35 (s, 1H), 4.80 (d, *J* = **4.5** Hz, 1H), 2.99 (dt, *J* = 10.2, 4.0 Hz, 2H), 2.82 (ddd, *J* = 14.2, 9.7, 4.8 Hz, 1H), 2.53 (dt, *J* = 14.2, 9.8, 7.7 Hz, 1H), 2.18 – 2.04 (m, 2H), 2.02 (s, 3H); ^{13}C NMR (126 MHz, $CDCl_3$) ***Syn***: δ 150.16, 142.92, 142.17, 140.25, 134.25, 130.27, 128.31, 128.23, 128.16, 127.41, 126.93, 126.79, 126.50, 125.68, 125.61, 114.35, 71.51, 48.87, 33.34, 28.33, 19.02; HRMS calcd for $C_{25}H_{27}O$ $[M+H]^+$: 343.2062, found 343.2063.

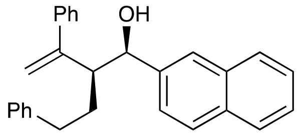

4e

Isolated as a mixture of diastereomers; ^{1}H NMR (500 MHz, $CDCl_3$) ***Syn***: ^{1}H NMR (500 MHz, $CDCl_3$) δ 7.84 – 7.75 (m, 2H), 7.75 – 7.68 (m, 2H), 7.50 – 7.40 (m, 2H), 7.33 – 7.22 (m, 6H), 7.20 (t, *J* = 7.2 Hz, 2H), 7.15 (t, *J* = 7.2 Hz, 1H), 7.05 – 6.98 (m, 2H), 5.53 (d, *J* = 0.7 Hz, 1H), 5.29 (s, 1H), 4.82 (d, ***J* = 4.4 Hz**, 1H), 3.18 – 3.08 (m, 1H), 2.77 (dt, *J* = 14.1, 7.3 Hz, 1H), 2.48 (dt, *J* = 13.8, 8.5 Hz, 1H), 2.12 (s, 1H), 2.01 (q, *J* = 8.5, 6.8 Hz, 2H), ***Anti*** (assignable peaks only): δ 5.57 (s, 1H), 5.36 (s, 1H), 3.09 – 3.03 (m, 1H), 2.64 (ddd, *J* = 14.9, 10.4, 5.9 Hz, 1H), ^{13}C NMR (126 MHz, $CDCl_3$) δ 149.52, 142.90, 142.15, 139.83, 133.15, 132.72, 128.43 (d, *J* = 4.4 Hz), 128.30, 128.01, 127.76, 127.60 (d, *J* = 5.8 Hz), 126.74, 125.99, 125.72 (d, *J* = 13.0 Hz), 125.06, 124.32, 114.72, 75.09, 50.92, 33.39, 28.72; HRMS calcd for $C_{28}H_{27}O$ $[M+H]^+$: 379.2062, found 379.2060.

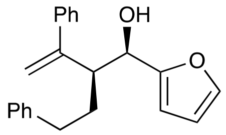

4f

Isolated as a mixture of diastereomers; ^{1}H NMR (500 MHz, $CDCl_3$) ***Syn***: δ 7.20 – 7.07 (m, 8H), 7.04 (t, *J* = 7.3 Hz, 1H), 6.99 (d, *J* = 6.7 Hz, 2H), 6.12 (dd, *J* = 3.2, 1.8 Hz, 1H), 6.01 (d, *J* = 3.1 Hz, 1H), 5.32 (s, 1H), 5.06 (s, 1H), 4.54 (d, *J* = 6.2 Hz, 1H), 3.04 (ddd, *J* = 10.2, 6.1, 3.5 Hz, 1H), 2.65 (ddd, *J* = 13.8, 10.4, 4.6 Hz, 1H), 2.42 (ddd, *J* = 13.8, 10.3, 7.0 Hz, 1H), 2.04 (dddd, *J* = 13.8, 10.3, 7.0, 3.5 Hz, 1H), 1.94 (s, 1H), 1.85 (dtd, *J* = 13.8, 10.4, 4.7 Hz,

1H); ***Anti*** (assignable peaks only): δ 7.29 – 7.23 (m, 1H), 6.91 – 6.86 (m, 1H), 6.15 (dd, *J* = 3.3, 1.8 Hz, 1H), 6.08 (d, *J* = 3.3 Hz, 1H), 4.58 (d, ***J* = 8.6 Hz**, 1H), 2.54 (ddd, *J* = 14.8, 9.8, 5.9 Hz, 1H), 2.31 (ddd, *J* = 13.8, 9.7, 7.2 Hz, 1H), 2.15 (s, 1H), 1.62 – 1.53 (m, 1H); ^{13}C NMR (126 MHz, $CDCl_3$) ***Syn***: δ 155.02, 148.88, 142.50, 142.11, 141.32, 128.28, 128.14, 128.11, 127.26, 126.50, 125.61, 114.54, 110.00, 106.57, 70.56, 48.54, 33.19, 30.84; ***Anti*** (assignable peaks only): δ 154.57, 149.55, 142.36, 141.95, 141.88, 128.28, 127.43, 126.99, 115.66, 107.62, 70.43, 50.15, 33.20, 32.96; HRMS calcd for $C_{22}H_{23}O_2$ $[M+H]^+$: 319.1698, found 319.1700.

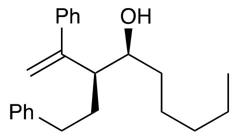

4g

Isolated as a mixture of diastereomers; ^{1}H NMR (500 MHz, $CDCl_3$) ***Syn***: δ 7.41 – 7.11 (m, 10H), 5.52 (s, 1H), 5.20 (s, 1H), 3.51 (dt, *J* = 8.9, **4.6** Hz, 1H), 2.85 (ddd, *J* = 14.1, 9.9, 4.6 Hz, 1H), 2.67 (ddd, *J* = 11.2, 5.0, 3.4 Hz, 1H), 2.58 (ddd, *J* = 13.7, 9.7, 7.3 Hz, 1H), 2.13 (dddd, *J* = 13.5, 10.4, 7.3, 3.4 Hz, 1H), 2.02 – 1.82 (m, 2H), 1.76 – 1.12 (m, 9H), 0.85 (t, *J* = 7.0 Hz, 3H); ***Anti*** (assignable peaks only): δ 5.26 (d, *J* = 1.0 Hz, 1H), 3.71 – 3.61 (m, 1H), 2.80 – 2.70 (m, 1H), 0.86 (t, *J* = 7.1 Hz, 3H); ^{13}C NMR (126 MHz, $CDCl_3$) ***Syn***:δ 149.98, 142.93, 142.34, 128.33, 128.26, 128.24, 128.20, 127.39, 127.32, 126.64, 126.55, 125.66, 114.93, 113.80, 73.49, 49.15, 34.51, 33.38, 31.65, 29.86, 25.70, 22.47, 13.90; ***Anti*** (assignable peaks only): δ 149.83, 143.44, 142.21, 127.32, 126.64, 114.93, 73.77, 50.27, 33.54, 33.09, 31.71, 25.47; HRMS calcd for $C_{23}H_{31}O$ $[M+H]^+$: 323.2375, found 323.2372.

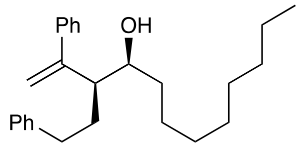

4g'

Isolated as a mixture of diastereomers; ^{1}H NMR (500 MHz, $CDCl_3$) ***Syn***: δ 7.36 – 7.17 (m, 9H), 5.43 (s, 1H), 5.14 (s, 1H), 4.62 (d, ***J* = 5.0 Hz**, 1H), 2.94 (dd, *J* = 9.7, 5.0 Hz, 1H), 2.08 (brs, 1H), 1.70 – 1.55 (m, 2H), 1.37 – 1.12 (m, 12H), 0.89 (t, *J* = 7.0, 7.0 Hz, 3H), ***Anti*** (assignable peaks only): δ 5.49 (d, *J* = 1.0 Hz, 1H), 5.25 (d, *J* = 1.0 Hz, 1H); ^{13}C NMR (101 MHz, $CDCl_3$) ***Syn***: δ 149.62, 143.12, 141.19, 132.67, 128.30, 128.06, 127.71, 127.42, 126.54, 114.35, 75.03, 51.96, 31.85, 29.79, 29.41, 29.24, 27.63, 27.35, 22.64, 14.09, ***Anti*** (assignable peaks only): δ 150.17, 142.84, 141.10, 133.32, 128.39, 126.92, 115.49, 53.46, 31.78, 31.14, 29.49, 29.29, 29.13,

27.03; HRMS calcd for $C_{26}H_{37}O$ $[M+H]^+$: 365.2844, found 365.2841.

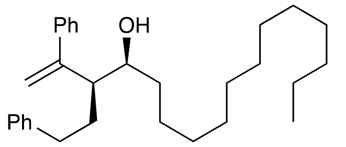

4g”

Isolated as a mixture of diastereomers; ^{1}H NMR (400 MHz, $CDCl_3$) ***Syn***: δ 7.46 – 7.11 (m, 9H), 5.42 (s, 1H), 5.13 (s, 1H), 4.60 (d, ***J* = 5.5 Hz**, 1H), 2.99 – 2.87 (m, 1H), 2.11 (brd, *J* = 8.5 Hz, 1H), 1.70 – 1.54 (m, 2H), 1.25 (d, *J* = 16.9 Hz, 20H), 0.90 (t, *J* = 6.8 Hz, 3H), ***Anti*** (assignable peaks only): δ 5.48 (s, 1H), 5.24 (s, 1H), 0.91 (t, *J* = 6.6 Hz, 24H); ^{13}C NMR (101 MHz, $CDCl_3$) ***Syn***: δ 149.55, 143.05, 141.13, 132.60, 128.21, 127.96, 127.64, 127.31, 126.45, 114.25, 74.99, 51.89, 31.84, 29.72, 29.58, 29.56, 29.51, 29.39, 29.27, 27.64, 27.62, 27.29, 22.61, 14.03, ***Anti*** (assignable peaks only): δ 150.08, 142.77, 141.03, 133.23, 128.30, 126.84, 115.38, 53.36, 31.05, 27.64, 26.96; HRMS calcd for $C_{30}H_{45}O$ $[M+H]^+$: 421.3470, found 421.3475.

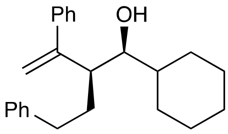

4h

Isolated as a mixture of diastereomers; ^{1}H NMR (500 MHz, $CDCl_3$) δ 7.37 – 7.25 (m, 7H), 7.19 (dd, *J* = 7.8, 6.2 Hz, 3H), 5.54 (s, 1H), 5.23 (s, 1H), 3.12 (d, *J* = 5.9 Hz, 1H), 2.87 (ddd, *J* = 13.9, 9.6, 4.5 Hz, 1H), 2.82 (dt, *J* = 11.4, 3.8 Hz, 1H), 2.58 (ddd, *J* = 13.8, 9.4, 7.8 Hz, 1H), 2.09 – 1.90 (m, 2H), 1.82 (d, *J* = 12.9 Hz, 1H), 1.70 – 1.59 (m, 4H), 1.52 – 1.39 (m, 2H), 1.25 – 1.13 (m, 1H), 1.16 – 0.96 (m, 2H), 0.92 – 0.78 (m, 2H); ^{13}C NMR (126 MHz, $CDCl_3$) δ 150.03, 142.77, 142.46, 128.44, 128.37, 128.29, 127.52, 126.62, 125.78, 114.14, 114.08, 45.49, 39.92, 33.41, 29.70, 29.26, 28.91, 28.49, 26.35, 26.17, 25.88; HRMS calcd for $C_{24}H_{31}O$ $[M+H]^+$: 335.2375, found 335.2385.

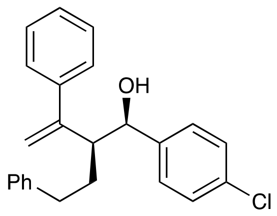

4i

Isolated as a mixture of diastereomers; ^{1}H NMR (500 MHz, $CDCl_3$) ***Syn***: δ 7.37 – 7.20 (m, 10H), 7.18 (d, *J* = 8.4 Hz, 2H), 7.11 (d, *J* = 8.2 Hz, 2H), 5.55 (s, 1H), 5.27 (s, 1H), 4.64 (d, ***J* = 4.9 Hz**, 1H), 3.00 (dd, *J* = 9.9, 4.7 Hz, 1H), 2.83 (ddd, *J* = 14.0, 8.9, 5.4 Hz, 1H), 2.59 – 2.49 (m, 1H), 2.14 (s,

1H), 2.08 – 1.94 (m, 2H), ***Anti*** (assignable peaks inly): δ 7.48 – 7.42 (m, 2H), 7.02 – 6.97 (m, 2H), 5.59 (s, 1H), 5.32 (s, 1H), 4.67 (d, *J* = 8.5 Hz, 1H), 2.96 – 2.91 (m, 1H), 2.70 (dt, *J* = 14.2, 7.1 Hz, 1H), 2.47 – 2.32 (m, 2H), 1.72 – 1.58 (m, 2H), 1.35 – 1.30 (m, 1H); ^{13}C NMR (126 MHz, $CDCl_3$) ***Syn***: δ 149.14, 142.71, 141.98, 140.87, 132.65, 128.36, 128.29, 128.09, 127.99, 127.53, 126.57, 126.47, 114.65, 114.58, 74.73, 74.67, 51.03, 50.95, 33.26, 29.02, ***Anti*** (assignable peaks only): δ 149.64, 142.53, 141.72, 140.63, 133.36, 126.91, 115.86, 115.83, 76.50, 52.65, 33.19, 32.60; HRMS calcd for $C_{24}H_{24}ClO$ $[M+H]^+$: 363.1516, found 363.1520.

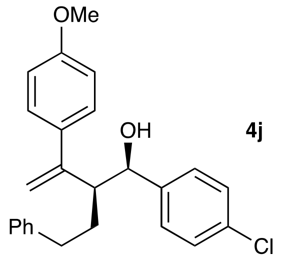

4j

Isolated as a mixture of diastereomers; ^{1}H NMR (500 MHz, $CDCl_3$) ***Syn***: δ 7.33 – 7.13 (m, 9H), 7.07 (d, *J* = 7.1 Hz, 2H), 6.85 (d, *J* = 8.7 Hz, 2H), 5.47 (s, 1H), 5.18 (s, 1H), 4.62 (d, ***J* = 4.8 Hz**, 1H), 3.83 (s, 3H), 2.93 (dd, *J* = 13.8, 5.4 Hz, 1H), 2.77 (dq, *J* = 14.4, 7.3 Hz, 1H), 2.50 (dt, *J* = 13.8, 8.5 Hz, 1H), 2.10 (s, 1H), 2.01 – 1.89 (m, 2H), ***Anti*** (assignable peaks only): zδ 7.36 (d, *J* = 8.7 Hz, 2H), 6.97 (d, *J* = 7.4 Hz, 2H), 6.90 (d, *J* = 8.7 Hz, 2H), 5.52 (s, 1H), 5.23 (s, 1H), 4.65 (d, ***J* = 8.6 Hz**, 1H), 3.85 (s, 3H), 2.65 (ddd, *J* = 14.3, 9.1, 5.7 Hz, 1H), 2.42 – 2.29 (m, 3H); ^{13}C NMR (126 MHz, $CDCl_3$) ***Syn***: δ 159.09, 148.41, 141.96, 140.87, 134.97, 132.54, 128.27, 128.18, 127.97, 127.56, 127.48, 125.67, 113.64, 74.55, 55.19, 50.87, 33.17, 28.81, ***Anti*** (assignable peaks only): δ 159.11, 148.95, 141.72, 140.66, 134.86, 133.29, 128.31, 128.16, 128.13, 127.94, 113.12, 76.55, 52.59, 33.12, 32.62, 29.59; HRMS calcd for $C_{25}H_{26}ClO_2$ $[M+H]^+$: 393.1621, found 393.1618.

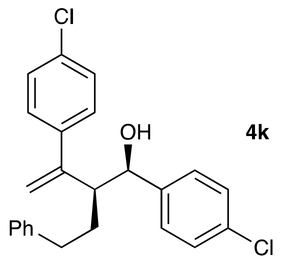

4k

Isolated as a mixture of diastereomers; ^{1}H NMR (500 MHz, $CDCl_3$) ***Syn***: δ 7.18 – 7.00 (m, 7H), 7.03 – 6.96 (m, 2H), 6.97 – 6.88 (m, 4H), 5.34 (s, 1H), 5.09 (s, 1H), 4.43 (d, *J* = 5.3 Hz, 1H), 2.74 (ddd, *J* = 10.8, 5.4, 3.4 Hz, 1H), 2.63 (ddd, *J* = 14.0, 9.8, 4.5 Hz, 1H), 2.37 (ddd, *J*

= 13.8, 9.4, 7.5 Hz, 1H), 2.00 – 1.87 (m, 2H), 1.87 – 1.75 (m, 1H); ***Anti*** (assignable peaks only): δ 6.90 (d, *J* = 7.1 Hz, 2H), 5.47 (s, 1H), 5.24 (s, 1H), 4.55 (d, *J* = 8.4 Hz, 1H), 2.58 (dt, *J* = 14.4, 7.4 Hz, 1H), 2.32 (dt, *J* = 13.7, 8.3 Hz, 1H); ^{13}C NMR (126 MHz, $CDCl_3$) ***Syn***: δ 148.21, 141.88, 141.35, 140.88, 133.42, 132.94, 128.49, 128.40, 128.19, 127.88, 127.74, 125.93, 115.21, 115.17, 75.28, 51.06, 33.28, 29.64, ***Anti*** (assignable peaks only) : δ 148.79, 141.59, 141.20, 140.69, 133.59, 133.49, 128.53, 128.33, 128.32, 128.29, 116.09, 116.06, 76.68, 52.36, 33.20, 29.76; HRMS calcd for $C_{24}H_{23}Cl_2O$ $[M+H]^+$: 397.1126, found 397.1130.

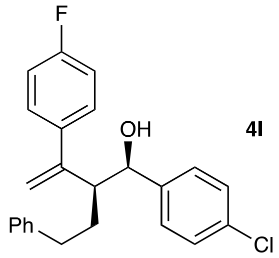

4l

Isolated as a mixture of diastereomers; ^{1}H NMR (500 MHz, $CDCl_3$) ***Syn***: δ 7.30 – 7.13 (m, 6H), 7.10 (d, *J* = 8.4 Hz, 2H), 7.04 (d, *J* = 7.0 Hz, 2H), 6.93 (td, *J* = 8.5, 2.6 Hz, 1H), 6.94 – 6.88 (m, 1H), 6.83 (dt, *J* = 10.3, 2.1 Hz, 1H), 5.49 (s, 1H), 5.22 (s, 1H), 4.56 (d, ***J* = 5.3 Hz,** 1H), 2.86 (ddd, *J* = 11.0, 5.4, 3.4 Hz, 1H), 2.74 (ddd, *J* = 14.1, 9.9, 4.6 Hz, 1H), 2.48 (ddd, *J* = 13.8, 9.6, 7.5 Hz, 1H), 2.03 (dddd, *J* = 13.5, 10.4, 7.4, 3.4 Hz, 1H), 1.92 (dtd, *J* = 14.0, 10.1, 4.6 Hz, 1H), 1.26 (brd, *J* = 5.3 Hz, 1H); ***Anti*** (assignable peaks only): δ 5.53 (s, 1H), 5.28 (s, 1H), 4.60 (d, ***J* = 8.2 Hz**, 1H), 2.62 (dt, *J* = 14.4, 7.5 Hz, 1H), 2.36 (dt, *J* = 13.8, 8.3 Hz, 1H), 1.64 (p, *J* = 8.0 Hz, 2H); ^{13}C NMR (126 MHz, $CDCl_3$) ***Syn***: δ 162.73 (d, *J* = 245.9 Hz), 148.21 (d, *J* = 2.1 Hz), 145.24 (d, *J* = 7.3 Hz), 141.87, 140.84, 132.94, 129.81 (d, *J* = 8.2 Hz), 128.39, 128.17, 127.74, 125.93, 122.23 (d, *J* = 2.8 Hz), 115.58, 114.34 (d, *J* = 21.2 Hz), 113.57 (d, *J* = 21.9 Hz), 75.28, 51.04, 33.28, 29.62; ***Anti***: δ 162.76 (d, *J* = 245.6 Hz), 148.79 (d, *J* = 2.0 Hz), 145.11 (d, *J* = 7.4 Hz), 141.60, 140.67, 133.56, 129.85 (d, *J* = 8.3 Hz), 128.45, 128.29, 127.87, 125.91, 122.65 (d, *J* = 2.8 Hz), 116.38, 114.38 (d, *J* = 21.1 Hz), 113.97 (d, *J* = 21.9 Hz), 76.68, 52.30, 33.21, 32.79; HRMS calcd for $C_{24}H_{23}ClFO$ $[M+H]^+$: 381.1421, found 381.1418.

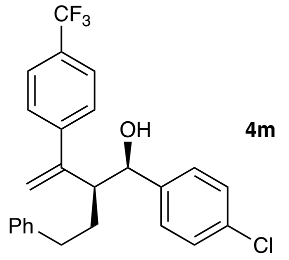

4m

Isolated as a mixture of diastereomers; ^{1}H NMR (500 MHz, $CDCl_3$) ***Syn***: δ 7.42 (d, *J* = 8.1 Hz, 2H), 7.16 (d, *J* = 7.3 Hz, 2H), 7.14 – 7.06 (m, 5H), 7.02 (d, *J* = 8.4 Hz, 2H), 6.97 (d, *J* = 7.0 Hz, 2H), 5.44 (s, 1H), 5.20 (s, 1H), 4.48 (dd, ***J* = 5.7, 1.6 Hz**, 1H), 2.82 (ddd, *J* = 10.6, 5.4, 3.3 Hz, 1H), 2.68 (ddd, *J* = 14.0, 9.8, 4.5 Hz, 1H), 2.43 (ddd, *J* = 13.8, 9.3, 7.8 Hz, 1H), 2.03 (dddd, *J* = 13.3, 9.8, 7.6, 3.4 Hz, 1H), 1.94 (brd, *J* = 2.7 Hz, 1H), 1.87 (dddd, *J* = 13.9, 11.0, 9.5, 4.6 Hz, 1H), ***Anti***: δ 7.57 (d, *J* = 8.2 Hz, 2H), 7.45 (d, *J* = 8.1 Hz, 2H), 6.93 (d, *J* = 7.1 Hz, 2H), 5.56 (s, 1H), 5.35 (s, 1H), 4.61 (dd, ***J* = 8.2, 2.1 Hz**, 1H), 2.64 (dt, *J* = 14.6, 7.5 Hz, 1H), 2.38 (dt, *J* = 14.2, 8.4 Hz, 1H), 2.18 (d, *J* = 3.0 Hz, 1H), 1.66 (td, *J* = 8.3, 6.9 Hz, 2H); ^{13}C NMR (126 MHz, $CDCl_3$) ***Syn***: δ 148.24, 146.55, 141.69, 140.71, 133.03, 129.43 (q, *J* = 32.4 Hz), 128.36, 128.34, 128.15, 127.73, 127.26, 126.83, 125.92, 125.21 (q, *J* = 4.1 Hz), 116.38, 75.51, 50.93, 33.18, 29.81, ***Anti***: δ 148.85, 146.44, 141.38, 140.61, 133.60, 128.46, 128.30, 128.22, 128.20, 127.26, 123.01, 117.01, 76.66, 52.12, 32.71, 21.43; HRMS calcd for $C_{25}H_{23}ClF_3O$ $[M+H]^+$: 431.1390, found 431.1388.

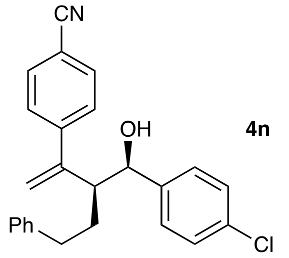

4n

Isolated as a mixture of diastereomers; ^{1}H NMR (500 MHz, $CDCl_3$) ***Syn***: δ 7.44 (d, *J* = 8.4 Hz, 2H), 7.21 (t, *J* = 7.3 Hz, 2H), 7.18 – 7.06 (m, 5H), 7.07 – 7.00 (m, 4H), 5.47 (s, 1H), 5.26 (s, 1H), 4.50 (dd, *J* = **6.2, 2.4** Hz, 1H), 2.83 (ddd, *J* = 10.1, 6.1, 3.3 Hz, 1H), 2.72 (ddd, *J* = 14.1, 9.9, 4.5 Hz, 1H), 2.48 (ddd, *J* = 13.8, 9.5, 7.4 Hz, 1H), 2.21 – 2.10 (m, 2H), 1.90 (dddd, *J* = 14.0, 11.0, 9.6, 4.5 Hz, 1H), ***Anti***: δ 7.56 (d, *J* = 8.4 Hz, 2H), 7.42 (d, *J* = 8.4 Hz, 2H), 6.92 (d, *J* = 6.9 Hz, 2H), 5.57 (s, 1H), 5.39 (s, 1H), 4.61 (dd, *J* = **8.0, 2.5** Hz, 1H), 2.62 (dt, *J* = 14.3, 6.7 Hz, 1H), 2.40 – 2.27 (m, 1H), 2.29 (dd, *J* = 5.7, 3.1 Hz, 1H); ^{13}C NMR (126 MHz, $CDCl_3$) ***Syn***: δ 147.93, 147.68, 141.53, 140.71, 133.05, 131.94, 128.32, 128.26, 128.08, 127.81, 127.05, 125.90, 118.69, 117.12, 110.73, 76.09, 50.79, 33.14, 30.59, ***Anti***: δ 148.58, 141.20, 140.64, 133.54, 132.02, 128.40, 128.14, 127.48,

118.76, 117.41, 110.82, 51.53, 32.88, 29.62; HRMS calcd for $C_{25}H_{23}ClNO$ [M+H]$^+$: 388.1468, found 388.1466.

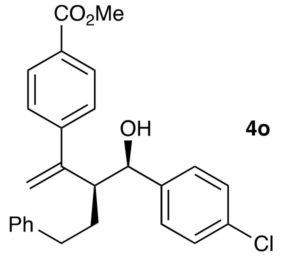

4o

Isolated as a mixture of diastereomers; ^{1}H NMR (500 MHz, $CDCl_3$) ***Syn***: δ 7.87 (d, *J* = 8.3 Hz, 2H), 7.24 – 7.16 (m, 2H), 7.17 – 7.10 (m, 5H), 7.06 (d, *J* = 8.4 Hz, 2H), 7.01 (d, *J* = 6.8 Hz, 2H), 5.50 (s, 1H), 5.24 (s, 1H), 4.52 (d, *J* = **5.4** Hz, 1H), 3.85 (s, 3H), 2.94 – 2.81 (m, 1H), 2.71 (ddd, *J* = 14.1, 9.9, 4.5 Hz, 1H), 2.46 (ddd, *J* = 13.8, 9.6, 7.4 Hz, 1H), 2.13 – 1.97 (m, 1H), 1.90 (dddd, *J* = 13.9, 10.8, 9.6, 4.5 Hz, 1H), 1.18 (d, *J* = 6.9 Hz, 1H), ***Anti*** (assignable peaks only): δ 7.94 (d, *J* = 8.3 Hz, 1H), 7.38 (d, *J* = 8.4 Hz, 1H), 6.91 – 6.85 (m, 1H), 5.54 (s, 1H), 5.30 (s, 1H), 4.58 (d, *J* = **8.1** Hz, 1H), 3.87 (s, 2H), 2.58 (dt, *J* = 14.4, 7.5 Hz, 1H), 2.32 (dt, *J* = 13.7, 8.4 Hz, 1H), 0.95 (d, *J* = 6.7 Hz, 1H); ^{13}C NMR (126 MHz, $CDCl_3$) ***Syn***: δ 166.67, 148.40, 147.36, 141.65, 140.66, 132.79, 129.49, 128.90, 128.21, 128.01, 127.58, 126.36, 125.75, 116.12, 75.26, 51.99, 50.78, 33.10, 29.63, ***Anti*** (assignable peaks only): δ 166.73, 148.97, 147.30, 141.36, 140.50, 133.40, 129.53, 128.96, 128.29, 128.16, 128.10, 126.77, 125.74, 116.82, 76.53, 52.01, 51.96, 33.02, 32.65; HRMS calcd for $C_{26}H_{26}ClO_3$ [M+H]$^+$: 421.1570, found 421.1565.

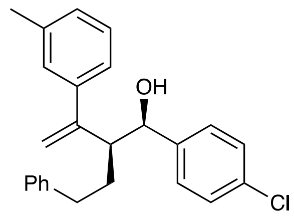

4p

Isolated as a mixture of diastereomers; ^{1}H NMR (500 MHz, $CDCl_3$) ***Syn***: δ 7.33 – 7.15 (m, 9H), 7.11 (t, *J* = 7.8 Hz, 2H), 7.03 (d, *J* = 8.0 Hz, 1H), 6.98 (s, 1H), 5.52 (s, 1H), 5.24 (s, 1H), 4.62 (t, *J* = 4.6, 1.6 Hz, 1H), 2.96 (dt, *J* = 9.7, 4.9 Hz, 1H), 2.82 (ddd, *J* = 14.1, 8.6, 5.8 Hz, 1H), 2.54 (dt, *J* = 14.1, 8.5 Hz, 1H), 2.36 (s, 3H), 2.10 (s, 1H), 2.05 – 1.93 (m, 2H), ***Anti*** (assignable peaks only): δ 5.57 (s, 1H), 5.29 (s, 1H), 4.67 (dd, *J* = 8.2, 2.3 Hz, 1H), 2.70 (dt, *J* = 14.4, 7.4 Hz, 1H), 2.41 (s, 4H), 1.67 (q, *J* = 7.9 Hz, 2H); ^{13}C NMR (126 MHz, $CDCl_3$) ***Syn***: δ 149.26, 142.72, 141.95, 140.82, 137.82, 132.57, 128.31, 128.20, 128.13, 127.96, 127.54, 127.36, 125.70, 123.48, 114.27, 74.62,

51.01, 33.18, 28.83, 21.34, ***Anti*** (assignable peaks only): δ 149.73, 142.55, 141.79, 140.67, 133.36, 128.40, 128.31 (d, *J* = 2.3 Hz), 128.21, 127.64, 124.00, 115.64, 52.58, 33.21, 32.62, 21.49; HRMS calcd for $C_{25}H_{26}ClO$ $[M+H]^+$: 377.1672, found 377.1669.

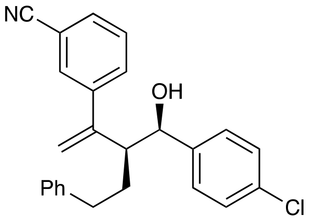

4q

Isolated as a mixture of diastereomers; ^1H NMR (500 MHz, $CDCl_3$) ***Syn***: δ 7.33 (t, *J* = 7.7 Hz, 1H), 7.32 – 7.24 (m, 4H), 7.21 (ddt, *J* = 15.3, 9.0, 2.0 Hz, 4H), 7.10 (dd, *J* = 7.8, 5.7 Hz, 4H), 5.50 (s, 1H), 5.30 (s, 1H), 4.57 (d, ***J* = 6.1 Hz**, 1H), 2.87 (ddd, *J* = 10.4, 6.3, 3.5 Hz, 1H), 2.79 (ddd, *J* = 14.1, 9.8, 4.5 Hz, 1H), 2.56 (ddd, *J* = 13.8, 9.4, 7.5 Hz, 1H), 2.22 (dddd, *J* = 13.5, 9.5, 7.5, 3.4 Hz, 1H), 2.17 – 2.07 (m, 1H), 1.96 (dddd, *J* = 13.9, 11.0, 9.4, 4.6 Hz, 1H); ***Anti*** (assignable peaks only): δ 7.61 (d, *J* = 1.8 Hz, 1H), 7.59 – 7.54 (m, 2H), 7.42 (t, *J* = 7.8 Hz, 1H), 6.96 (d, *J* = 6.9 Hz, 2H), 5.56 (s, 1H), 5.39 (s, 1H), 4.65 (d, ***J* = 8.0 Hz**, 1H), 2.65 (dt, *J* = 14.3, 7.3 Hz, 1H), 2.41 (dt, *J* = 13.8, 8.3 Hz, 1H), 1.76 – 1.67 (m, 2H); ^{13}C NMR (126 MHz, $CDCl_3$) ***Syn***: δ 147.49, 144.34, 141.49, 140.67, 133.16, 130.79, 130.66, 130.12, 128.98, 128.38, 128.33, 128.13, 127.83, 126.01, 118.58, 116.63, 112.29, 76.15, 50.97, 33.15, 30.44; ***Anti*** (assignable peaks only): δ 148.13, 144.26, 141.18, 133.61, 131.27, 130.77, 130.51, 129.06, 128.45, 128.19, 128.11, 127.83, 126.01, 125.97, 117.04, 76.15, 51.74, 32.85, 31.53; HRMS calcd for $C_{25}H_{23}ClNO$ $[M+H]^+$: 388.1468, found 388.1466.

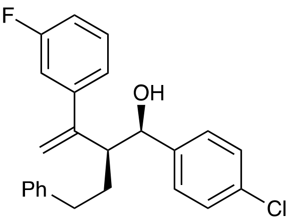

4r

Isolated as a mixture of diastereomers; ^1H NMR (500 MHz, $CDCl_3$) ***Syn***: δ 7.30 – 7.13 (m, 6H), 7.10 (d, *J* = 8.4 Hz, 2H), 7.04 (d, *J* = 7.0 Hz, 2H), 6.92 (t, *J* = 6.6 Hz, 2H), 6.83 (dt, *J* = 10.3, 2.1 Hz, 1H), 5.49 (s, 1H), 5.22 (s, 1H), 4.56 (d, *J* = 5.3 Hz, 1H), 2.86 (ddd, *J* = 11.0, 5.4, 3.4 Hz, 1H), 2.74 (ddd, *J* = 14.1, 9.9, 4.6 Hz, 1H), 2.48 (ddd, *J* = 13.8, 9.6, 7.5 Hz, 1H), 2.03 (dddd, *J* = 13.5, 10.4, 7.4, 3.4 Hz, 1H), 1.92 (dtd, *J* = 14.0, 10.1, 4.6 Hz, 1H), ***Anti***

(assignable peaks only): δ 5.53 (s, 1H), 5.28 (s, 1H), 4.60 (d, *J* = 8.2 Hz, 1H), 2.62 (dt, *J* = 14.4, 7.5 Hz, 1H), 2.36 (dt, *J* = 13.8, 8.3 Hz, 1H), 1.63 (q, *J* = 7.8 Hz, 1H); ^{13}C NMR (126 MHz, $CDCl_3$) ***Syn***: δ 162.73 (d, *J* = 245.9 Hz), 148.21 (d, *J* = 2.0 Hz), 145.24 (d, *J* = 7.3 Hz), 141.87, 140.84, 132.94, 129.84, 129.78, 128.39, 128.17, 127.74, 125.93, 122.23 (d, *J* = 2.8 Hz), 115.58, 114.34 (d, *J* = 21.2 Hz), 113.57 (d, *J* = 21.9 Hz), 75.28, 51.04, 33.28, 29.62, ***Anti***: δ 162.76 (d, *J* = 245.6 Hz), 148.79 (d, *J* = 2.0 Hz), 145.11 (d, *J* = 7.4 Hz), 141.60, 140.67, 133.56, 129.89, 129.82, 128.45, 128.35, 128.29, 127.88, 125.91, 122.23 (d, *J* = 2.8 Hz), 116.38, 114.38 (d, *J* = 21.1 Hz), 113.97 (d, *J* = 21.9 Hz), 76.68, 52.30, 33.21, 32.79; HRMS calcd for $C_{24}H_{23}ClFO$ $[M+H]^+$: 381.1421, found 381.1420.

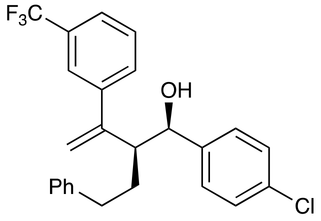

4s

Isolated as a mixture of diastereomers; ^{1}H NMR (500 MHz, $CDCl_3$) ***Syn***: δ 7.38 – 7.19 (m, 11H), 7.18 (d, *J* = 8.4 Hz, 2H), 7.11 (d, *J* = 8.2 Hz, 2H), 5.55 (s, 1H), 5.27 (s, 1H), 4.64 (d, ***J* = 4.9 Hz**, 1H), 2.99 (dt, *J* = 9.6, 4.7, 4.7 Hz, 1H), 2.88 – 2.77 (m, 1H), 2.59 – 2.49 (m, 1H), 2.18 – 2.10 (m, 1H), 2.09 – 1.94 (m, 2H), ***Anti*** (assignable peaks only): δ 5.59 (s, 1H), 5.32 (s, 1H), 4.67 (d, ***J* = 8.5 Hz**, 1H), 2.98 – 2.89 (m, 1H), 2.75 – 2.64 (m, 1H), 2.47 – 2.33 (m, 2H), 1.72 – 1.65 (m, 1H), 1.63 (brs, 1H), 1.36 – 1.30 (m, 1H); ^{13}C NMR (126 MHz, $CDCl_3$) ***Syn***: δ 149.14, 142.71, 141.98, 140.87, 132.65, 128.36, 128.29, 128.09, 127.99, 127.53, 126.57, 126.47, 114.65, 114.58, 74.73, 74.67, 51.03, 50.95, 33.26, 29.02, ***Anti*** (assignable peaks only): δ 149.64, 142.53, 141.72, 140.63, 133.36, 126.91, 115.86, 115.83, 76.50, 52.65, 33.19, 32.60; HRMS calcd for $C_{24}H_{23}ClF_3O$ $[M+H]^+$: 431.1390, found 431.1388.

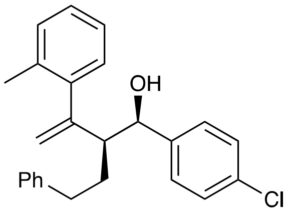

4t

Isolated as a mixture of diastereomers; ^{1}H NMR (500 MHz, $CDCl_3$) ***Syn***: δ 7.49 (dd, *J* = 6.9, 2.4 Hz, 1H), 7.34 – 7.25 (m, 5H), 7.25 – 7.19 (m, 3H), 7.16 (td, *J* = 4.6, 2.1 Hz, 2H), 7.12 –

7.05 (m, 3H), 5.49 (d, *J* = 1.0 Hz, 1H), 5.35 (s, 1H), 4.80 (d, *J* = 4.5 Hz, 1H), 2.99 (dt, *J* = 10.6, 4.2 Hz, 1H), 2.81 (td, *J* = 9.5, 4.9 Hz, 1H), 2.53 (ddd, *J* = 14.5, 10.0, 7.7 Hz, 1H), 2.17 – 2.05 (m, 2H), 2.02 (s, 3H); ^{13}C NMR (126 MHz, $CDCl_3$) ***Syn***: δ 150.23, 142.99, 142.24, 140.32, 134.32, 130.34, 128.39, 128.30, 128.23, 127.48, 127.00, 126.86, 126.58, 125.75, 125.69, 114.43, 71.58, 48.94, 33.41, 28.40, 19.09; HRMS calcd for $C_{25}H_{26}ClO$ $[M+H]^+$: 377.1672, found 377.1669.

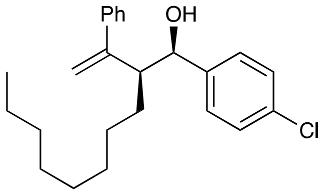

4u

Isolated as a mixture of diastereomers; ^{1}H NMR (500 MHz, $CDCl_3$) ***Syn***: δ 7.52 (d, *J* = 8.1 Hz, 2H), 7.35 – 7.27 (m, 5H), 7.28 – 7.16 (m, 5H), 7.02 (d, *J* = 6.9 Hz, 2H), 5.55 (s, 1H), 5.27 (s, 1H), 4.65 (d, *J* = **4.5 Hz**, 1H), 2.95 (dt, *J* = 11.2, 3.9 Hz, 1H), 2.77 (ddd, *J* = 13.9, 9.5, 4.4 Hz, 1H), 2.48 (dt, *J* = 13.9, 8.6 Hz, 1H), 2.14 (s, 1H), 1.98 (dddd, *J* = 13.7, 11.0, 9.1, 4.4 Hz, 1H), 1.86 (dddd, *J* = 13.6, 9.3, 7.8, 3.3 Hz, 1H), ***Anti*** (assignable peaks only): δ 7.56 (d, *J* = 8.0 Hz, 2H), 7.38 (d, *J* = 8.0 Hz, 2H), 6.95 (d, *J* = 6.9 Hz, 2H), 4.72 (d, *J* = **7.7 Hz**, 1H), 2.68 (ddd, *J* = 14.3, 9.6, 5.2 Hz, 1H), 1.75 – 1.64 (m, 1H), ^{13}C NMR (126 MHz, $CDCl_3$) ***Syn***: δ 148.78, 147.80, 142.37, 141.71, 131.80, 128.49, 128.35, 128.32, 127.79, 126.92, 126.51, 125.92, 118.90, 114.89, 110.77, 74.53, 50.74, 33.13, 28.51, ***Anti*** (assignable peaks only): δ 148.93, 147.53, 142.30, 141.49, 131.92, 128.42, 128.24, 127.71, 127.67, 126.82, 118.78, 116.29, 111.39, 76.14, 52.32, 32.54, 29.69; HRMS calcd for $C_{24}H_{32}ClO$ $[M+H]^+$: 371.2142, found 371.2139.

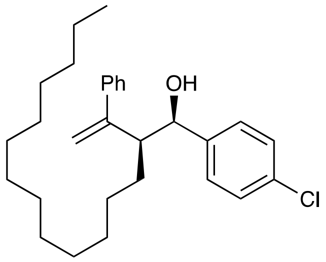

4u'

Isolated as a mixture of diastereomers; ^{1}H NMR (400 MHz, $CDCl_3$) ***Syn***: δ 7.47 – 7.11 (m, 9H), 5.42 (s, 1H), 5.13 (s, 1H), 4.60 (d, *J* = **5.0** Hz, 1H), 2.93 (dt, *J* = 8.7, 5.5 Hz, 1H), 2.11 (d, *J* = 8.5 Hz, 1H), 1.71 – 1.55 (m, 2H), 1.25 (d, *J* = 16.9 Hz, 20H), 0.91 (t, *J* = 6.9 Hz, 3H), ***Anti*** (assignable peaks only): δ 5.48 (s, 1H), 5.24 (s, 1H), 2.85 (q, *J* = 9.1, 8.4 Hz, 1H), 2.36 (brs, 1H); ^{13}C NMR

(101 MHz, $CDCl_3$) ***Syn***: δ 149.59, 143.10, 141.18, 132.64, 128.25, 128.01, 127.69, 127.36, 126.49, 114.30, 75.05, 51.94, 31.89, 29.76, 29.62, 29.60, 29.56, 29.44, 29.31, 27.34, 22.65, 14.07, ***Anti*** (assignable peaks only): δ 150.12, 142.82, 141.07, 133.28, 128.35, 126.88, 53.40; HRMS calcd for $C_{29}H_{42}ClO$ $[M+H]^+$: 441.2924, found 441.2922.

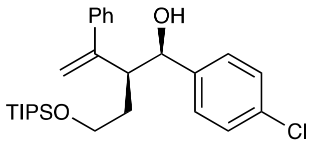

4v

Isolated as a mixture of diastereomers; ^{1}H NMR (500 MHz, $CDCl_3$) ***Syn***: δ 7.30 – 7.15 (m, 9H), 5.37 (d, *J* = 0.9 Hz, 1H), 5.18 (s, 1H), 4.72 (dd, *J* = **5.5, 1.9 Hz**, 1H), 3.80 (dt, *J* = 10.1, 5.0 Hz, 1H), 3.68 (ddd, *J* = 10.1, 8.1, 4.9 Hz, 1H), 3.29 (d, *J* = 2.5 Hz, 1H), 3.20 (dt, *J* = 7.6, 5.5 Hz, 1H), 1.96 – 1.80 (m, 2H), 1.63 (d, *J* = 1.8 Hz, 1H), 1.03 (d, *J* = 5.3 Hz, 18H), ***Anti*** (assignable peaks only): δ 5.46 (s, 1H), 5.22 (s, 1H), 4.68 (dd, *J* = **7.9, 2.0 Hz,** 1H), 3.61 – 3.52 (m, 1H); ^{13}C NMR (126 MHz, $CDCl_3$) ***Syn***: δ S149.92, 142.75, 141.39, 132.47, 128.16, 127.90, 127.84, 127.33, 126.46, 114.35, 74.93, 61.23, 49.03, 32.00, 17.87, 11.81; ***Anti*** (assignable peaks only): δ 149.69, 142.75, 140.76, 133.09, 128.32, 128.09, 126.70, 115.45; HRMS calcd for $C_{27}H_{40}ClO_2Si$ $[M+H]^+$: 459.2486, found 459.2490.

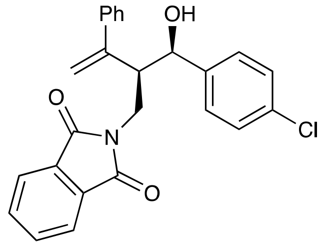

syn-**4w**

^{1}H NMR (500 MHz, $CDCl_3$) δ 7.81 – 7.71 (m, 2H), 7.73 – 7.64 (m, 2H), 7.32 – 7.17 (m, 7H), 7.13 (d, *J* = 8.3 Hz, 2H), 5.38 (s, 1H), 5.27 (s, 1H), 4.77 (t, *J* = **5.3 Hz**, 1H), 4.12 (dd, *J* = 14.2, 7.3 Hz, 1H), 3.99 (dd, *J* = 14.2, 6.8 Hz, 1H), 3.84 (q, *J* = 6.8 Hz, 1H), 2.98 – 2.91 (m, 1H); ^{13}C NMR (126 MHz, $CDCl_3$) δ 168.75, 147.13, 141.95, 140.26, 133.93, 132.81, 131.70, 128.35, 128.14, 127.62, 127.44, 126.49, 123.10, 115.68, 73.41, 48.61, 37.75; HRMS calcd for $C_{25}H_{21}ClNO_3$ $[M+H]^+$: 418.1210, found 418.1208.

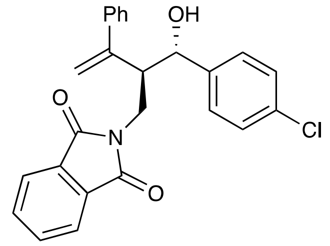

anti-**4w**

^{1}H NMR (500 MHz, $CDCl_3$) δ 7.77 (dd, *J* = 5.5, 3.1 Hz, 2H), 7.69 (dd, *J* = 5.5, 3.1 Hz, 2H), 7.28 – 7.18 (m, 7H), 7.12 (d, *J* = 8.3 Hz, 2H), 5.48 (s, 2H), 4.81 (dd, ***J* = 6.4, 3.9 Hz**, 1H), 3.94 (dd, *J* = 14.0, 7.0 Hz, 1H), 3.85 (dd, *J* = 14.0, 8.8 Hz, 1H), 3.67 (td, *J* = 8.8, 7.0, 6.4 Hz, 1H), 2.94 (d, *J* = 3.9 Hz, 1H); ^{13}C NMR (126 MHz, $CDCl_3$) δ 168.59, 146.19, 142.39, 139.56, 134.09, 133.15, 131.66, 128.22, 128.16, 127.90, 127.39, 126.27, 123.23, 116.53, 73.35, 48.96, 40.07; HRMS calcd for $C_{25}H_{21}ClNO_3$ $[M+H]^+$: 418.1210, found 418.1205.

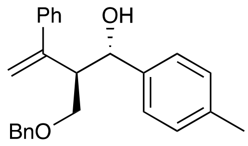

7

Isolated as a mixture of diastereomers; ^{1}H NMR (400 MHz, $CDCl_3$) ***Anti***: δ 7.46 – 7.29 (m, 5H), 7.28 – 7.18 (m, 5H), 7.17 – 7.10 (m, 2H), 7.08 (d, *J* = 8.0 Hz, 2H), 5.35 (s, 1H), 5.27 (s, 1H), 4.95 (dd, *J* = **7.1, 3.4** Hz, 1H), 4.56 (s, 2H), 4.08 (d, *J* = 3.5 Hz, 1H), 3.91 – 3.77 (m, 3H), 3.24 (td, *J* = 6.8, 4.4 Hz, 1H), 2.32 (s, 3H); ***Syn*** (assignable peaks only): δ 5.42 (s, 1H), 5.07 (s, 1H), 4.90 (t, *J* = **5.7** Hz, 1H), 3.58 – 3.48 (m, 1H), 3.40 (q, *J* = 6.2 Hz, 1H), 2.36 (s, 3H); ^{13}C NMR (101 MHz, $CDCl_3$) ***Anti***: δ 147.42, 142.44, 139.83, 137.37, 136.64, 128.57, 128.43, 128.05, 127.81, 127.74, 127.19, 126.61, 126.44, 115.38, 77.27, 73.56, 72.59, 50.89, 21.05, ***Syn***: δ 147.12, 142.26, 138.53, 137.85, 136.89, 74.47, 73.28, 70.79, 51.27, 14.15, HRMS calcd for $C_{25}H_{27}O_2$ $[M+H]^+$: 359.2011, found 359.2015.

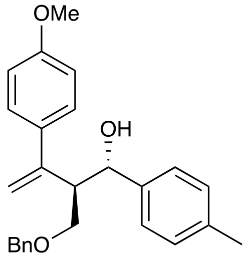

8

Isolated as a mixture of diastereomers; ^{1}H NMR (400 MHz, $CDCl_3$) ***Anti***: δ 7.41 – 7.25 (m, 5H), 7.22 – 7.14 (m, 2H), 7.05 (d, *J* = 8.6 Hz, 4H), 6.76 (d, *J* = 8.8 Hz, 2H), 5.26 (s, 1H), 5.15 (s, 1H), 4.92 (dd, *J* = **7.1, 3.4** Hz, 1H), 4.53 (s, 2H), 4.04 (d, *J* = 3.5 Hz, 1H), 3.87 – 3.73 (m,

2H), 3.78 (s, 3H), 3.17 (td, *J* = 6.9, 4.4 Hz, 1H), 2.29 (s, 3H), ***Syn*** (assignable peaks only): δ 7.10 (d, *J* = 8.4 Hz, 2H), 6.86 (d, *J* = 8.8 Hz, 2H), 5.33 (s, 0H), 4.85 (t, *J* = 5.9 Hz, 1H), 4.06 (d, *J* = 3.3 Hz, 1H), 3.56 – 3.42 (m, 1H), 3.35 (q, *J* = 5.9 Hz, 1H); ^{13}C NMR (101 MHz, $CDCl_3$) ***Anti***: δ 158.91, 146.76, 139.90, 137.42, 136.66, 134.87, 128.60, 128.47, 128.35, 127.85, 127.79, 127.56, 126.63, 114.13, 113.67, 113.43, 77.41, 73.61, 72.77, 55.22, 50.92, 21.08; ***Syn*** (assignable peaks only): δ 159.09, 146.47, 138.55, 137.88, 136.92, 134.61, 128.57, 128.35, 127.87, 127.63, 74.60, 73.34, 70.87, 55.28, 51.22, 21.13, HRMS calcd for $C_{26}H_{29}O_3$ $[M+H]^+$: 389.2117, found 389.2115.

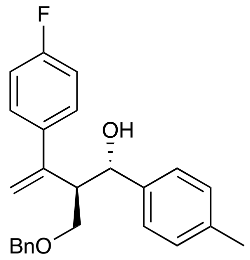

9

Isolated as a mixture of diastereomers; ^{1}H NMR (500 MHz, $CDCl_3$) ***Anti***: δ 7.39 – 7.28 (m, 5H), 7.16 (d, *J* = 8.1 Hz, 2H), 7.07 – 6.99 (m, 4H), 6.88 (t, *J* = 8.8 Hz, 2H), 5.26 (s, 1H), 5.21 (s, 1H), 4.88 (dd, *J* = **7.3, 3.4** Hz, 1H), 4.55 (s, 2H), 3.98 (d, *J* = 3.4 Hz, 1H), 3.84 (dd, *J* = 9.5, 6.6 Hz, 1H), 3.78 (dd, *J* = 9.5, 4.6 Hz, 1H), 3.14 (td, *J* = 7.0, 4.6 Hz, 1H), 2.29 (s, 3H), ***Syn***: δ5.35 (s, 1H), 5.07 (s, 1H), 4.85 (d, *J* = 5.6 Hz, 1H), 4.46 (d, *J* = 12.0 Hz, 1H), 4.41 (d, *J* = 12.0 Hz, 1H), 3.48 (dd, *J* = 6.1, 1.2 Hz, 2H), 3.29 (q, *J* = 6.4 Hz, 1H), 3.00 (d, *J* = 5.1 Hz, 1H), 2.33 (s, 3H); HRMS calcd for $C_{25}H_{26}FO_2$ $[M+H]^+$: 377.1917, found 377.1920.

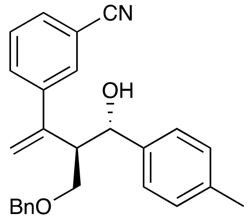

10

Isolated as a mixture of diastereomers; ^{1}H NMR (500 MHz, $CDCl_3$) ***Anti***: δ 7.49 – 7.43 (m, 2H), 7.41 – 7.29 (m, 9H), 7.14 – 7.09 (m, 2H), 7.02 (d, *J* = 7.8 Hz, 2H), 5.36 (s, 1H), 5.33 (s, 1H), 4.83 (dd, *J* = **7.6, 3.3** Hz, 1H), 4.58 (d, *J* = 12.0 Hz, 1H), 4.55 (d, *J* = 11.9 Hz, 1H), 3.88 (dd, *J* = 9.5, 6.6 Hz, 1H), 3.85 (dd, *J* = 3.4, 1.1 Hz, 1H), 3.81 (dd, *J* = 9.5, 5.0 Hz, 1H), 2.29 (s, 3H), ***Syn*** (assignable peaks only): δ 5.46 (s, 1H), 4.89 (dd, *J* = **6.4, 4.5** Hz, 1H), 4.46 (d, *J* = 11.9 Hz, 1H), 4.42 (d, *J* = 11.9 Hz, 1H), 3.53 (dd, *J* = 9.2, 6.3 Hz, 1H), 3.49 (dd, *J* = 9.2, 6.0

Hz, 1H), 3.23 (q, *J* = 6.2 Hz, 1H), 2.82 (ddt, *J* = 4.7, 2.8, 1.5 Hz, 1H), 2.33 (s, 3H); ^{13}C NMR (126 MHz, $CDCl_3$) ***Anti***: δ 147.32, 146.50, 139.24, 137.24, 137.04, 131.75, 128.63, 128.44, 127.73, 127.53, 127.08, 126.65, 118.79, 117.52, 110.55, 73.61, 72.44, 50.90, 20.99, ***Syn*** (assignable peaks only): δ 147.49, 146.28, 138.69, 137.72, 137.23, 131.88, 128.75, 128.63, 128.31, 127.89, 127.64, 127.43, 126.27, 118.86, 117.76, 110.59, 74.20, 73.26, 70.94, 51.43, 21.03; HRMS calcd for $C_{26}H_{26}NO_2$ $[M+H]^+$: 384.1964, found 384.1961.

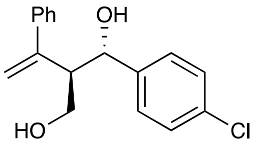

11

Isolated as a mixture of diastereomers; ^{1}H NMR (500 MHz, $CDCl_3$) ***Anti***: δ 7.41 – 7.15 (m, 7H), 7.12 – 6.98 (m, 2H), 5.34 (s, 1H), 5.18 (s, 1H), 4.92 (d, *J* = **8.2** Hz, 1H), 3.96 (h, *J* = 7.0 Hz, 2H), 3.62 (d, *J* = 6.0 Hz, 1H), 3.11 (td, *J* = 7.6, 4.2 Hz, 1H), 2.68 (s, 2H), ***Syn*** (assignable peaks only): δ 5.48 (s, 1H), 5.12 (s, 1H), 3.22 (q, *J* = 6.1 Hz, 1H); ^{13}C NMR (126 MHz, $CDCl_3$) ***Anti***: δ 147.09, 142.06, 141.09, 133.37, 128.52, 128.34, 128.25, 128.19, 127.56, 126.26, 115.39, 65.45, 52.90, ***Syn***:δ 146.75, 141.92, 139.92, 128.52, 128.10, 127.84, 126.63, 115.73, 62.61, 53.40; HRMS calcd for $C_{17}H_{18}ClO_2$ $[M+H]^+$: 289.0995, found 289.0990.

References

[1] Benkő, Z.; Burck, S.; Gudat, D.; Nieger, M.; Nyulászi, L.; Shore, N. *Dalton Trans.* **2008**, 4937.
[2] Namba, K.; Kishi, Y. *J. Am. Chem. Soc.* **2005**, *127*, 15382.
[3] Darensbourg, D. J.; Mackiewicz, R. M.; Rodgers, J. L.; Fang, C. C.; Billodeaux, D. R.; Reibenspies, J. H. *Inorg. Chem.* **2004**, *43*, 6024.
[4] Kuang, J.; Ma, S. *J. Org. Chem.* **2009**, *74*, 1763.

473_1H NMR (CDCl3, 499.94 MHz)

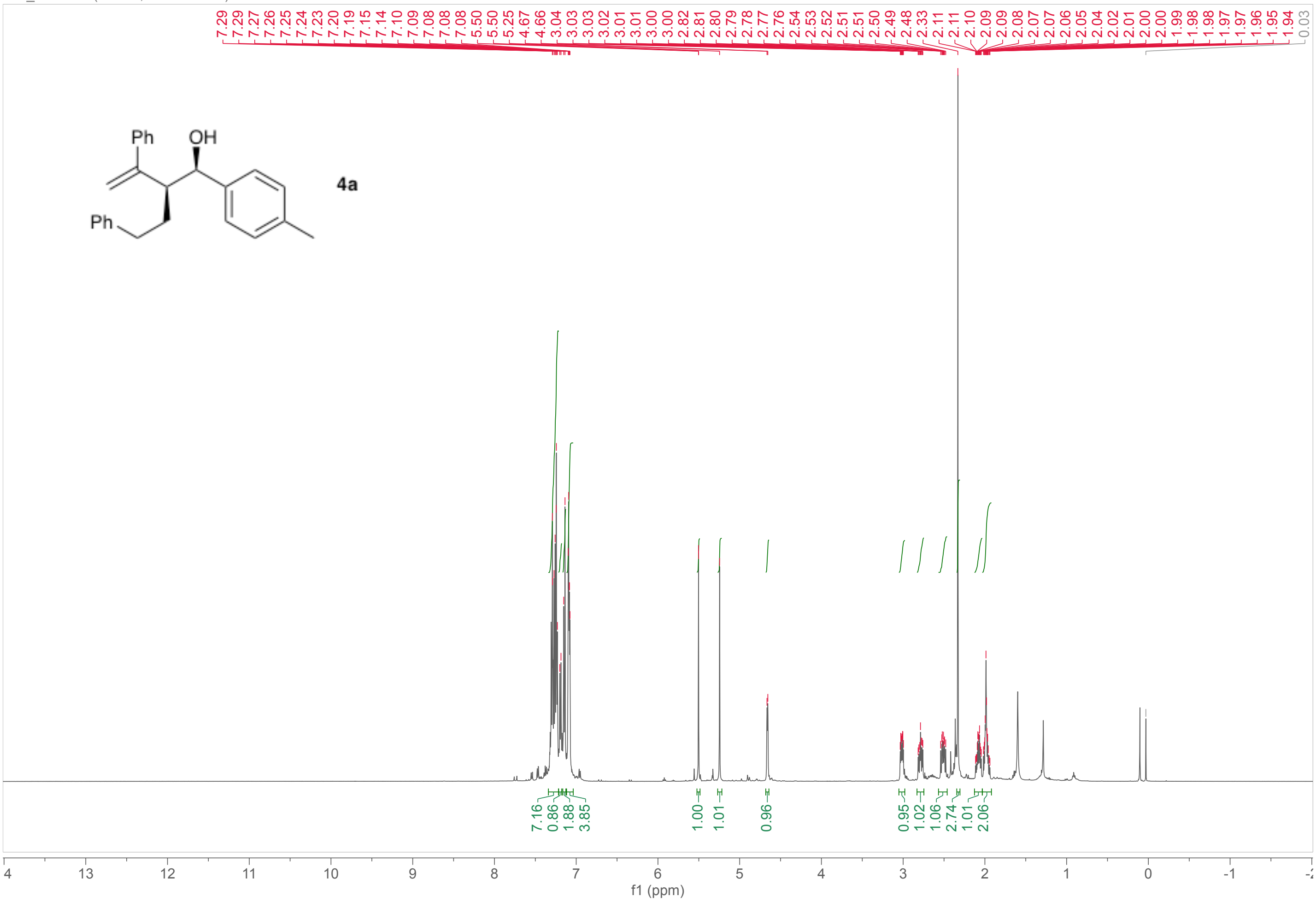

473_13C NMR (CDCl3, Varian-125.72 MHz)

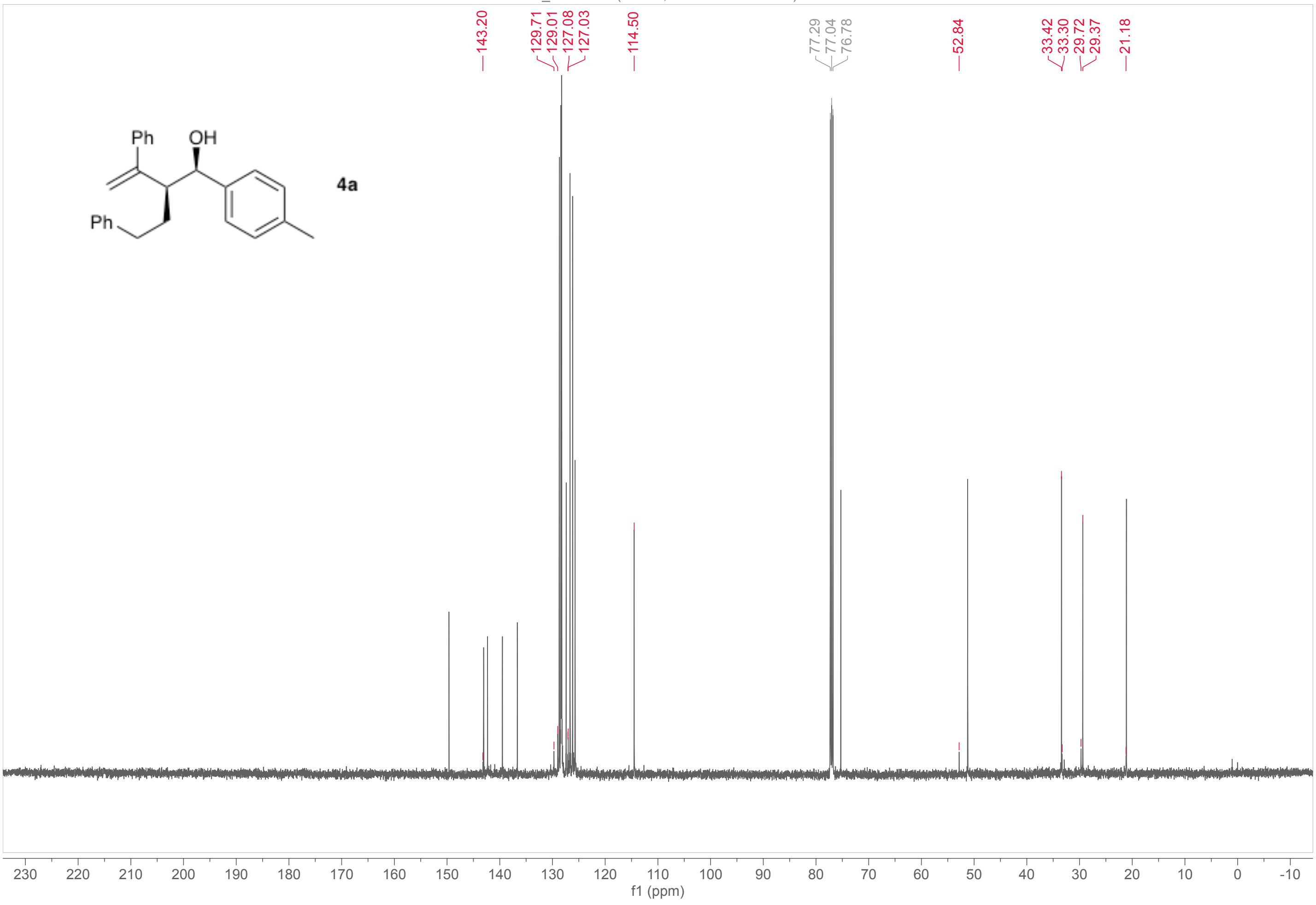

525_1H NMR spectra (499.94 MHz, CDCl3)

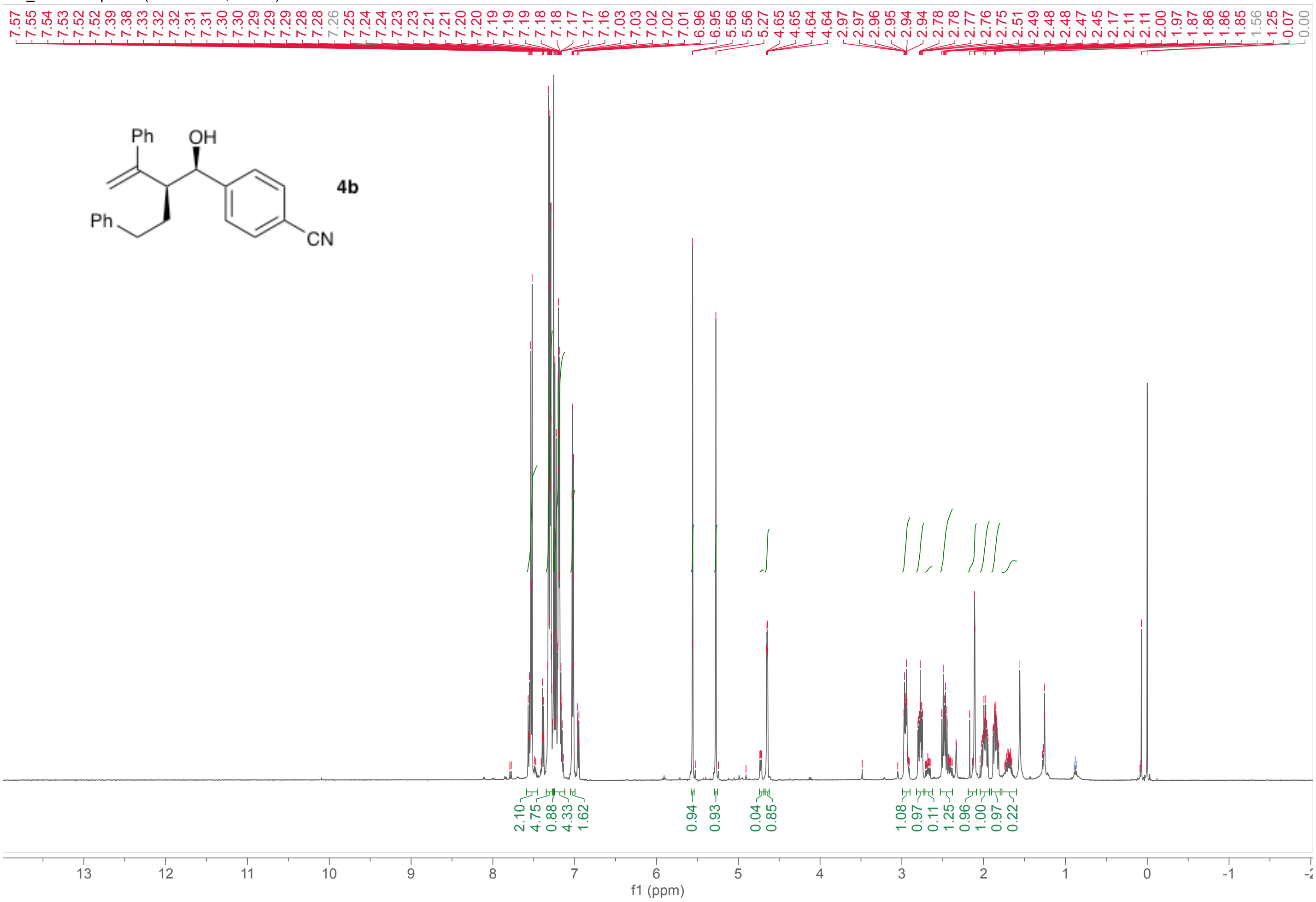

525_13C NMR (CDCl3, Varian-125.72 MHz)

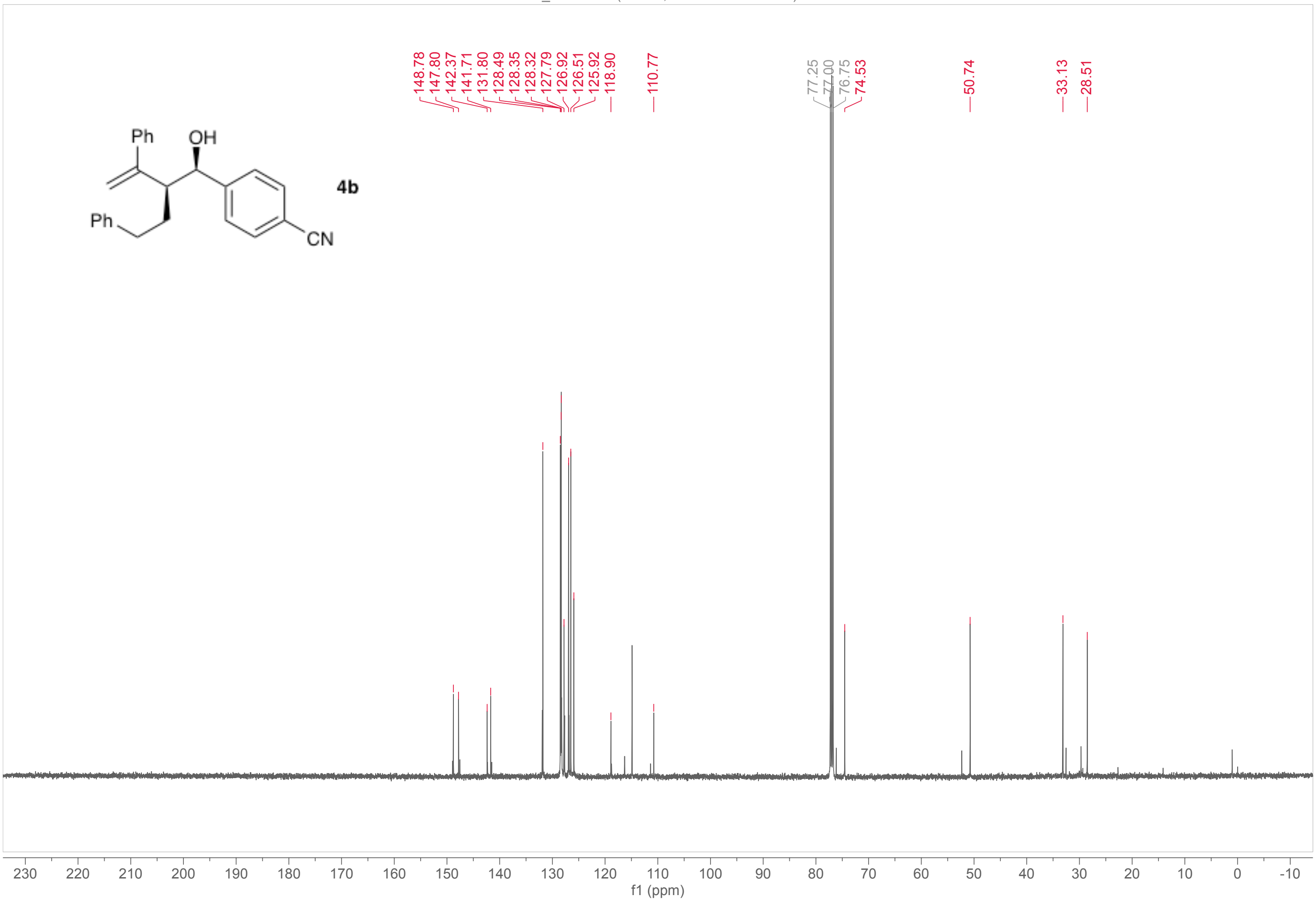

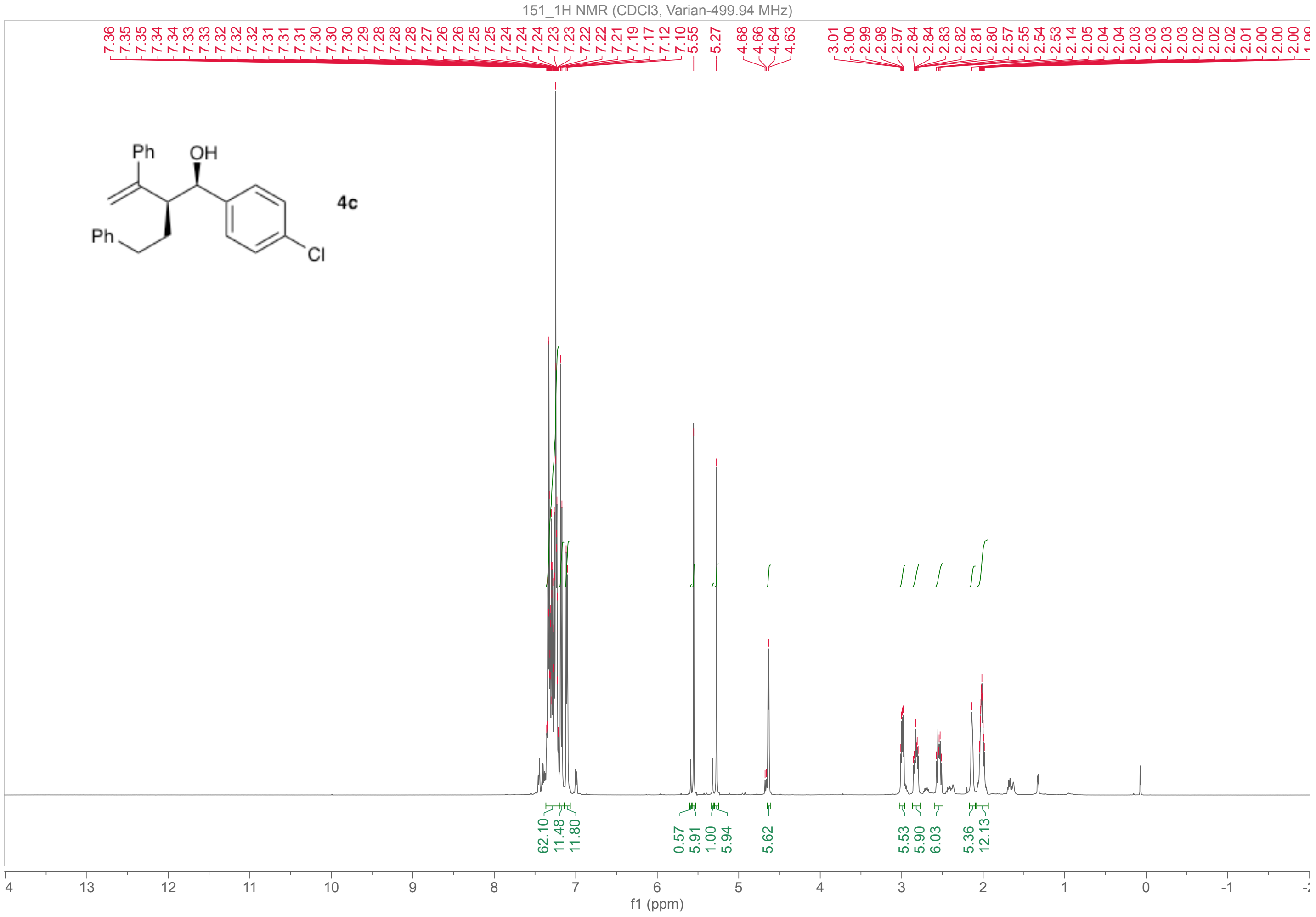
151_1H NMR (CDCl3, Varian-499.94 MHz)
7.36 7.35 7.35 7.34 7.33 7.33 7.32 7.32 7.32 7.31 7.31 7.30 7.30 7.29 7.28 7.28 7.27 7.26 7.26 7.25 7.25 7.24 7.24 7.24 7.23 7.23 7.22 7.22 7.21 7.19 7.17 7.12 7.10 5.55 5.27 4.68 4.66 4.64 4.63 3.01 3.00 2.99 2.98 2.97 2.84 2.84 2.83 2.82 2.81 2.80 2.57 2.55 2.54 2.53 2.14 2.05 2.04 2.04 2.03 2.03 2.03 2.02 2.02 2.01 2.00 2.00
Ph
OH
Ph
Cl
4c
62.10 11.48 11.80 0.57 5.91 1.00 5.94 5.62 5.53 5.90 6.03 5.36 12.13
f1 (ppm)

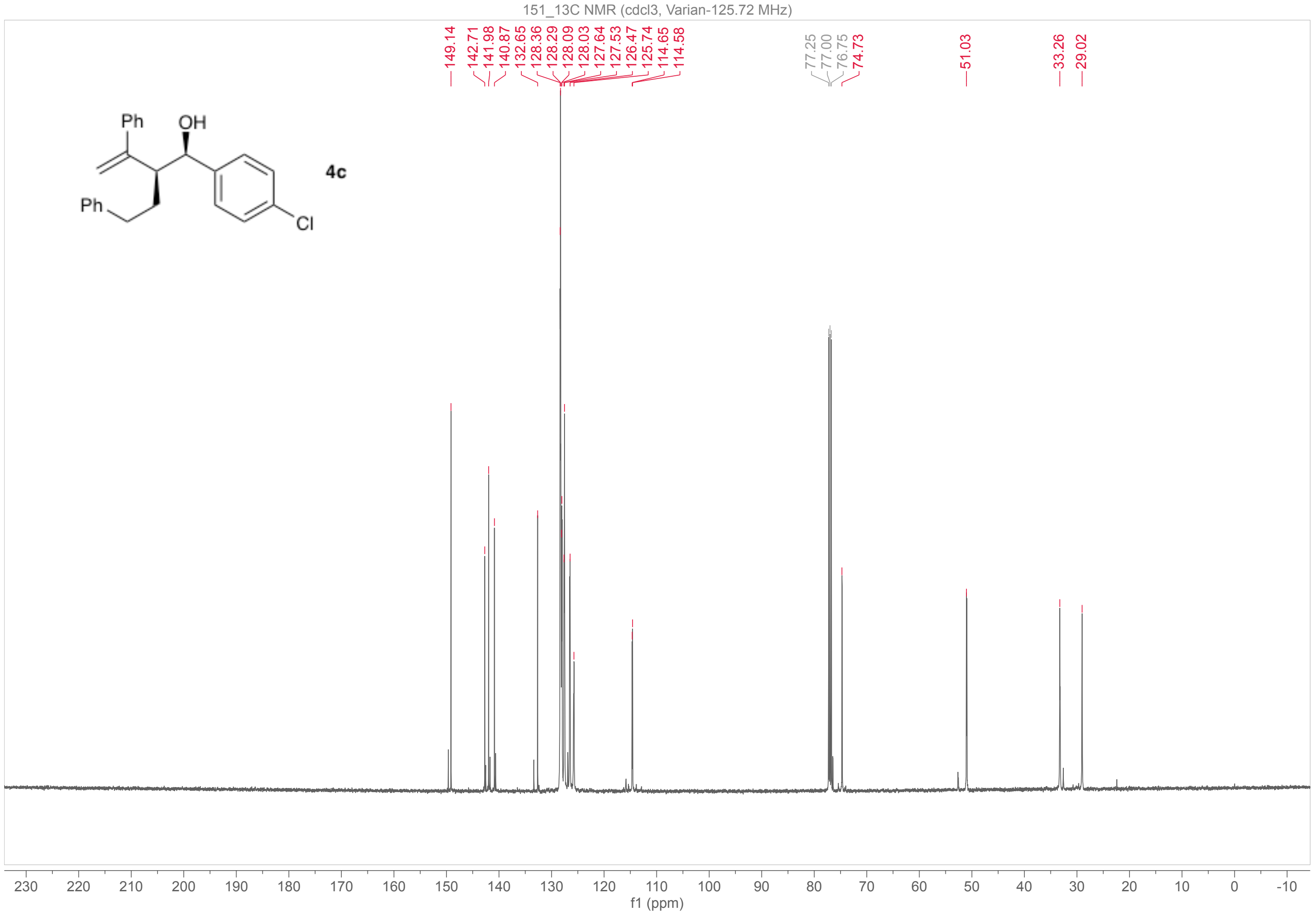
151_13C NMR (cdcl3, Varian-125.72 MHz)
149.14 142.71 141.98 140.87 132.65 128.36 128.29 128.09 128.03 127.64 127.53 126.47 125.74 114.65 114.58
77.25 77.00 76.75 74.73
51.03
33.26 29.02
Ph
OH
Ph
Cl
4c
f1 (ppm)

481_1H NMR (CDCl3, Varian-499.94 MHz)

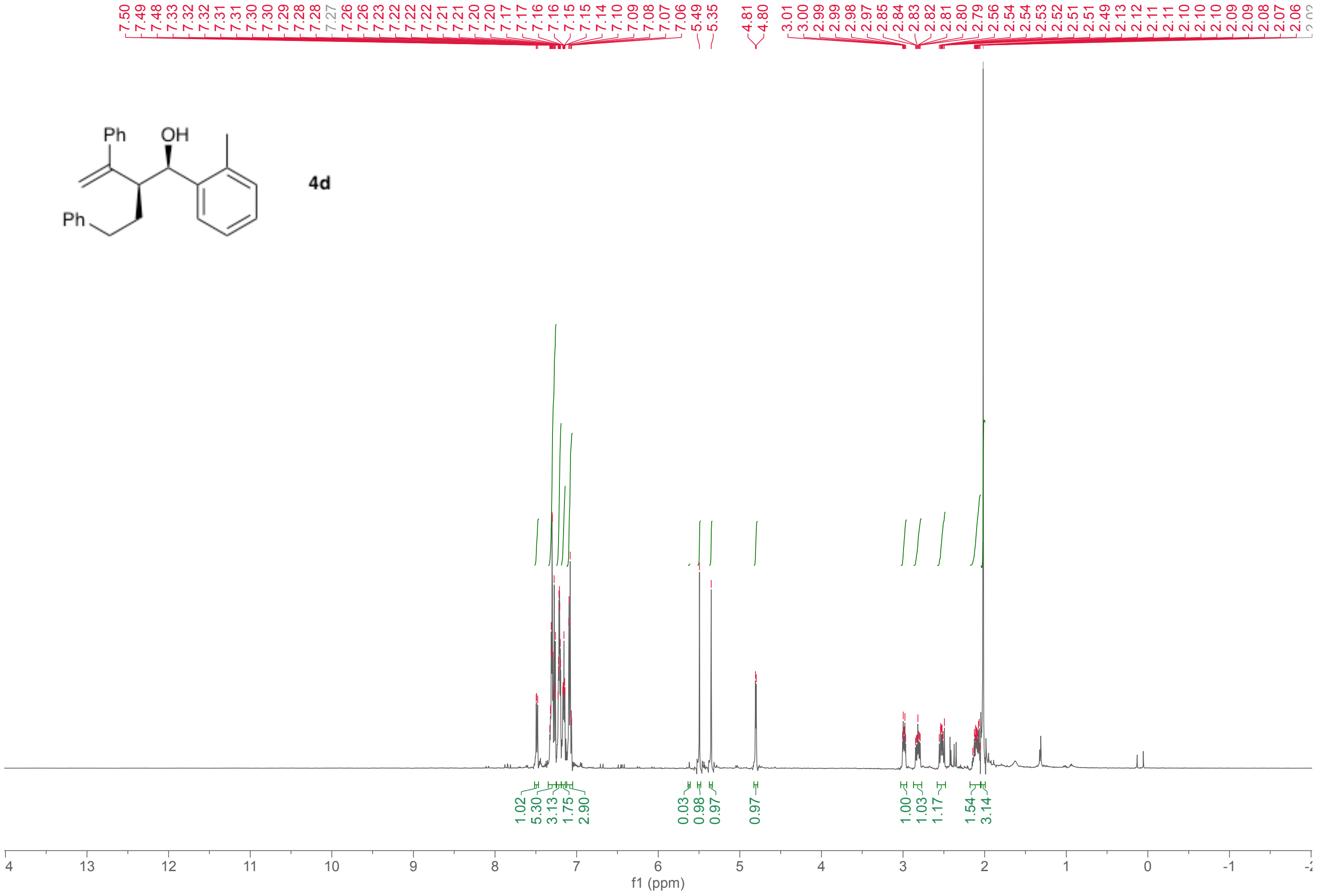

481_13C NMR (CDCl3, Varian-125.72 MHz)

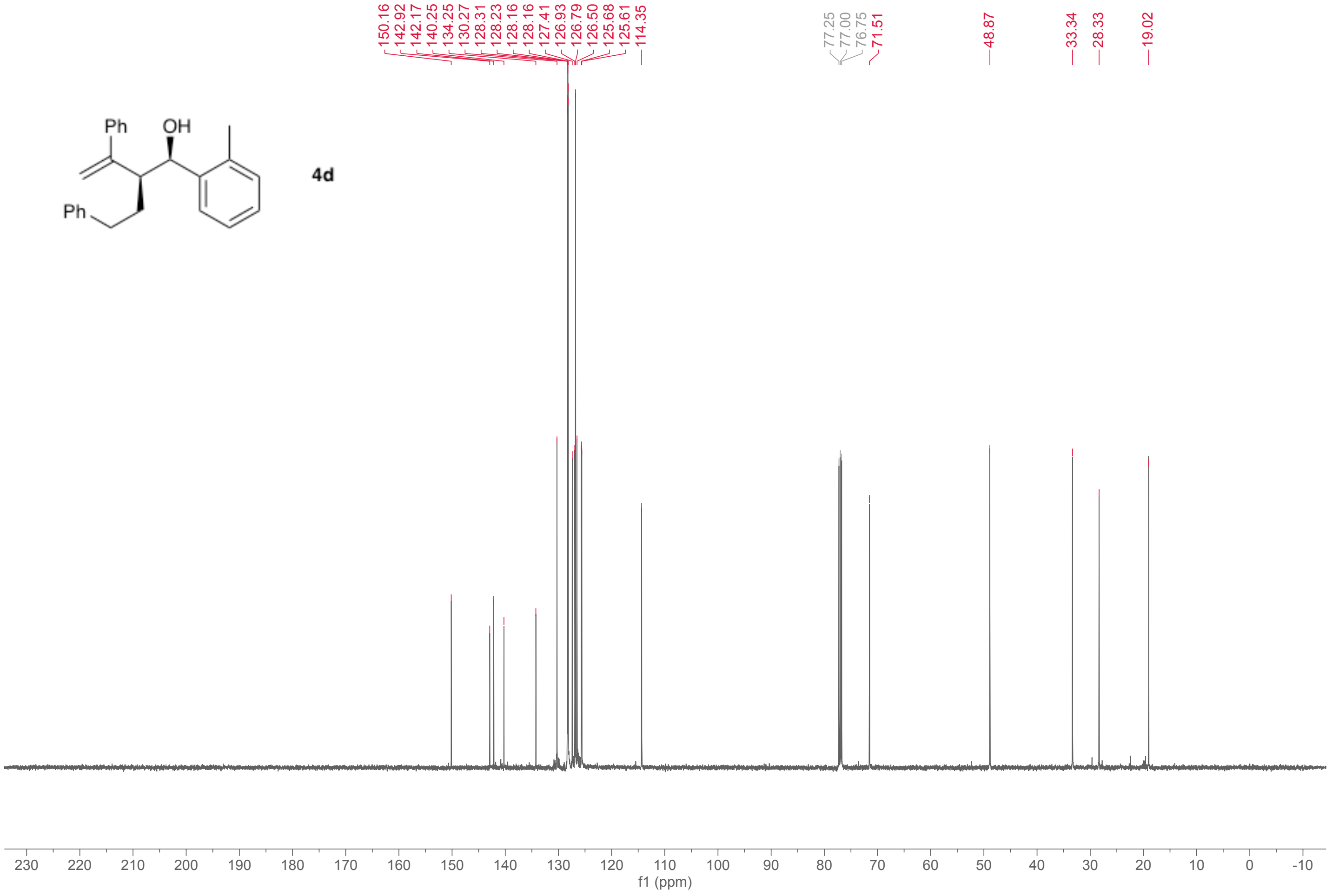

523_1H NMR spectra (499.94 MHz, cdcl3)

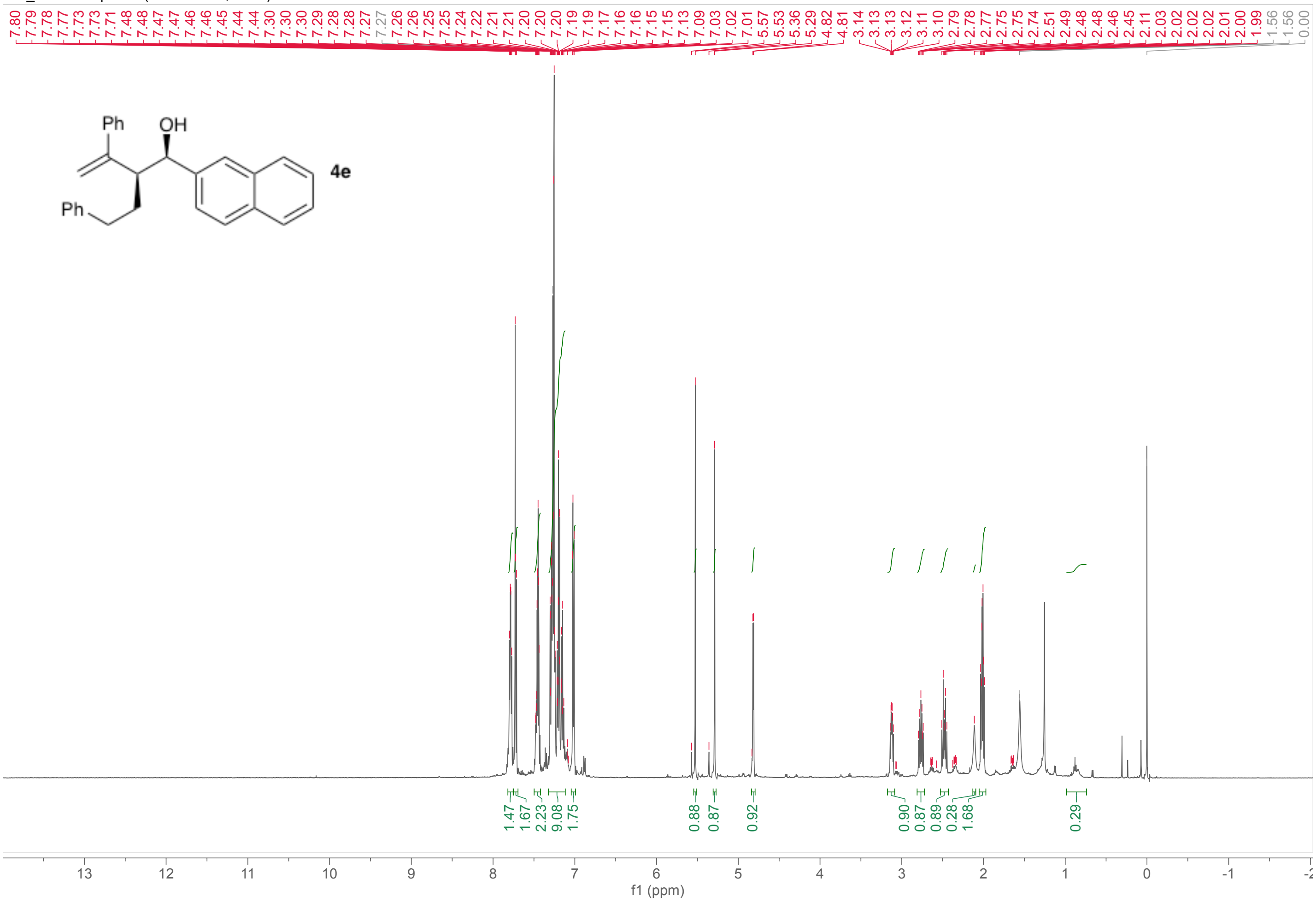

13C NMR spectra of 523 in cdcl3 (125.72 MHz)

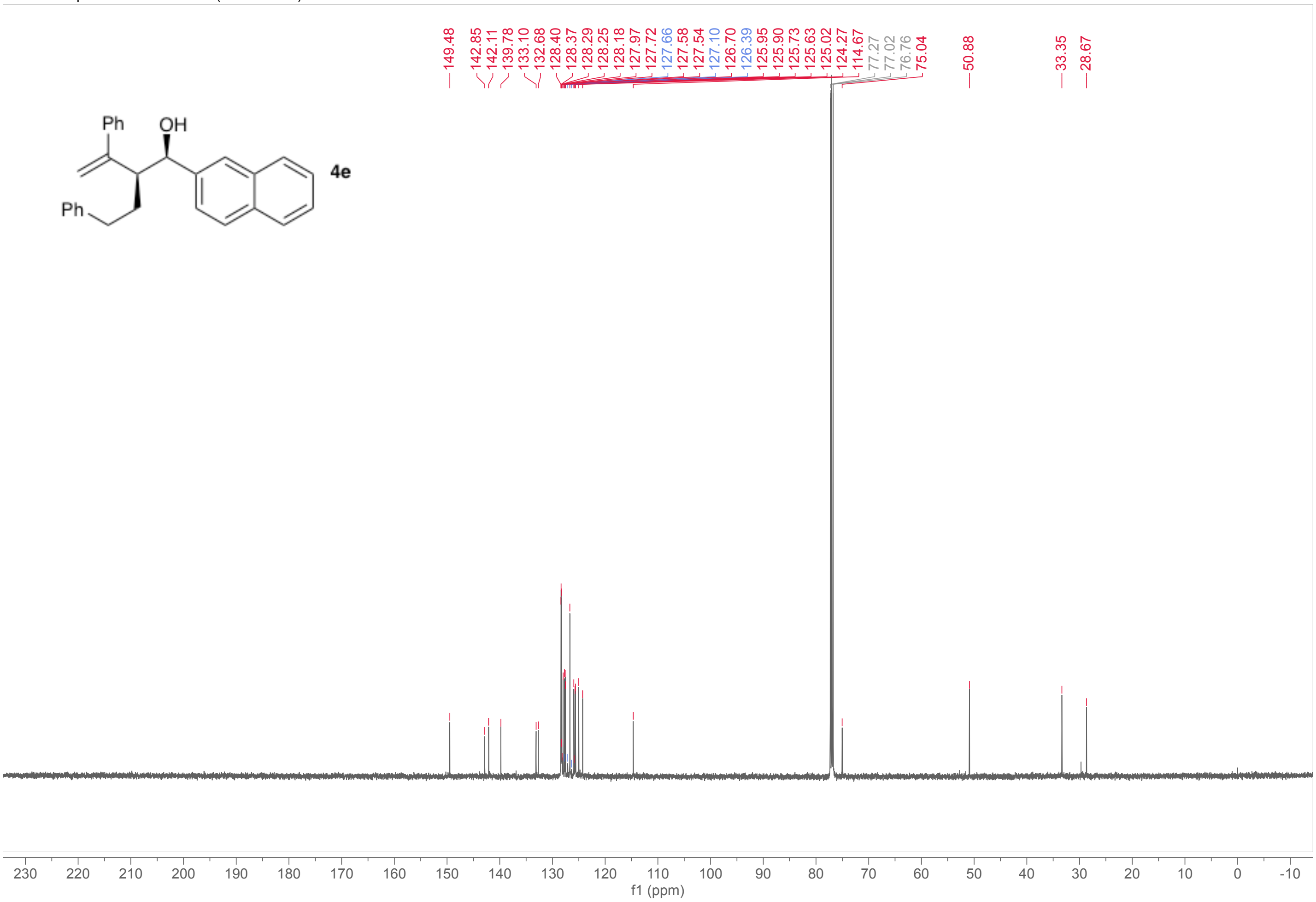

534_1H NMR spectra (499.94 MHz, CDCl3)

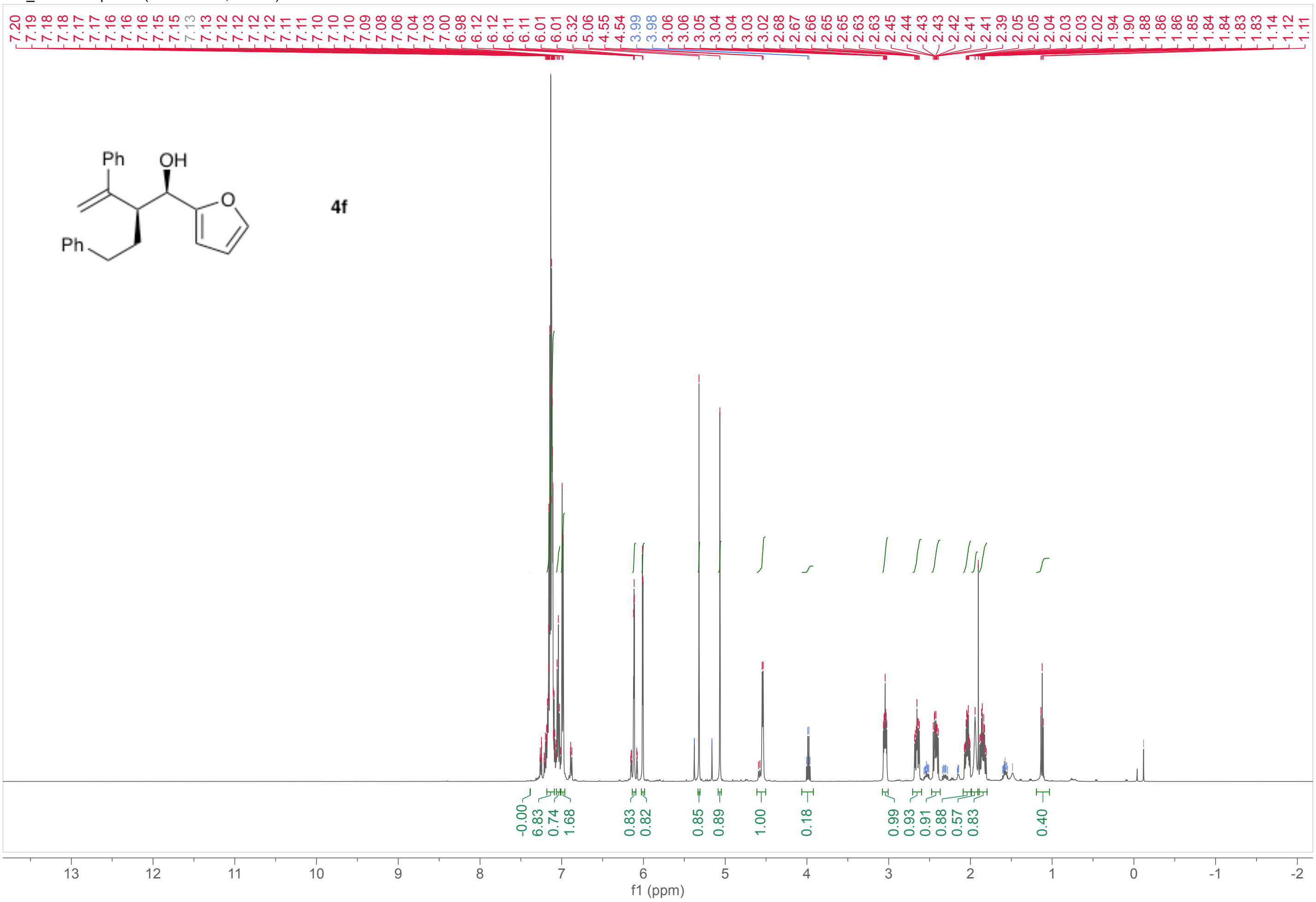

534_13C NMR spectra (125.72 MHz, CDCl3)

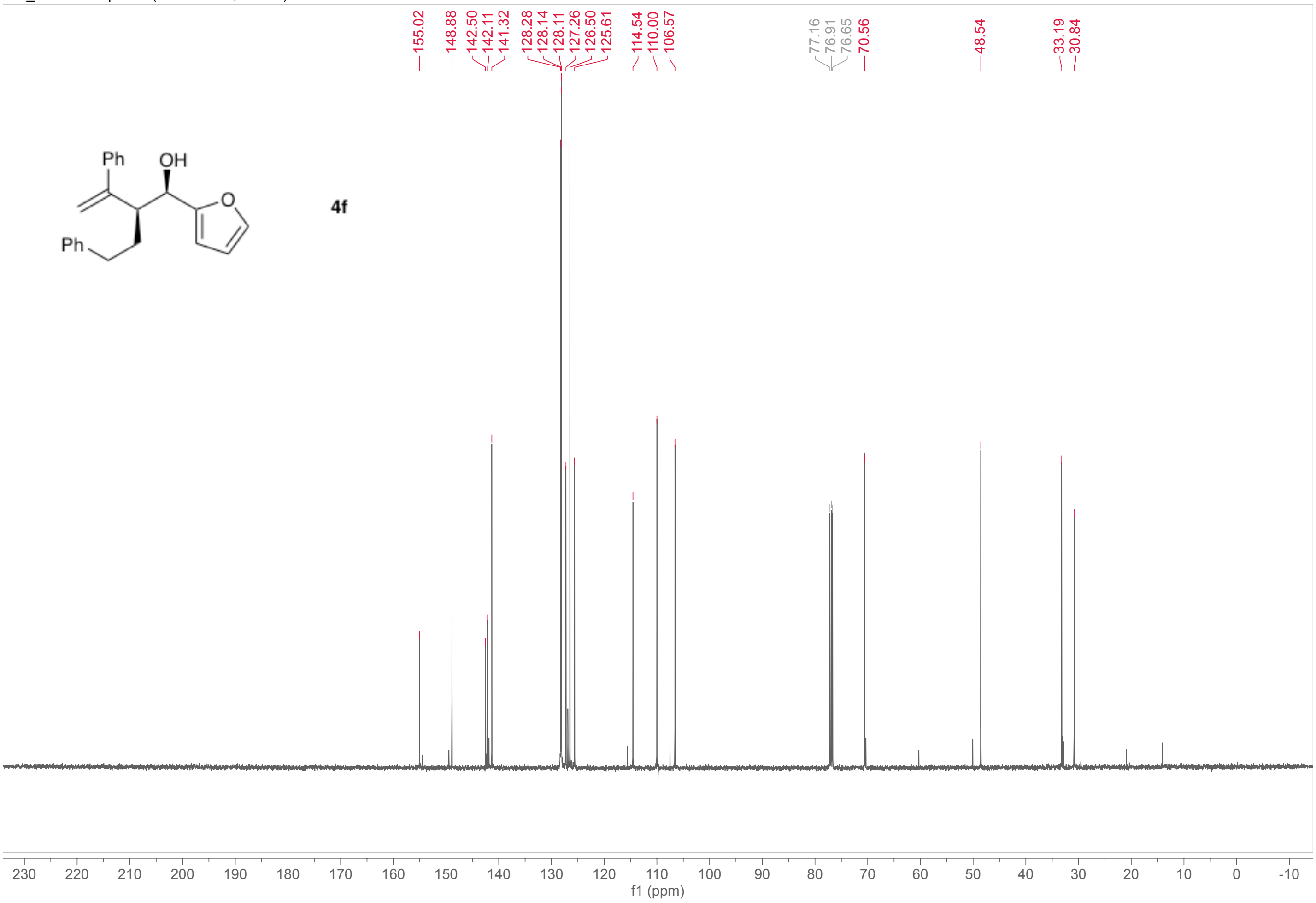

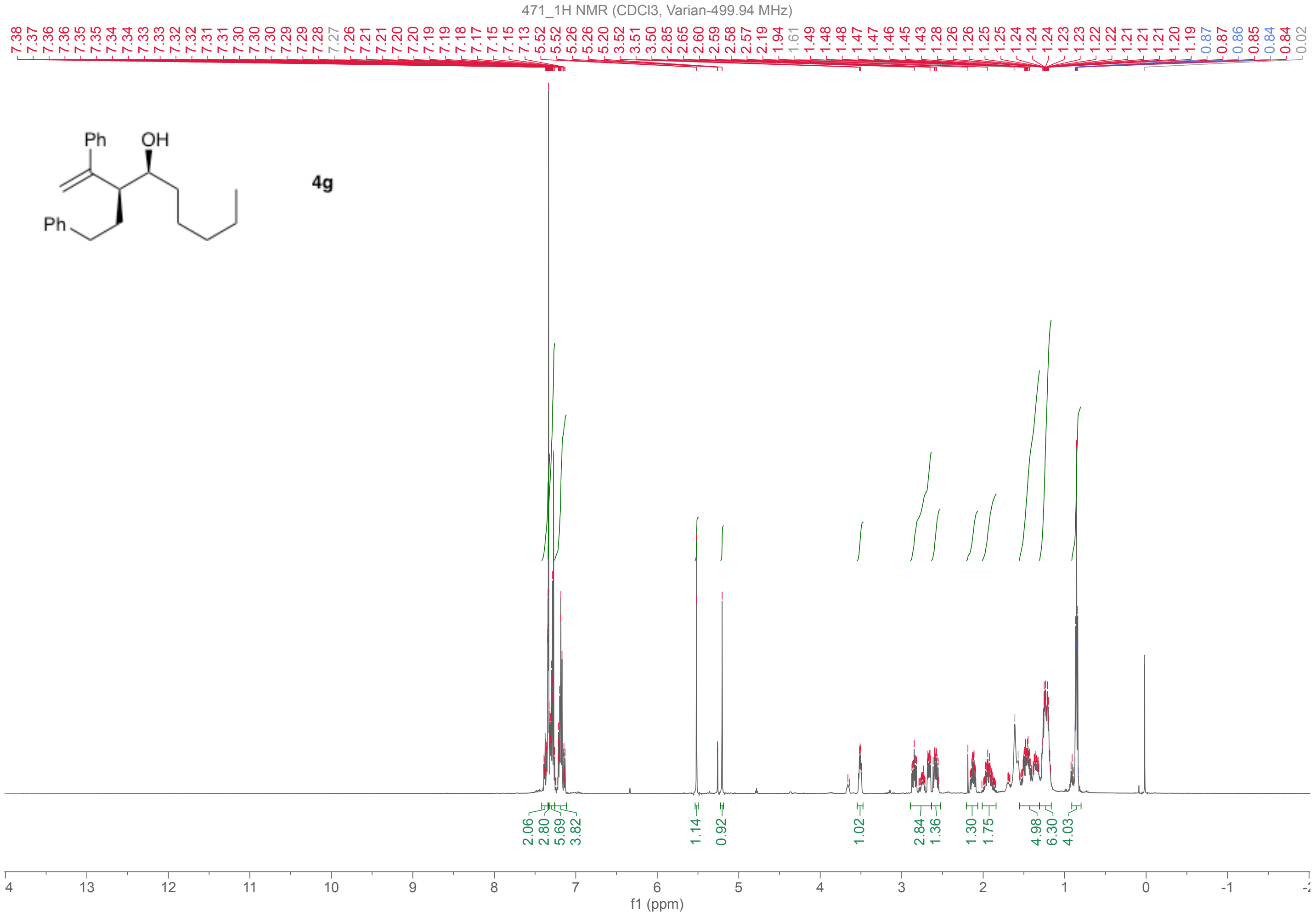
471_1H NMR (CDCl3, Varian-499.94 MHz)
7.38 7.37 7.36 7.35 7.35 7.34 7.34 7.33 7.33 7.32 7.32 7.31 7.31 7.30 7.30 7.30 7.29 7.29 7.28 7.27 7.26 7.21 7.21 7.20 7.20 7.19 7.19 7.18 7.17 7.15 7.15 7.13 5.52 5.52 5.26 5.26 5.20 3.52 3.51 3.50 2.85 2.65 2.60 2.59 2.58 2.57 2.19 1.94 1.61 1.49 1.48 1.48 1.47 1.47 1.46 1.45 1.43 1.28 1.26 1.26 1.25 1.25 1.24 1.24 1.24 1.23 1.23 1.22 1.22 1.21 1.21 1.21 1.20 1.19 0.87 0.87 0.86 0.85 0.84 0.84 0.02
Ph
OH
Ph
4g
2.06 2.80 5.69 3.82 1.14 0.92 1.02 2.84 1.36 1.30 1.75 4.98 6.30 4.03
4 13 12 11 10 9 8 7 6 5 4 3 2 1 0 -1
f1 (ppm)

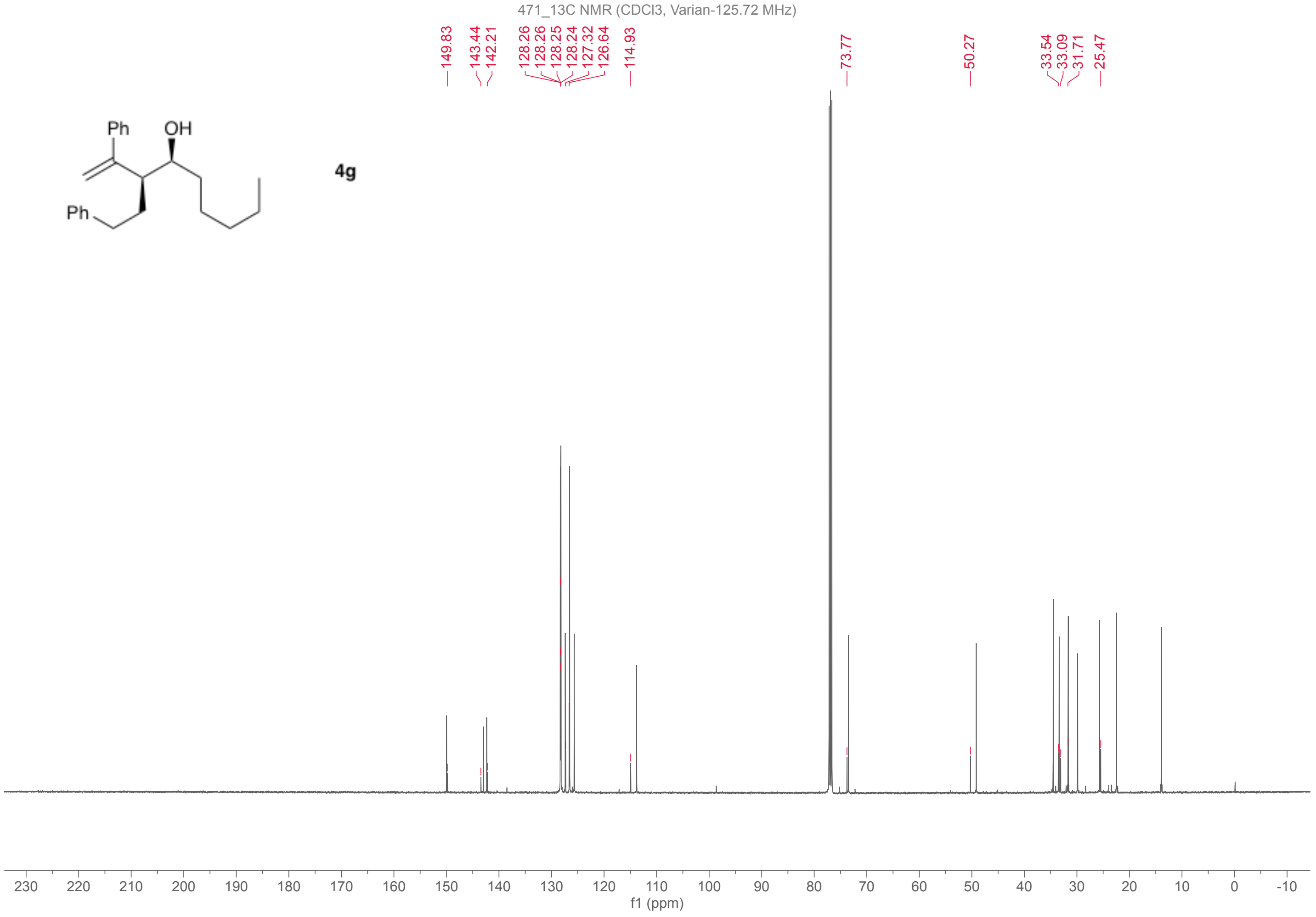
471_13C NMR (CDCl3, Varian-125.72 MHz)
149.83 143.44 142.21 128.26 128.26 128.25 128.24 127.32 126.64 114.93 73.77 50.27 33.54 33.09 31.71 25.47
Ph
OH
Ph
4g
230 220 210 200 190 180 170 160 150 140 130 120 110 100 90 80 70 60 50 40 30 20 10 0 -10
f1 (ppm)

147_1H NMR (CDCl3, Varian-499.94 MHz)

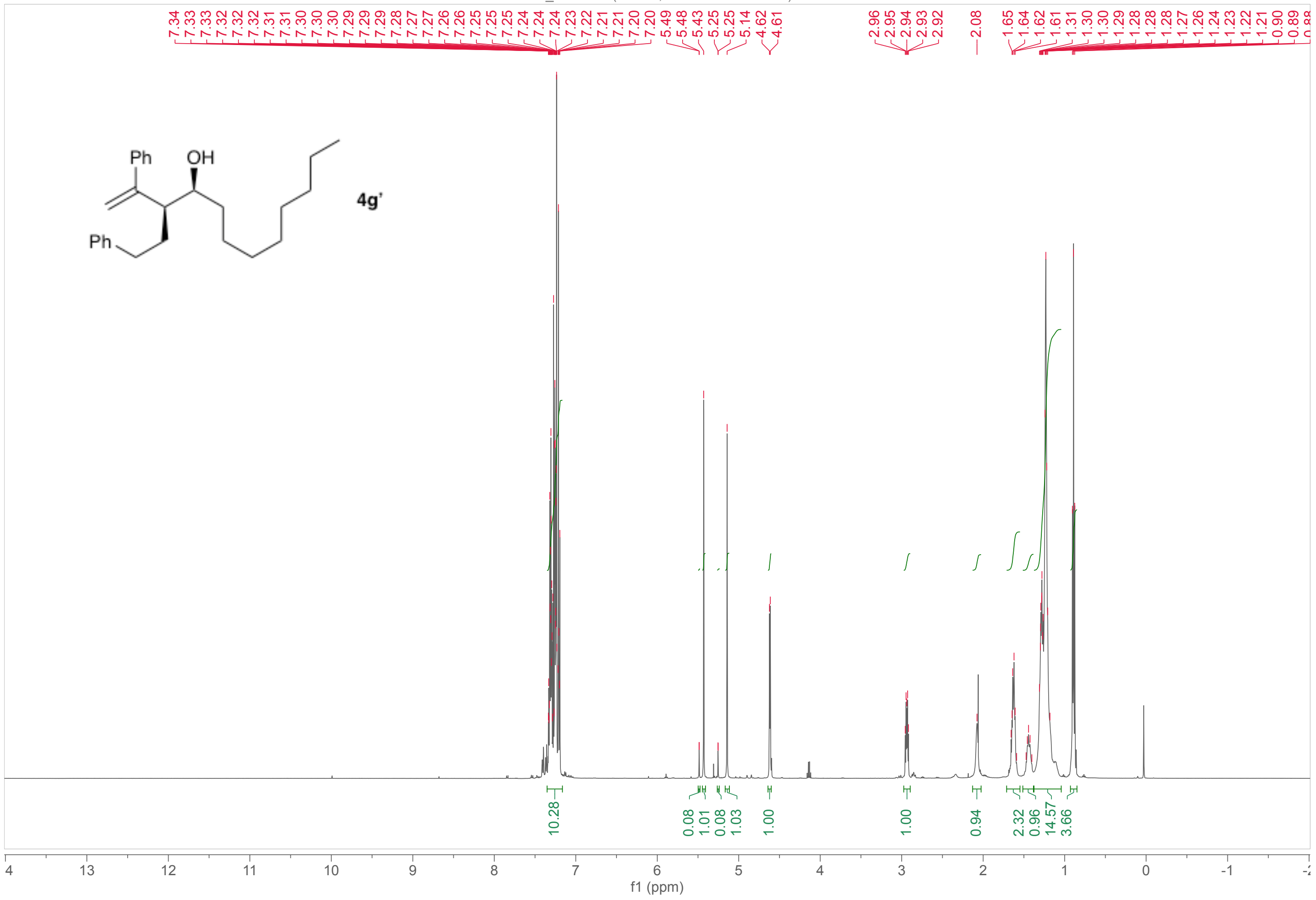

147_13C NMR (CDCl3, Varian-100.57 MHz)

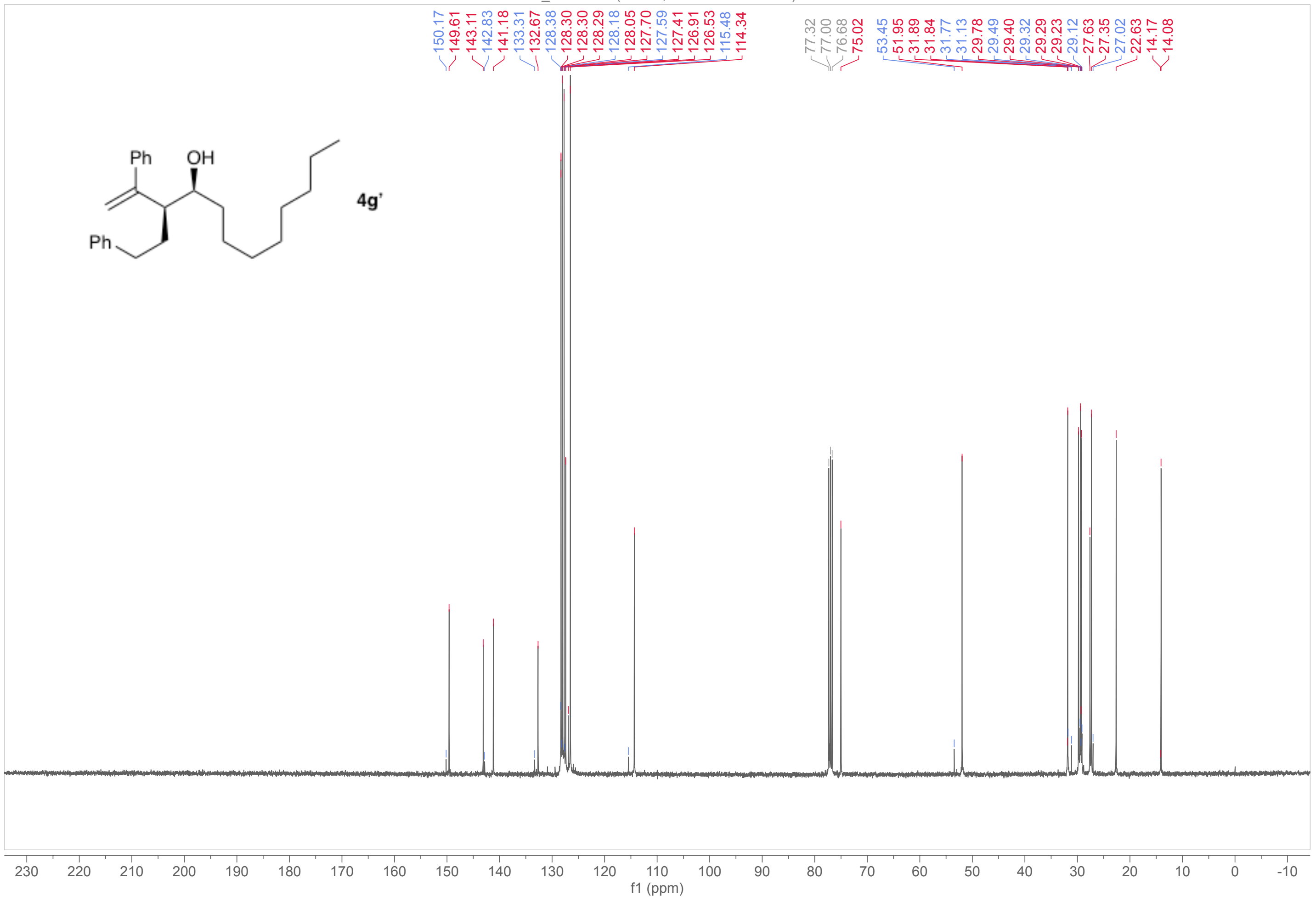

142_1H NMR (CDCl3, Varian-399.90 MHz)

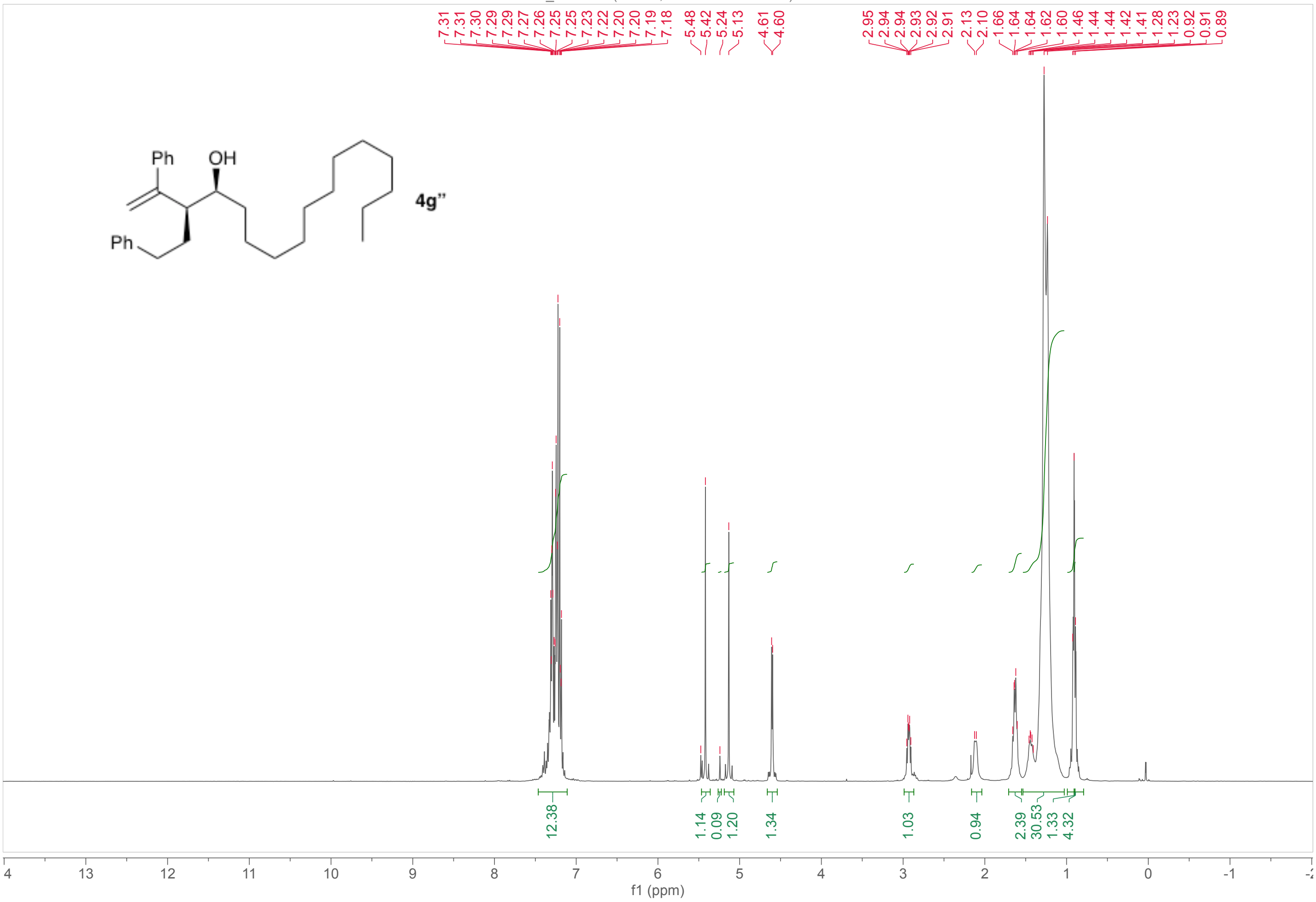

142_13C NMR (CDCl3, Varian-100.57 MHz)

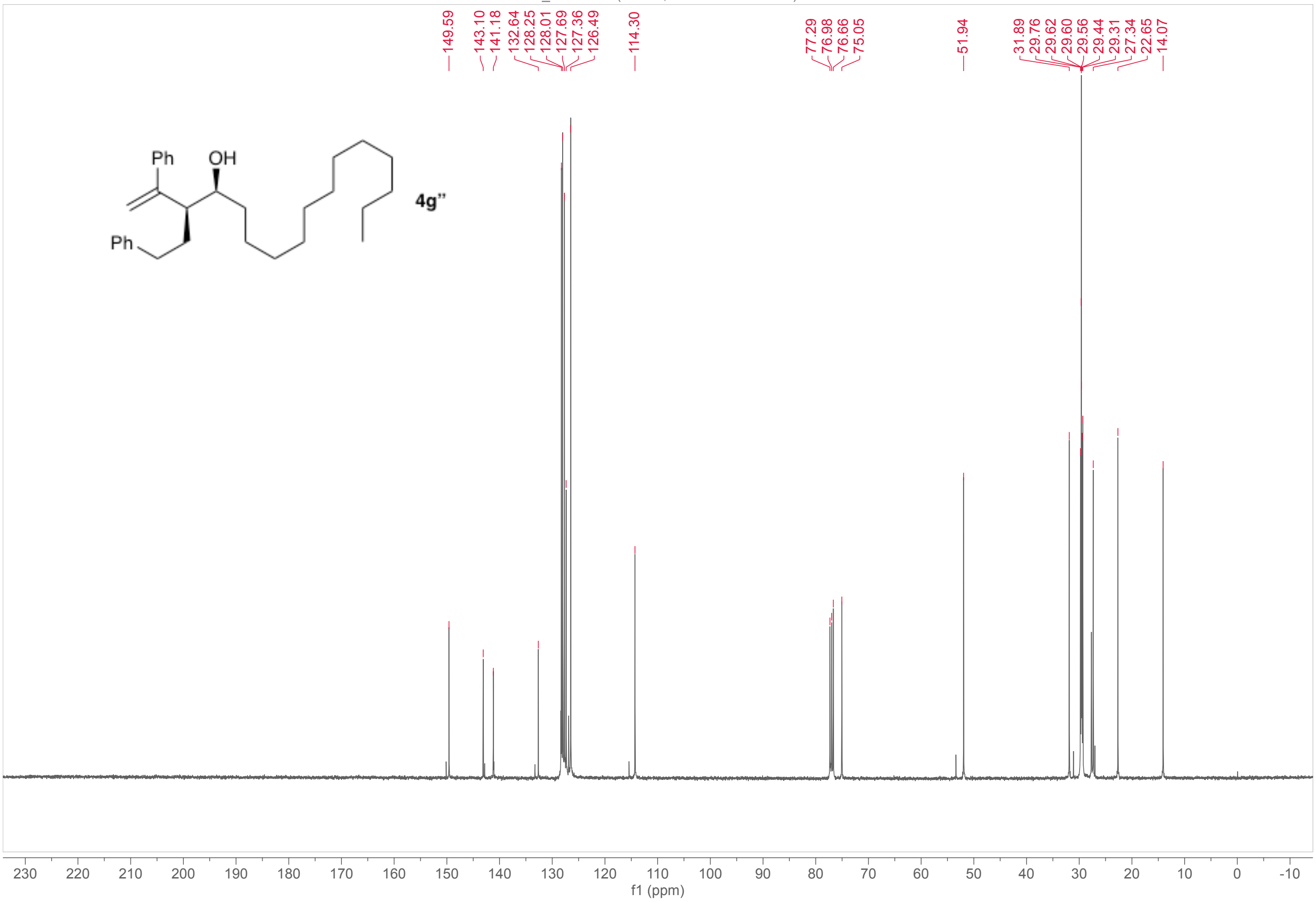

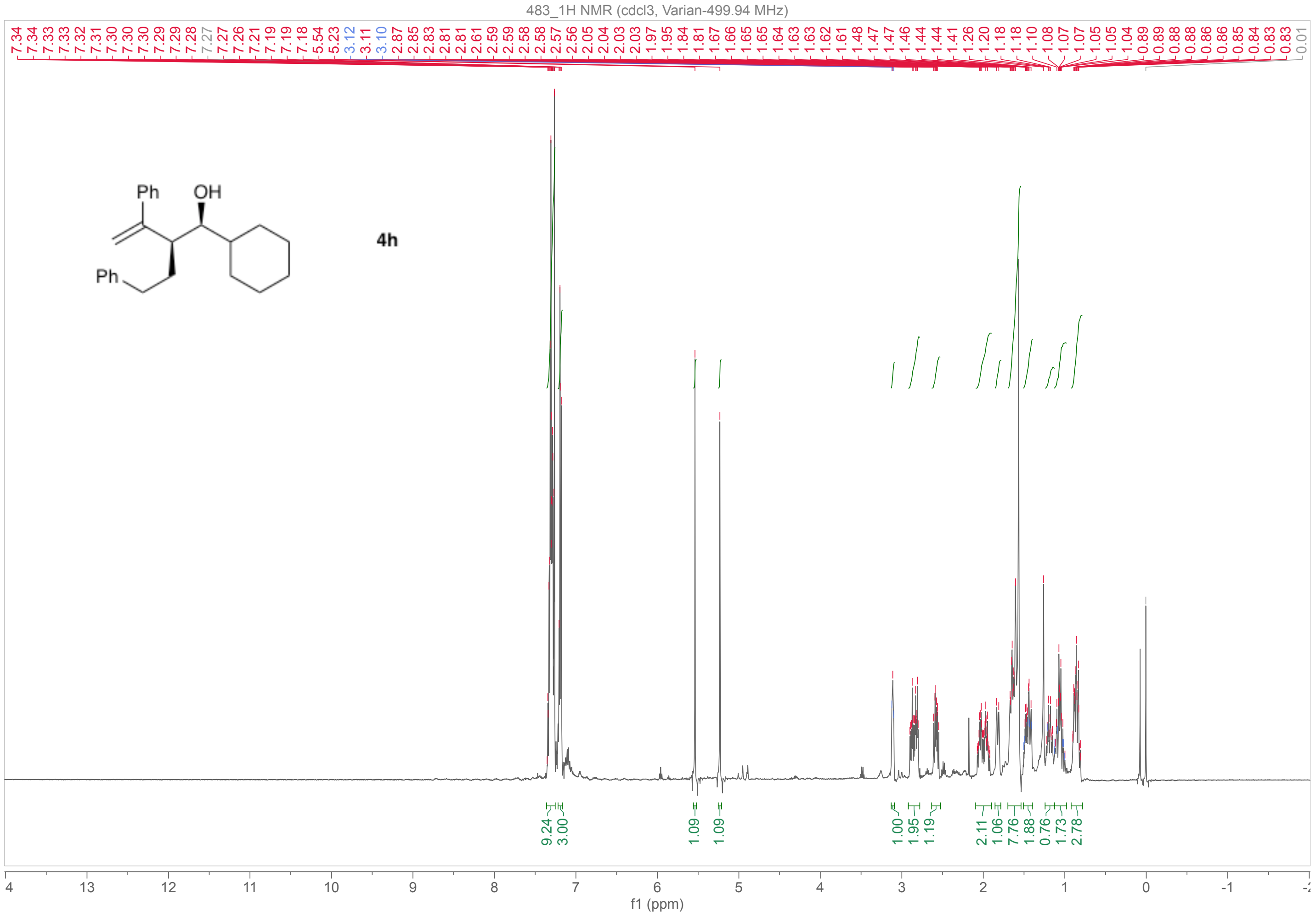
483_1H NMR (cdcl3, Varian-499.94 MHz)
Ph
OH
Ph
4h
f1 (ppm)

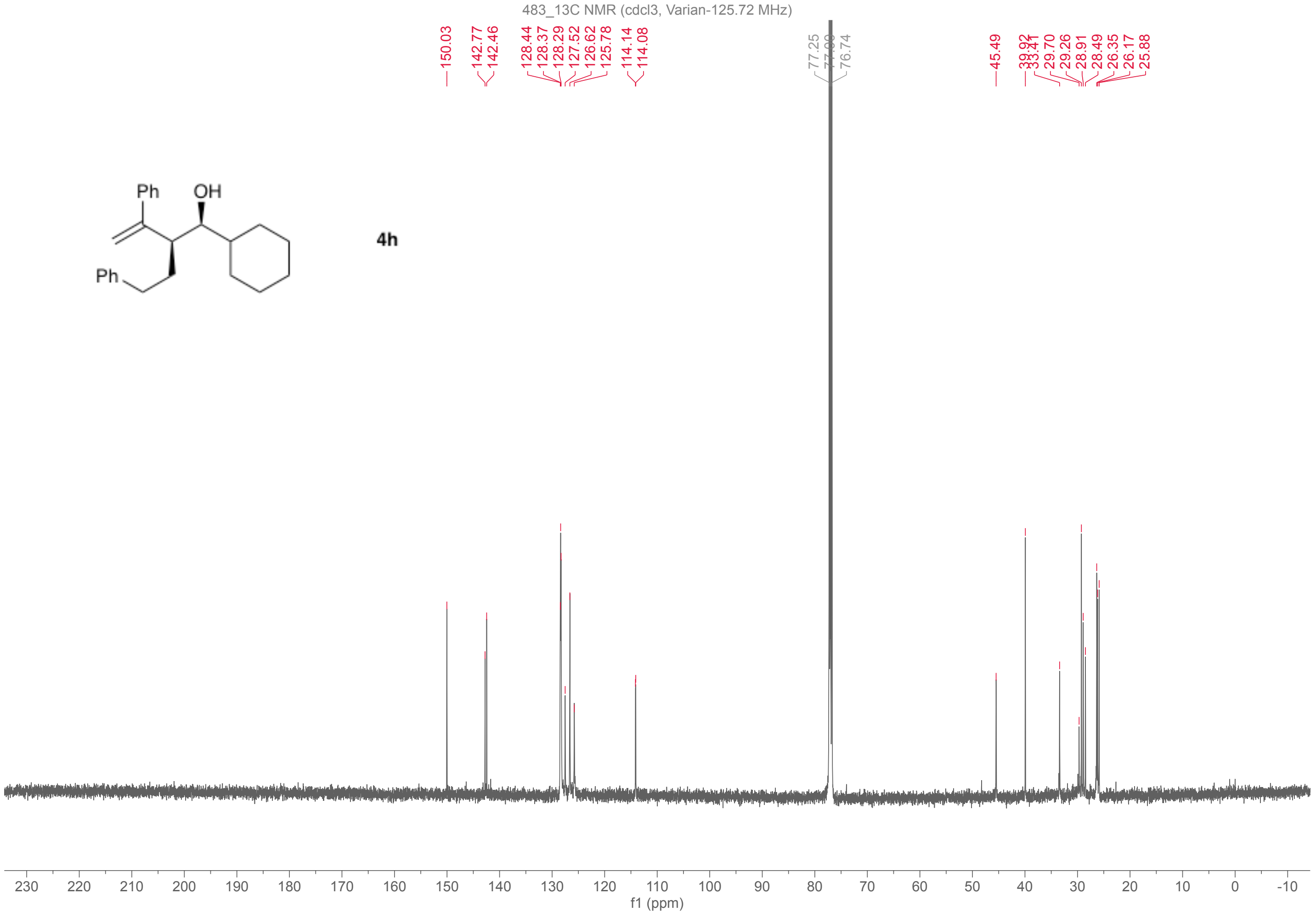
483_13C NMR (cdcl3, Varian-125.72 MHz)
150.03
142.77
142.46
128.44
128.37
128.29
127.52
126.62
125.78
114.14
114.08
77.25
76.74
45.49
33.41
29.70
29.26
28.91
28.49
26.35
26.17
25.88
Ph
OH
Ph
4h
f1 (ppm)

479_1H NMR (CDCl3, 499.94 MHz)

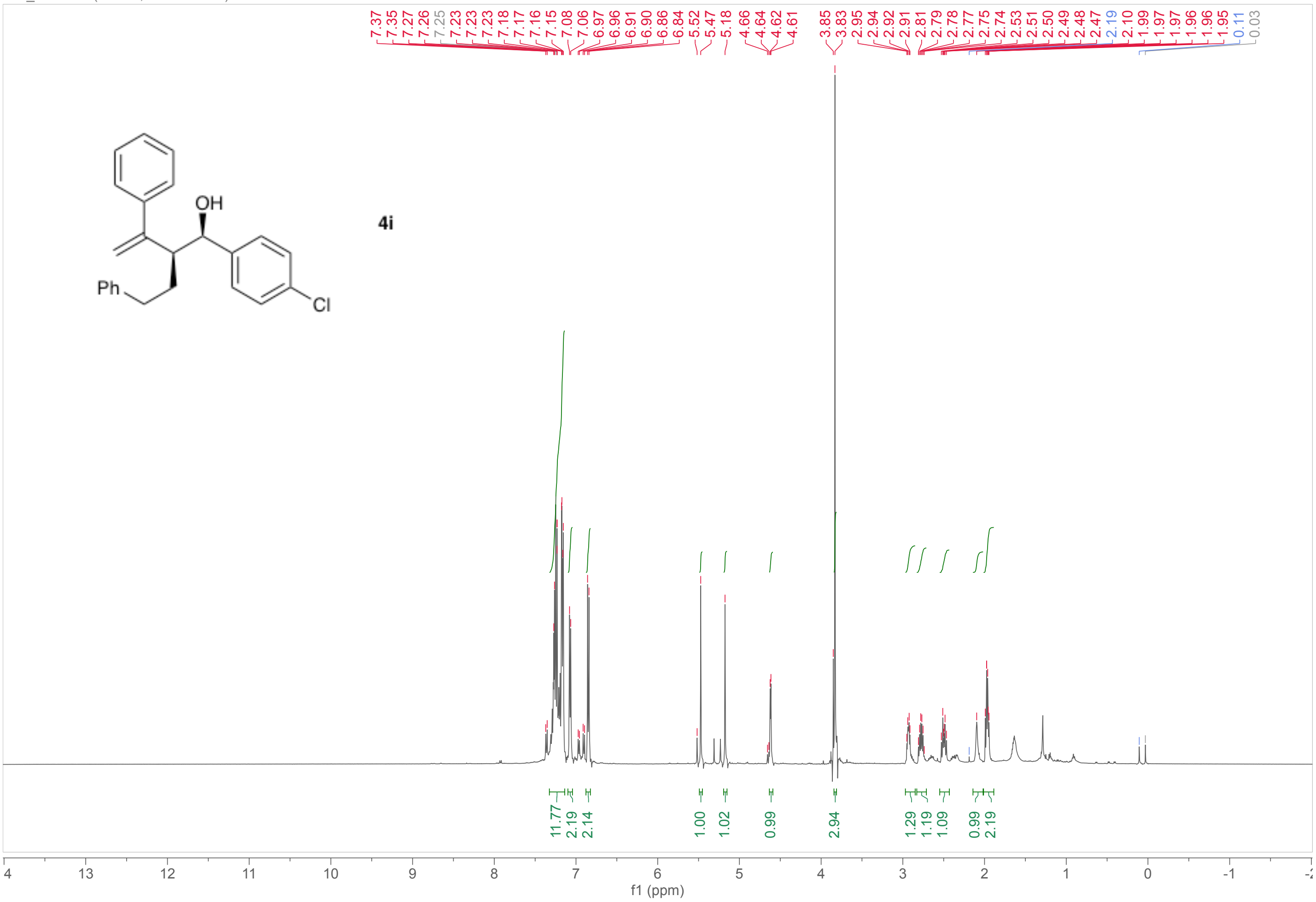

479_13C NMR (CDCl3, Varian-125.72 MHz)

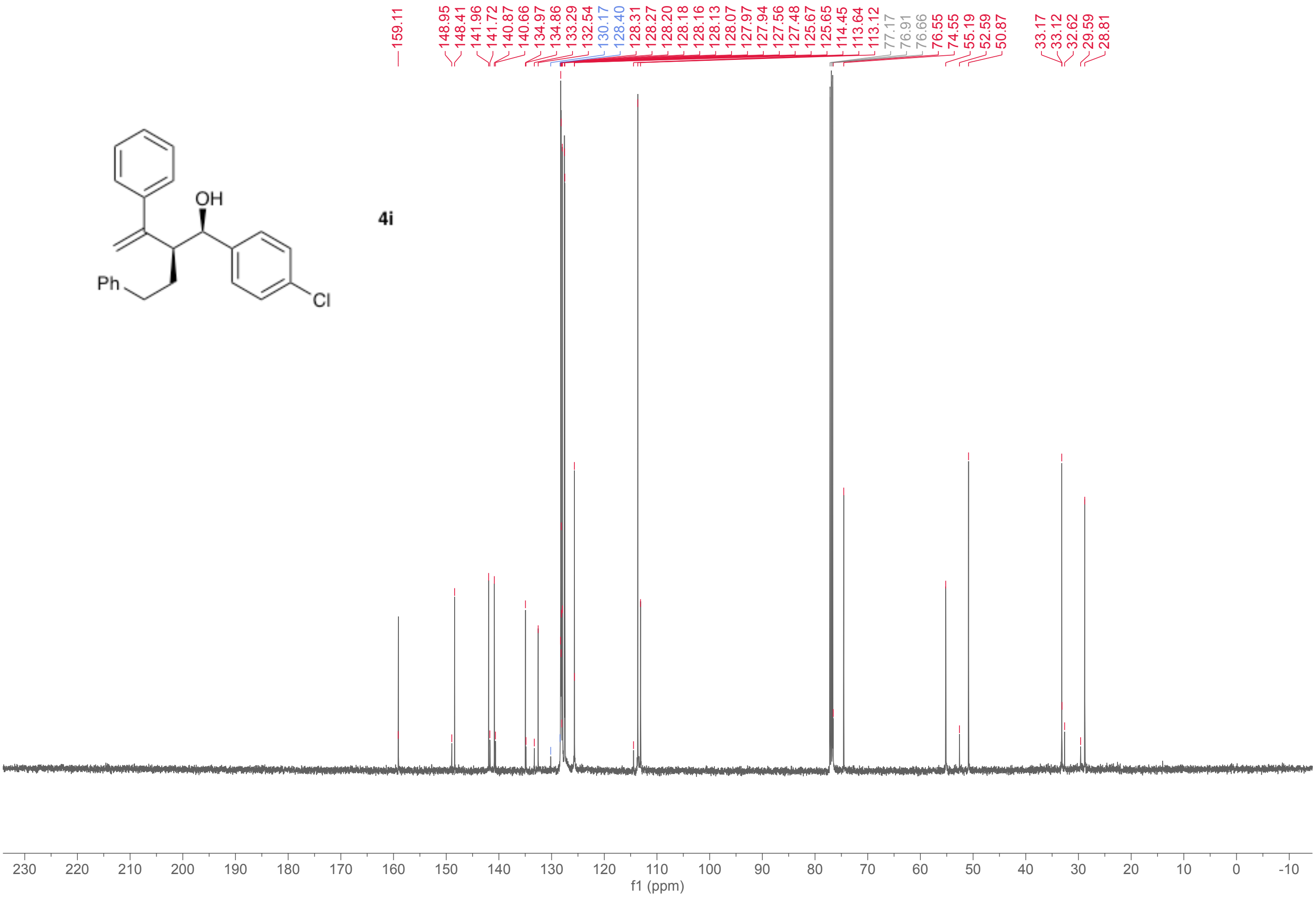

479_1H NMR (cdcl3, Varian-499.94 MHz)

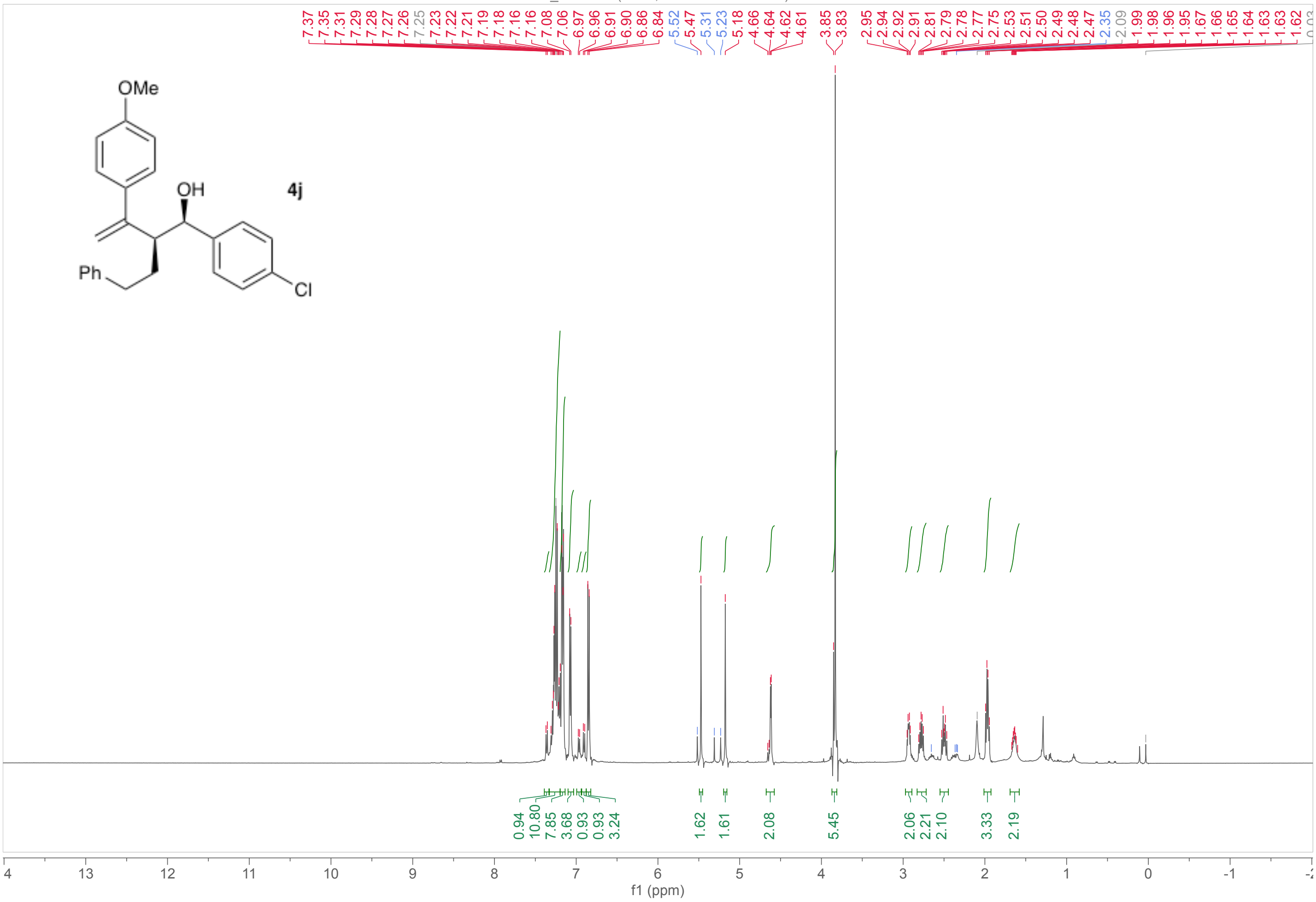

479_13C NMR (cdcl3, Varian-125.72 MHz)

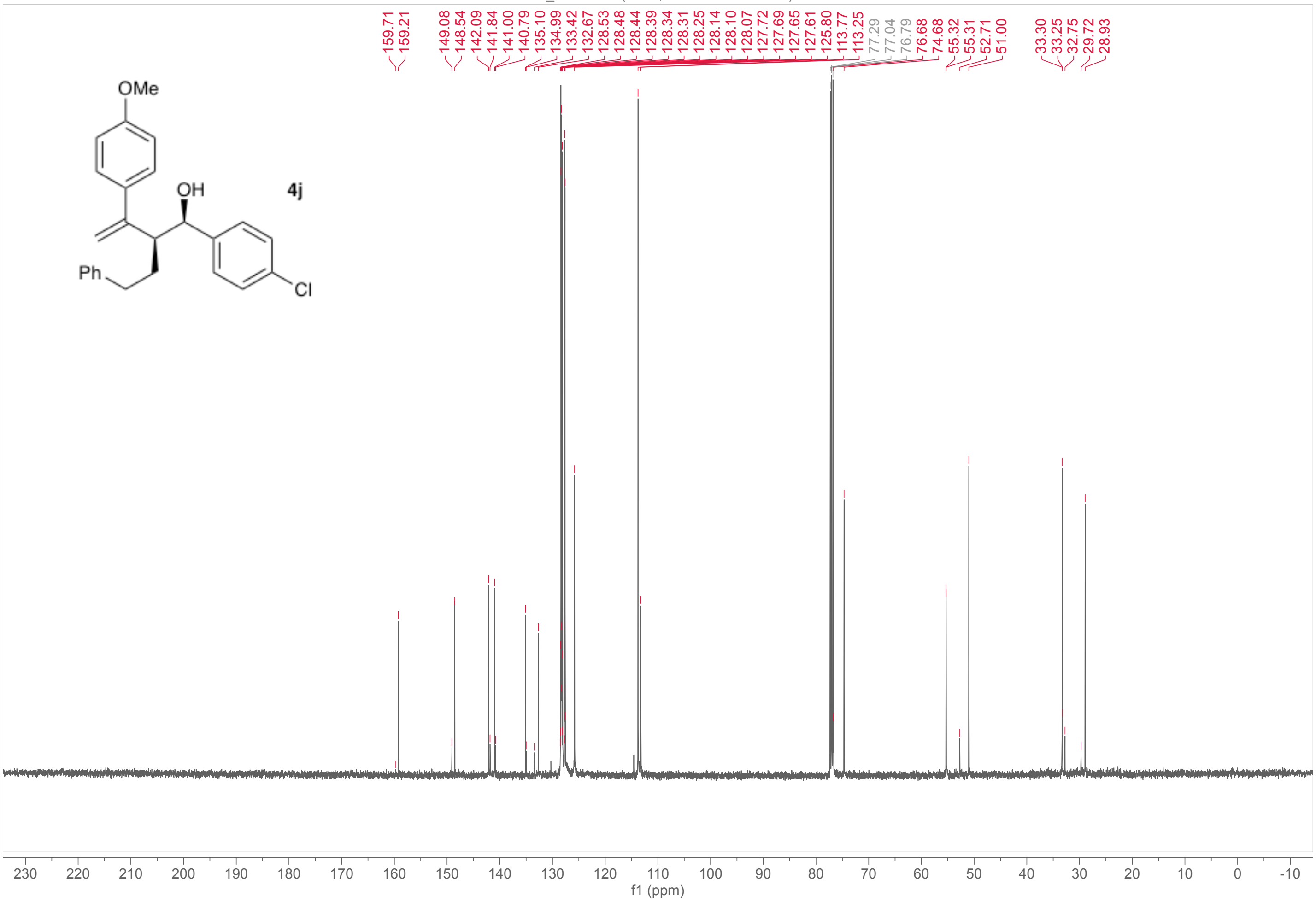

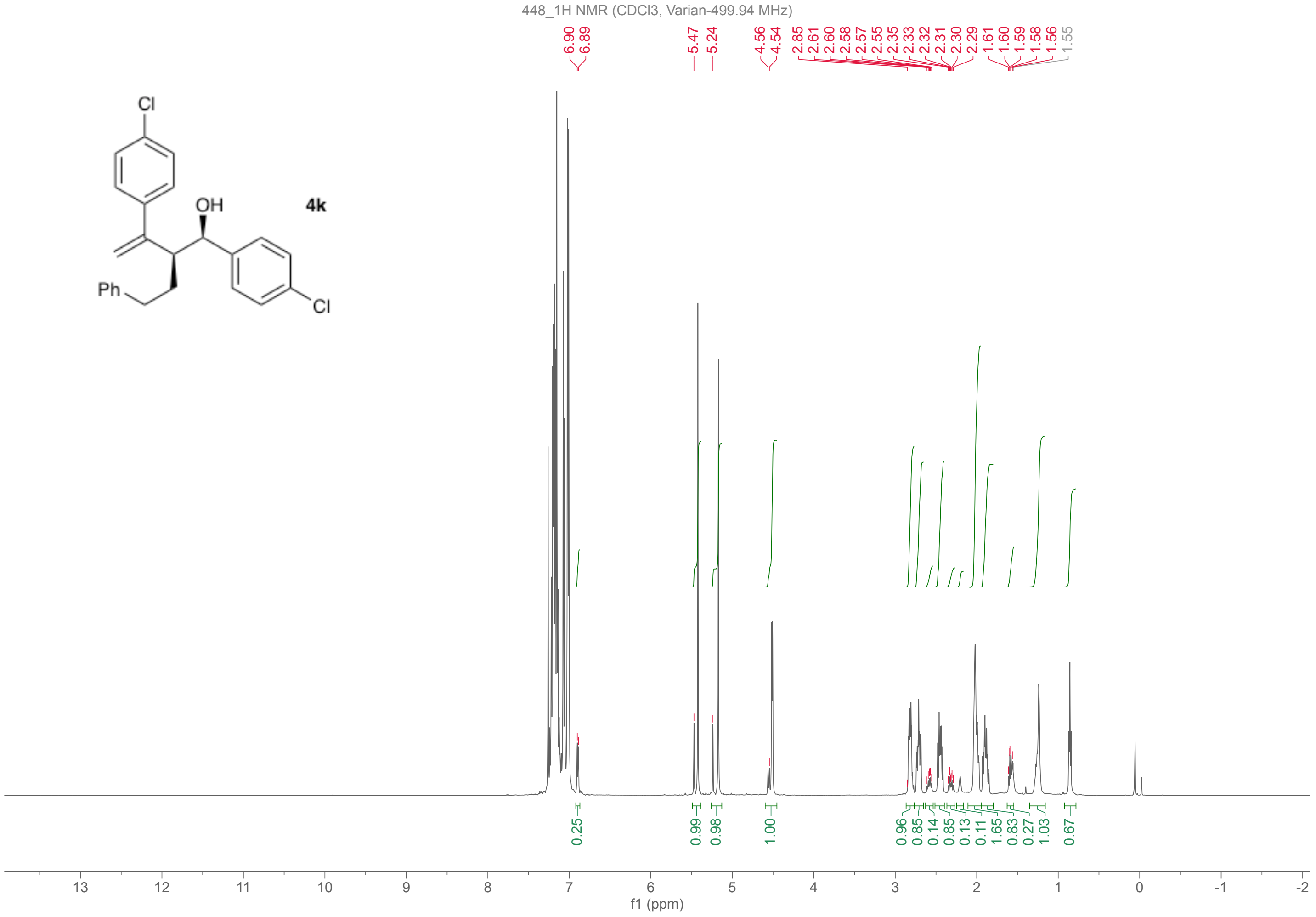
448_1H NMR (CDCl3, Varian-499.94 MHz)
6.90
6.89
5.47
5.24
4.56
4.54
2.85
2.61
2.60
2.58
2.57
2.55
2.35
2.33
2.32
2.31
2.30
2.29
1.61
1.60
1.59
1.58
1.56
1.55
Cl
OH
4k
Ph
Cl
0.25
0.99
0.98
1.00
0.96
0.85
0.14
0.85
0.13
0.11
1.65
0.83
0.27
1.03
0.67
13
12
11
10
9
8
7
6
5
4
3
2
1
0
-1
-2
f1 (ppm)

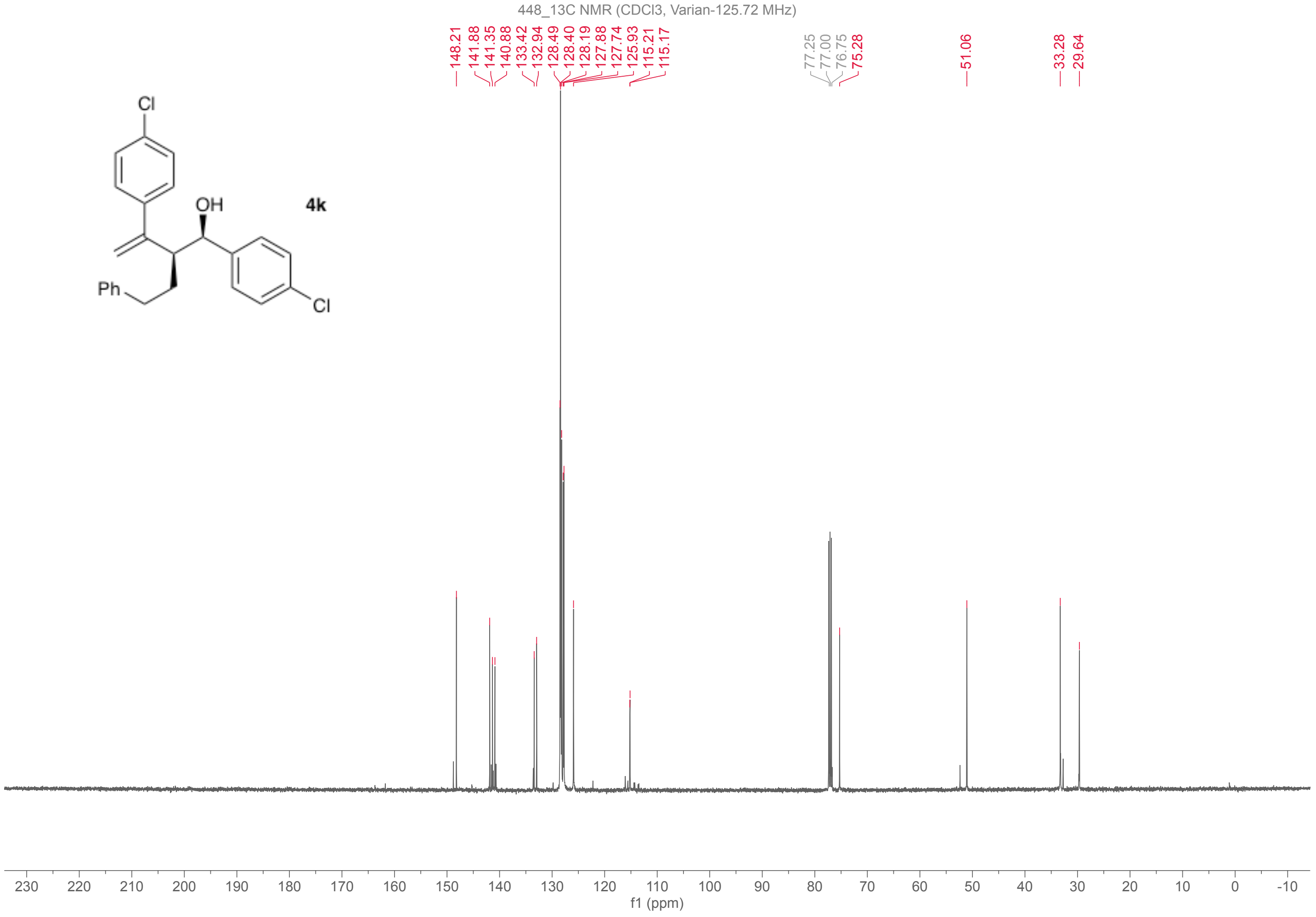
448_13C NMR (CDCl3, Varian-125.72 MHz)
148.21
141.88
141.35
140.88
133.42
132.94
128.49
128.40
128.19
127.88
127.74
125.93
115.21
115.17
77.25
77.00
76.75
75.28
51.06
33.28
29.64
Cl
OH
4k
Ph
Cl
230
220
210
200
190
180
170
160
150
140
130
120
110
100
90
80
70
60
50
40
30
20
10
0
-10
f1 (ppm)

446_1H NMR (CDCl3, Varian-499.94 MHz)

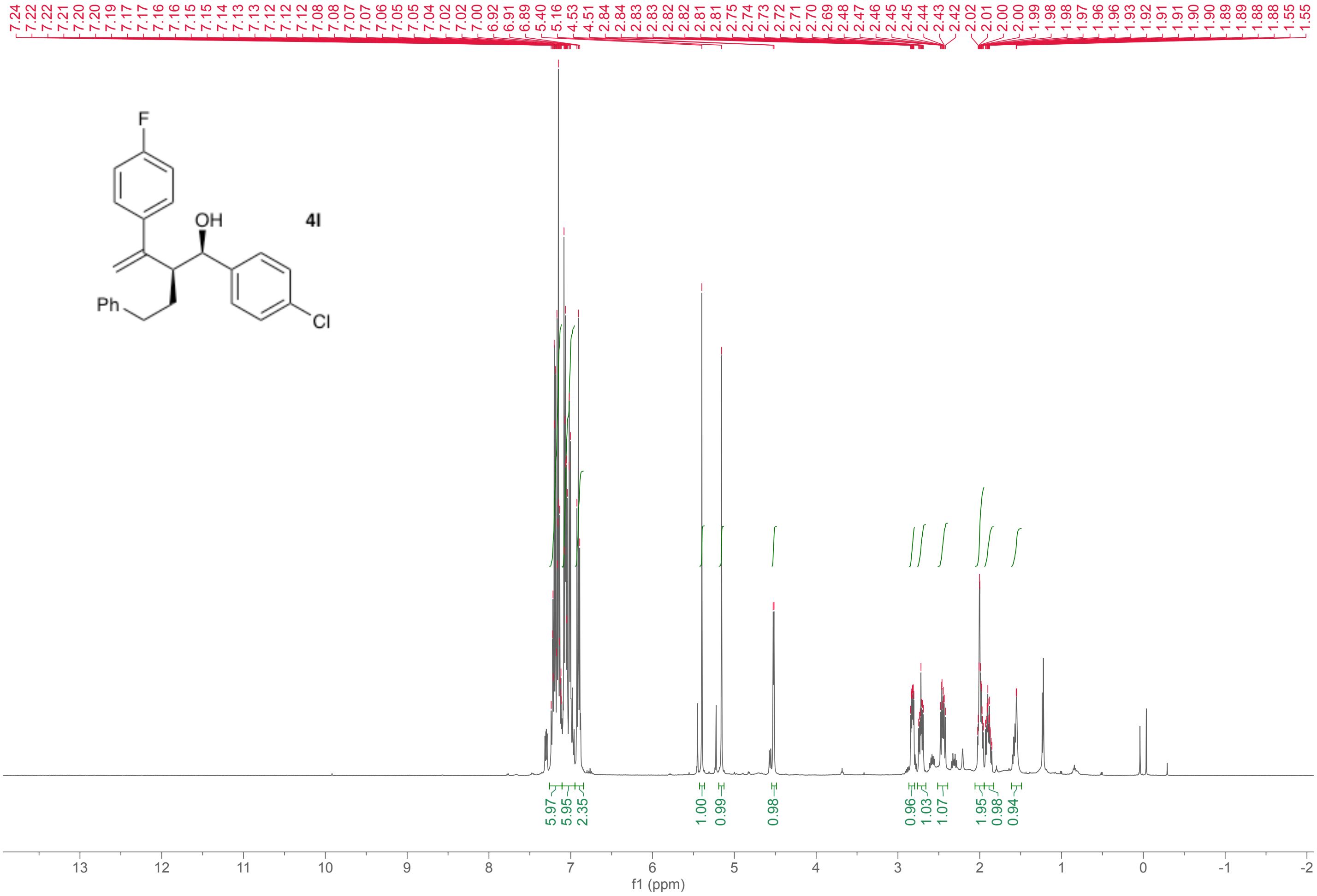

446_13C NMR (CDCl3, Varian-125.72 MHz)

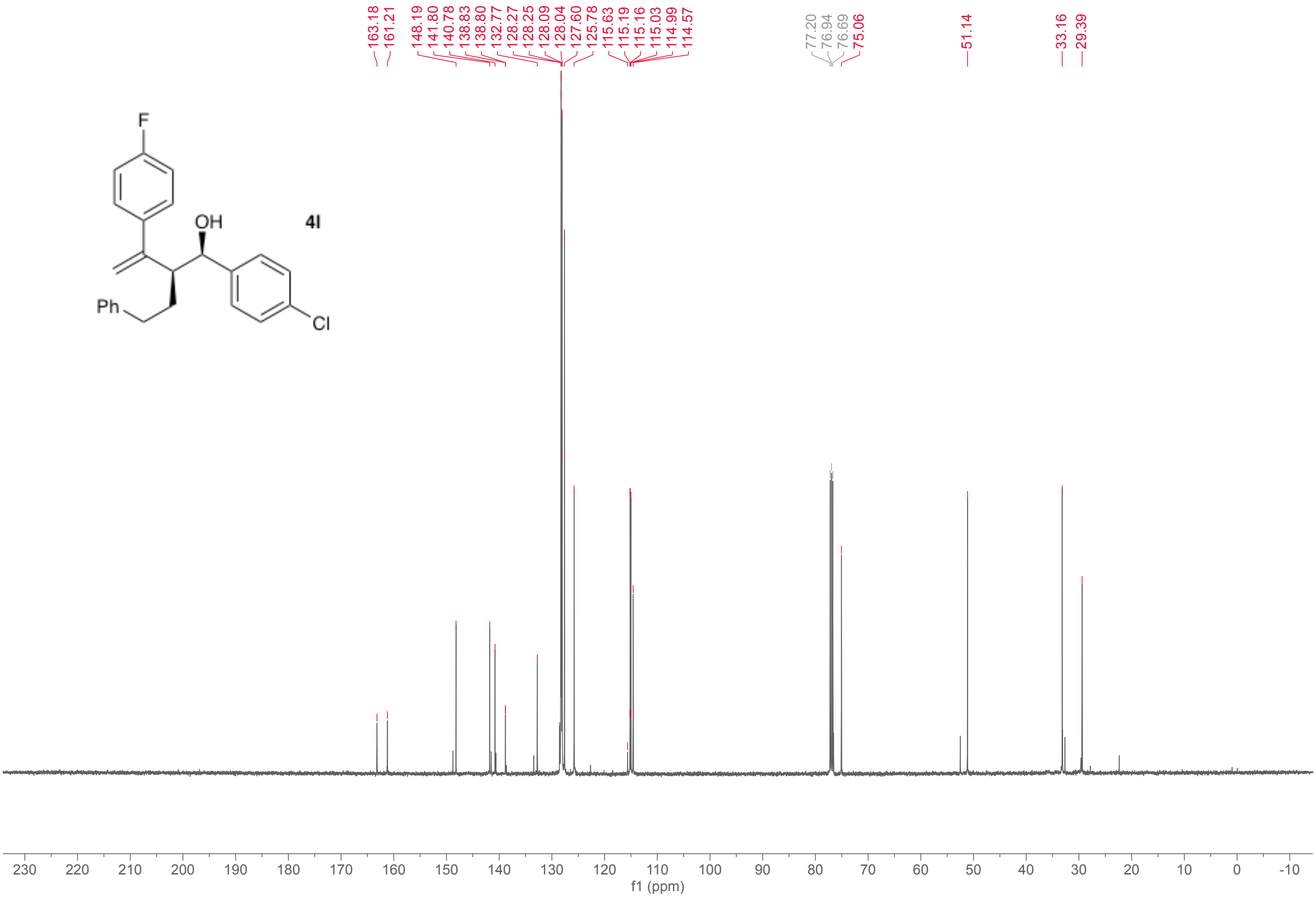

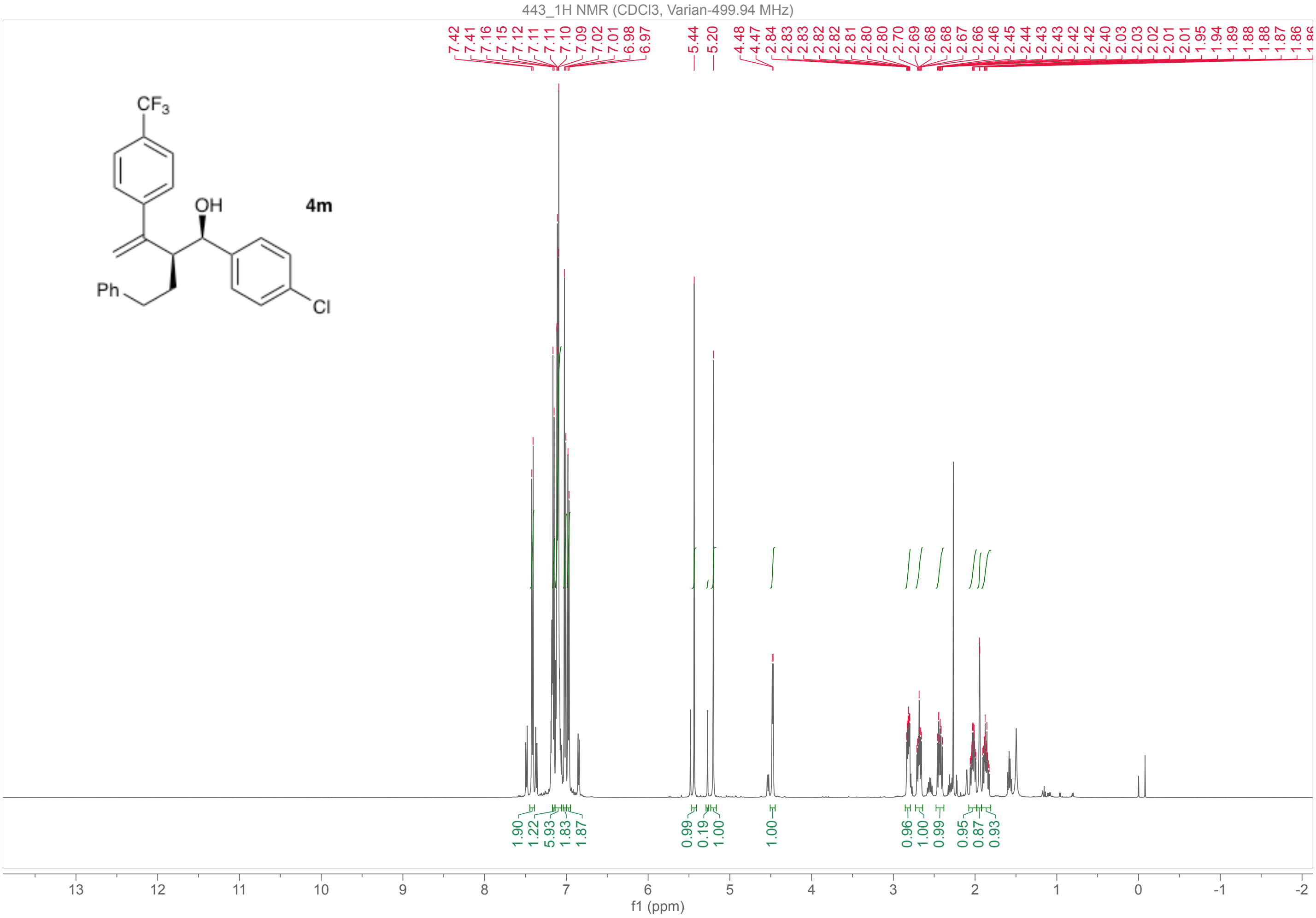
443_1H NMR (CDCl3, Varian-499.94 MHz)
7.42 7.41 7.16 7.15 7.12 7.11 7.11 7.10 7.09 7.02 7.01 6.98 6.97
5.44 5.20
4.48 4.47 2.84 2.83 2.83 2.82 2.82 2.81 2.80 2.80 2.70 2.69 2.68 2.68 2.67 2.66 2.46 2.45 2.44 2.43 2.43 2.42 2.42 2.40 2.03 2.03 2.02 2.01 2.01 1.95 1.94 1.89 1.88 1.88 1.87 1.86
CF3
OH
4m
Ph
Cl
1.90 1.22 5.93 1.83 1.87
0.99 0.19 1.00
1.00
0.96 1.00 0.99 0.95 0.87 0.93
f1 (ppm)

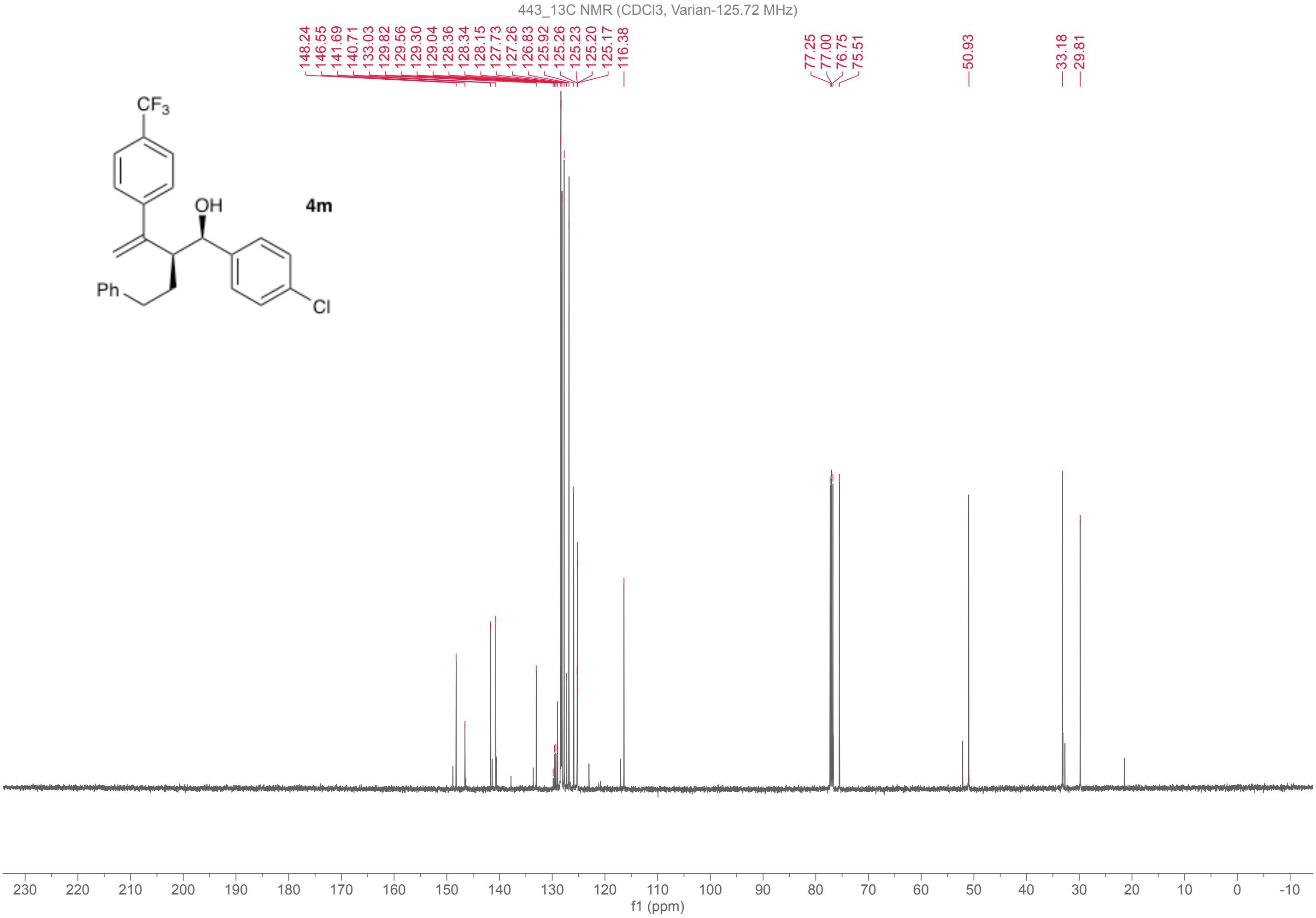
443_13C NMR (CDCl3, Varian-125.72 MHz)
148.24 146.55 141.69 140.71 133.03 129.82 129.56 129.30 129.04 128.36 128.34 128.15 127.73 127.26 126.83 125.92 125.26 125.23 125.20 125.17 116.38
77.25 77.00 76.75 75.51
50.93
33.18 29.81
CF3
OH
4m
Ph
Cl
f1 (ppm)

486_1H NMR (CDCl3, Varian-499.94 MHz)

4n

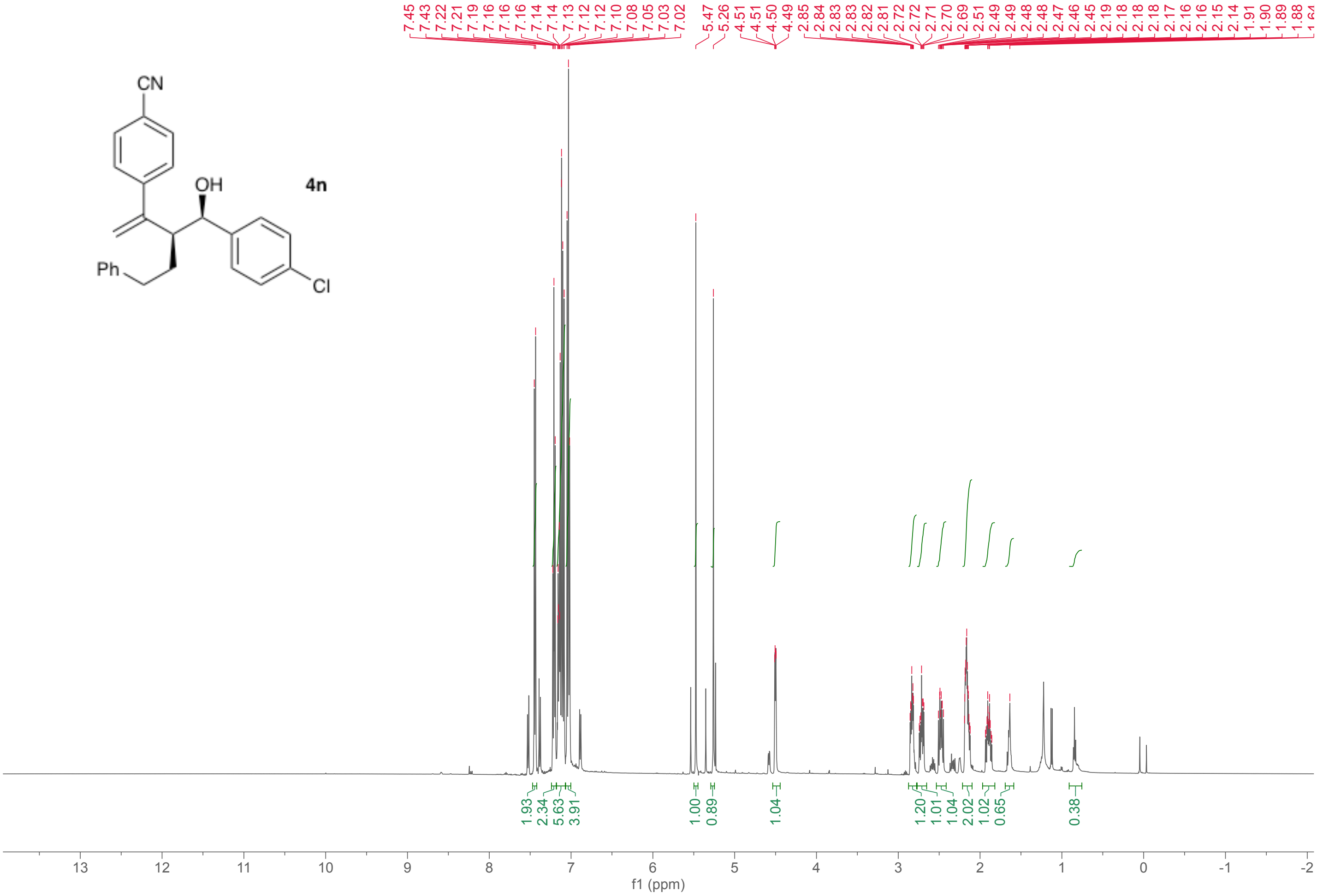

486_13C NMR (CDCl3, Varian-125.72 MHz)

4n

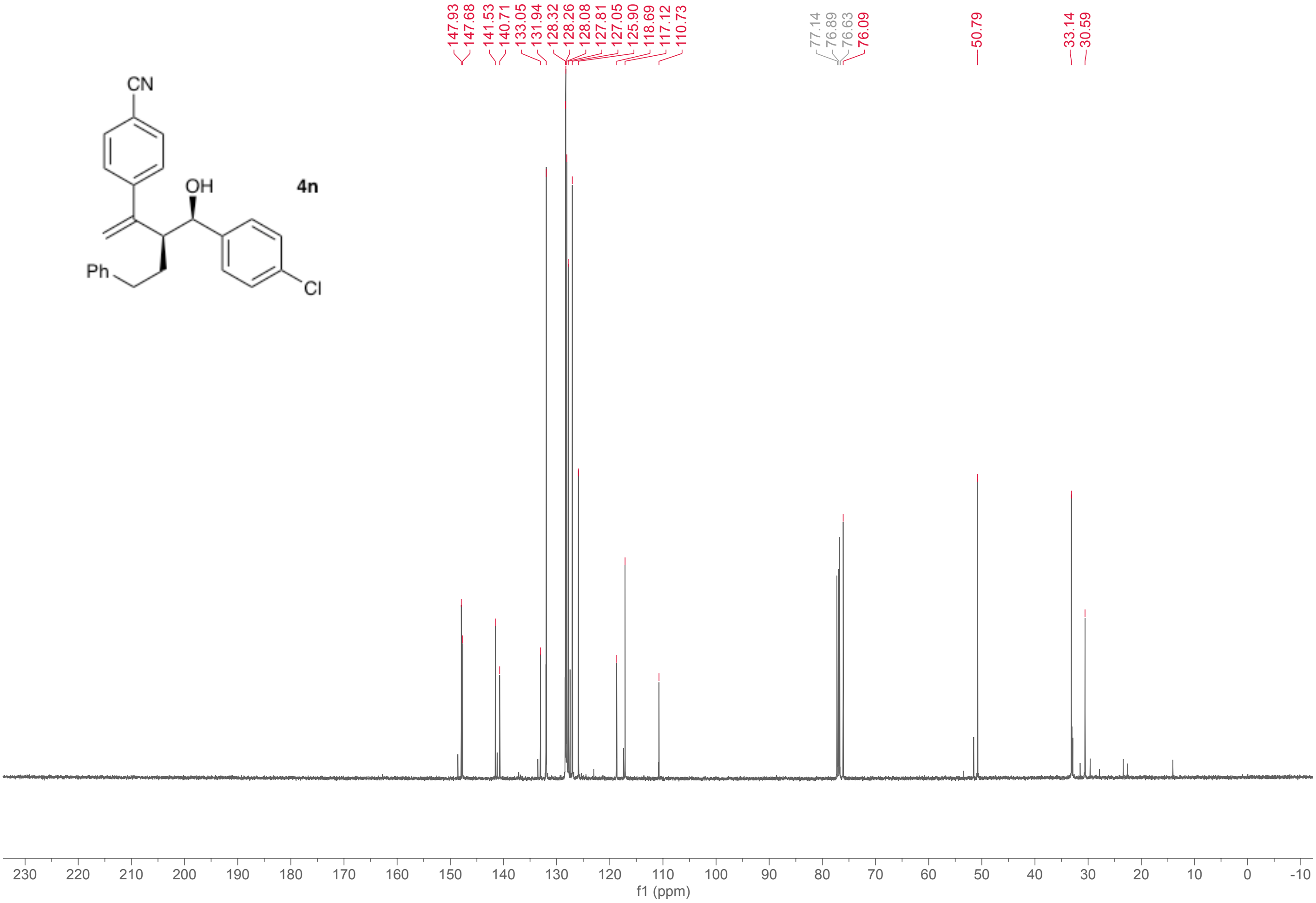

535_1H NMR spectra (499.94 MHz, CDCl3)

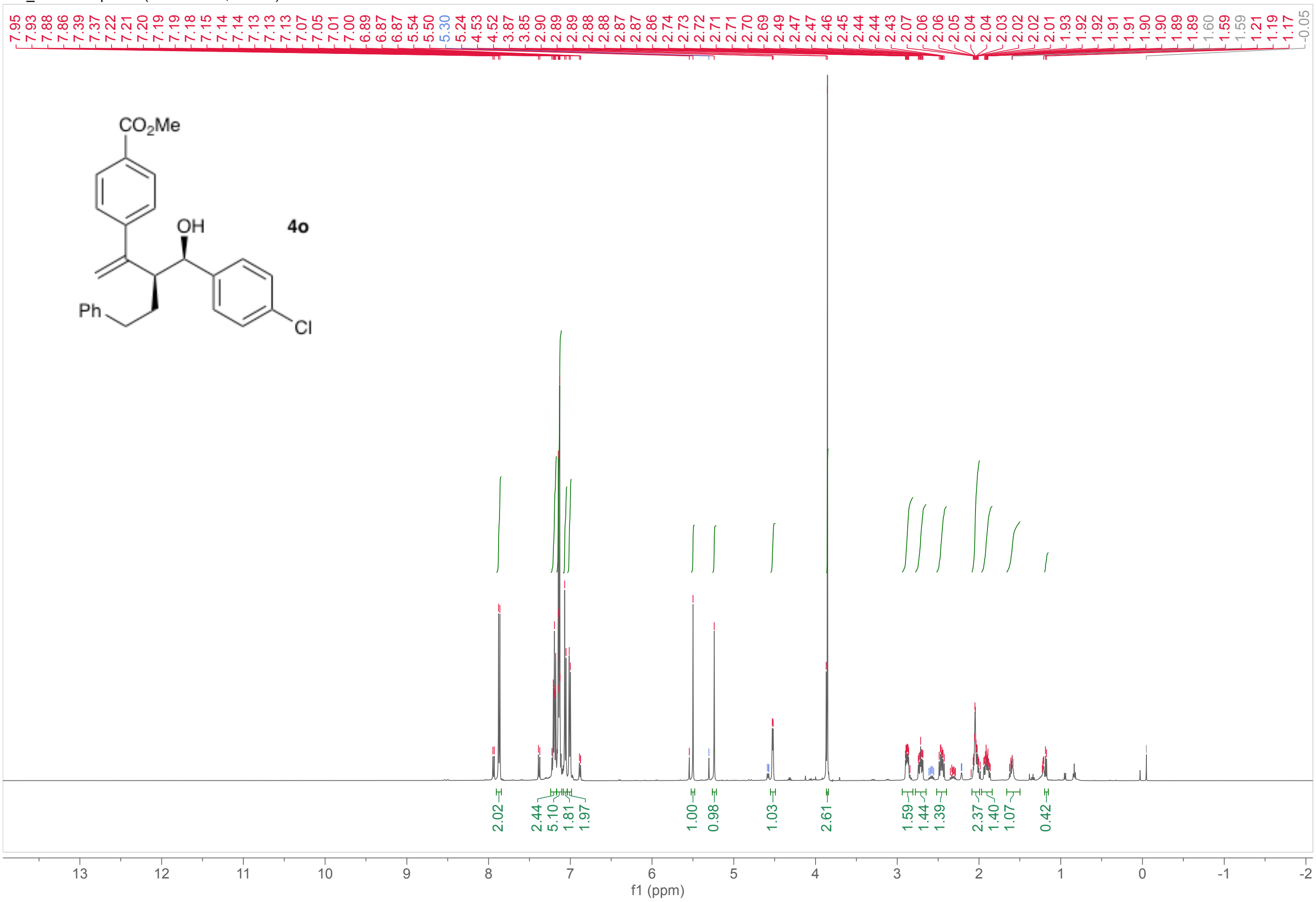

535_13C NMR spectra (125.72 MHz, CDCl3)

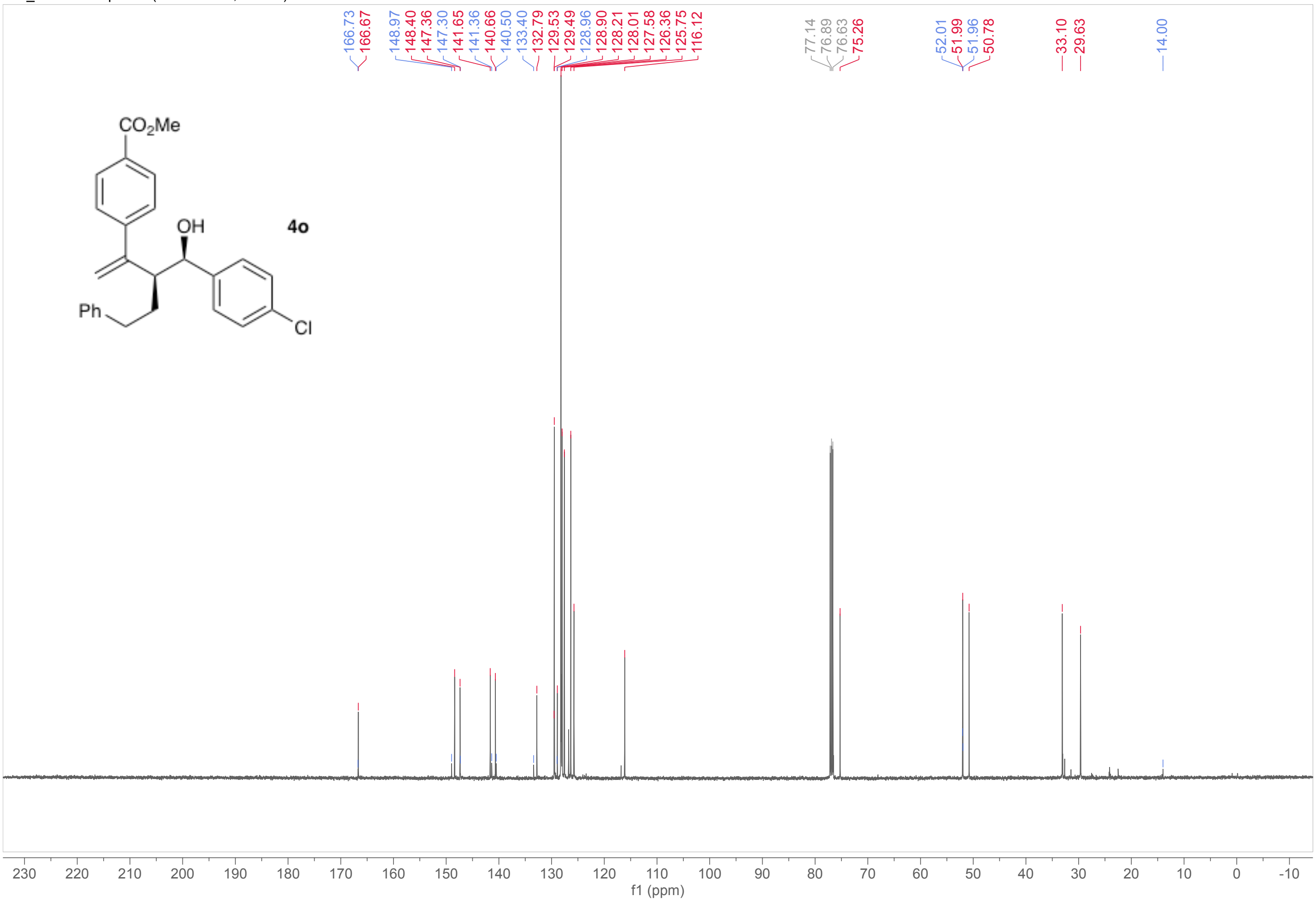

480_1H NMR (CDCl3, Varian-499.94 MHz)

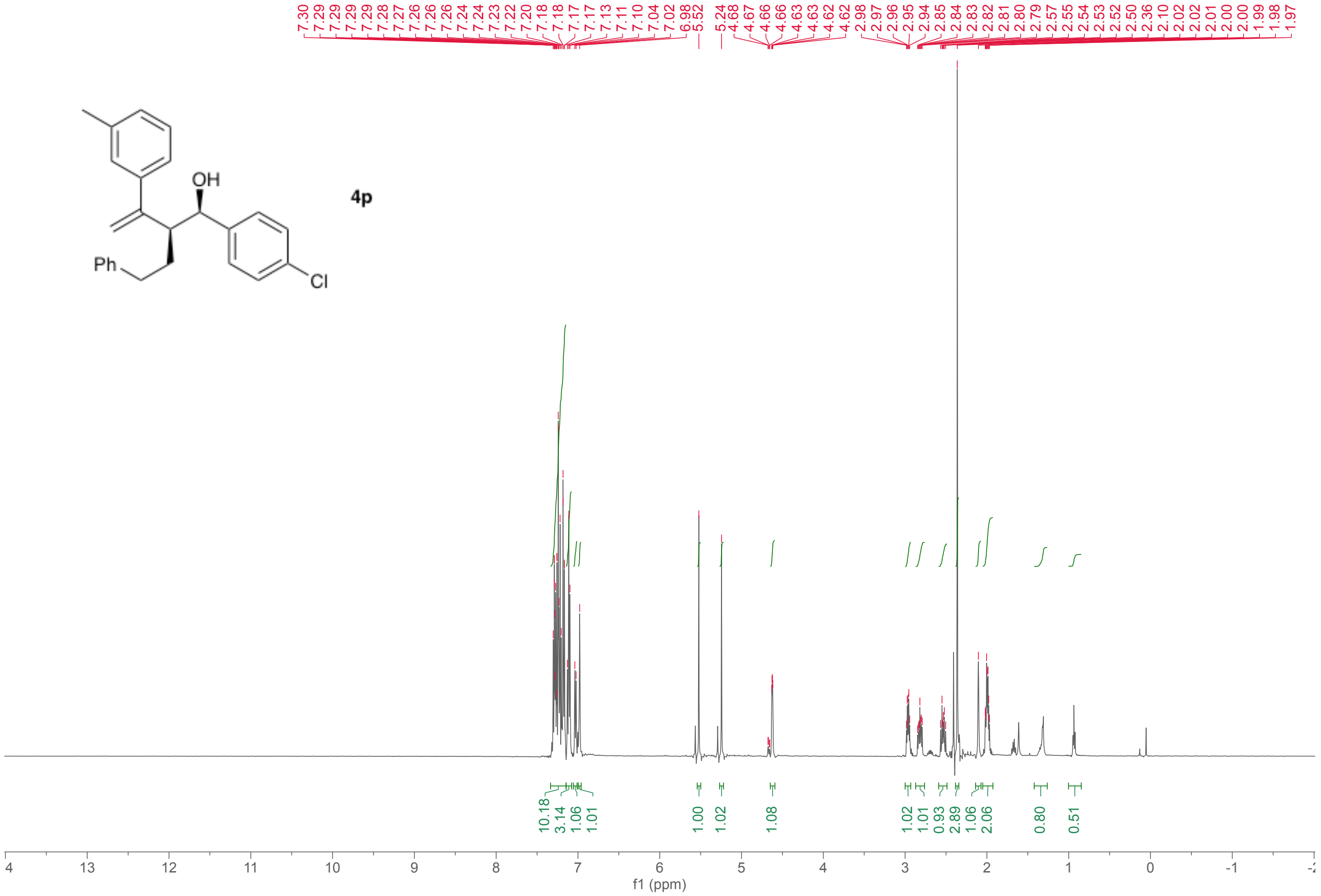

480_13C NMR (CDCl3, Varian-125.72 MHz)

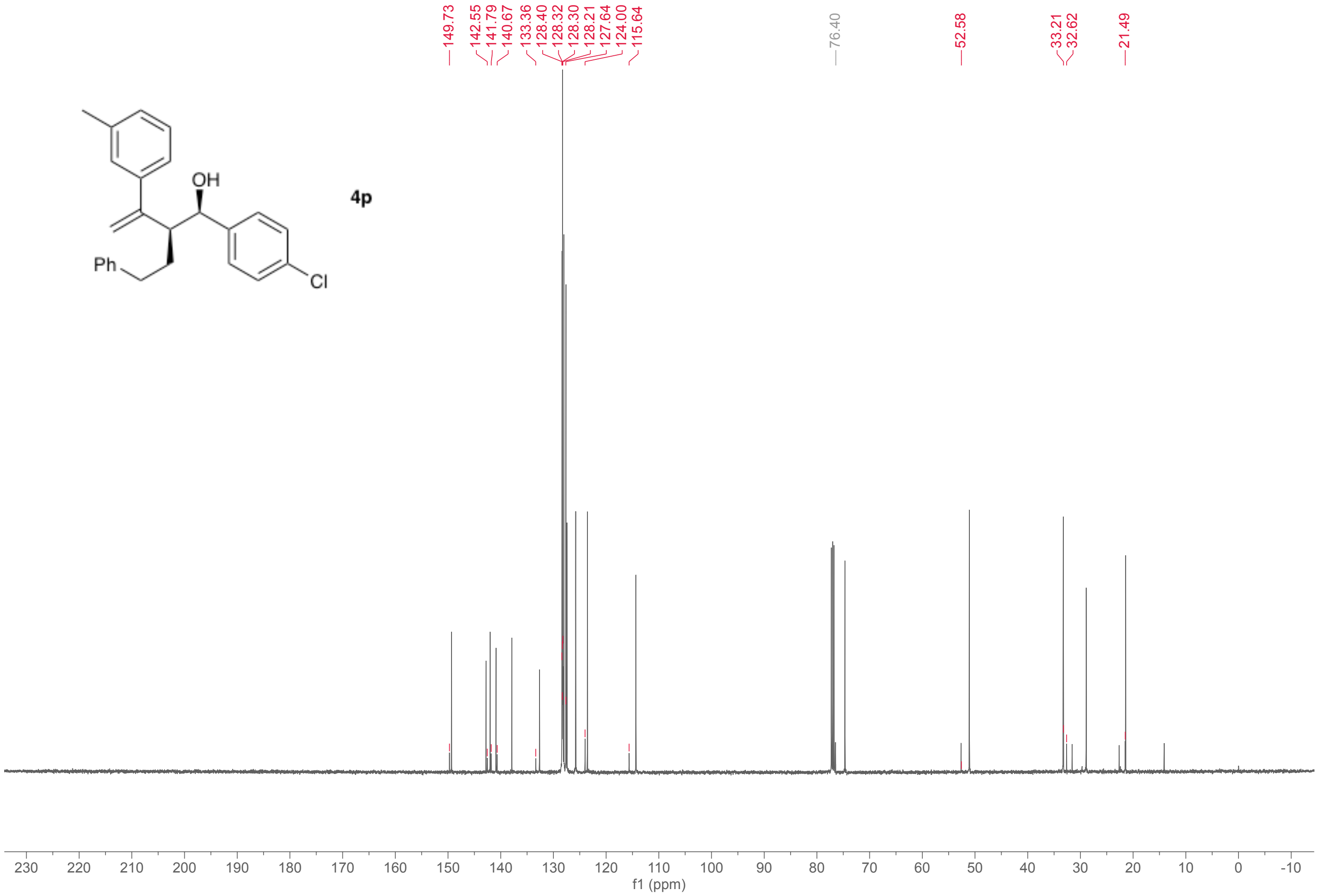

536_1H NMR spectra (499.94 MHz, CDCl3)

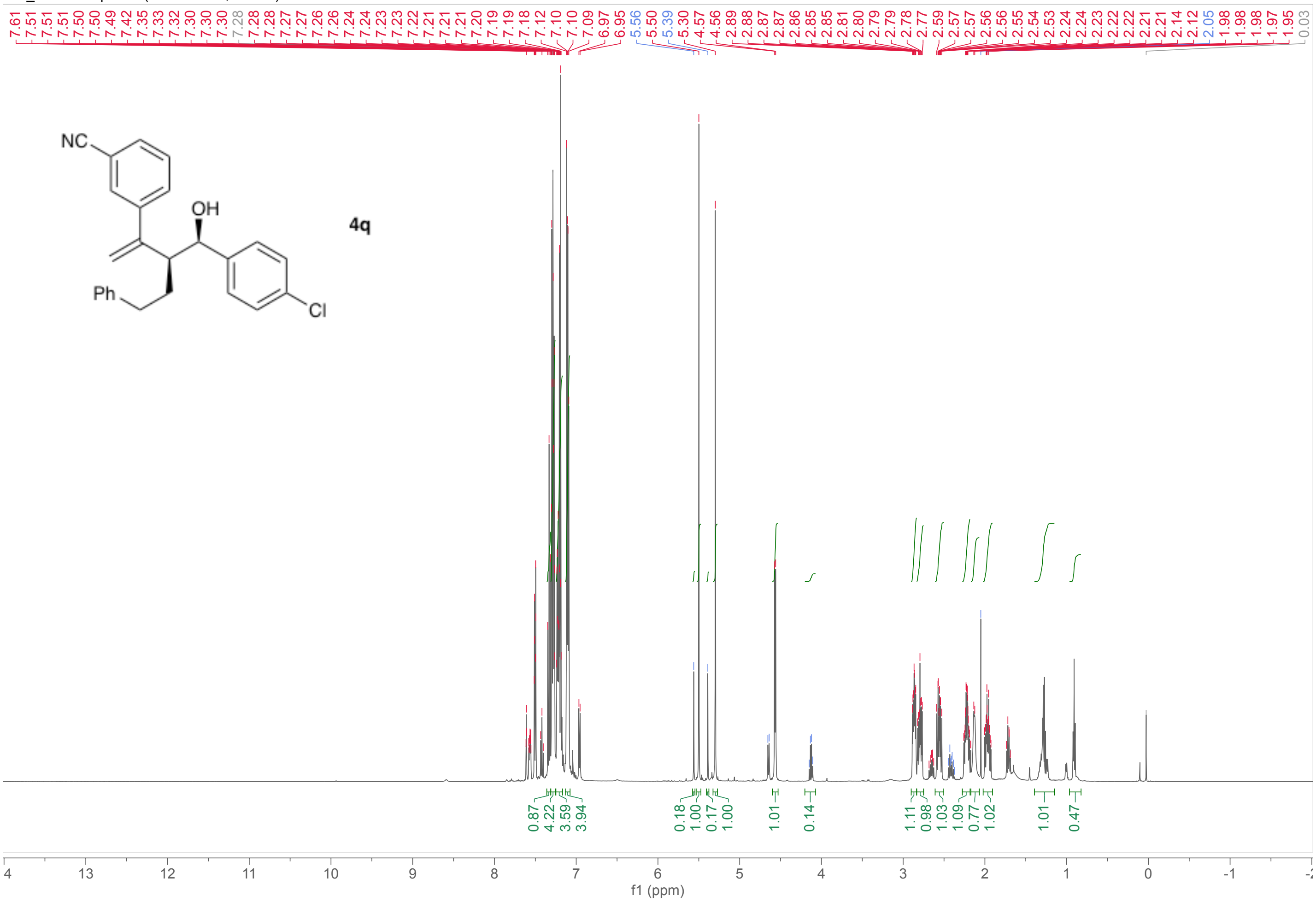

536_13C NMR spectra (125.72 MHz, cdcl3)

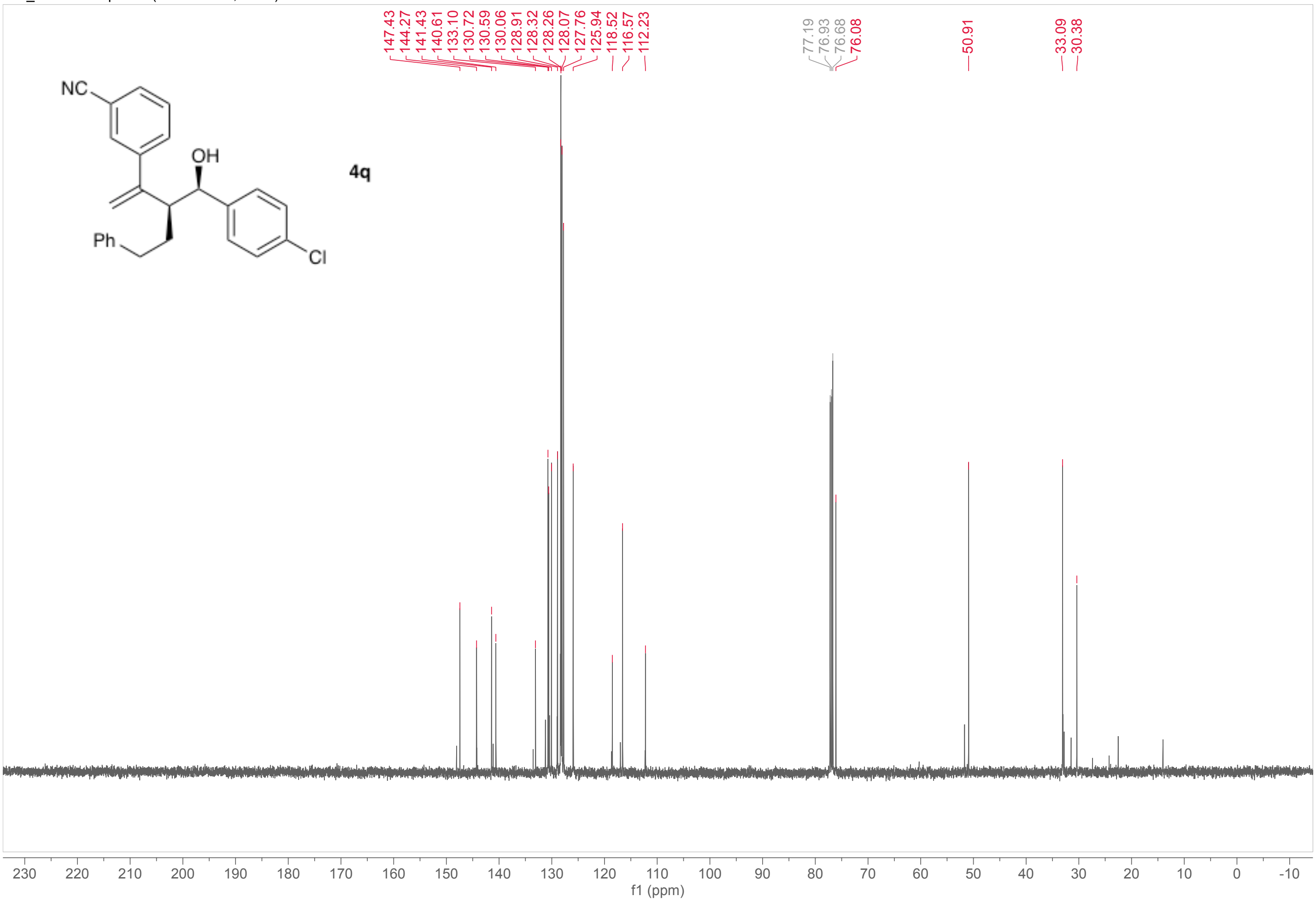

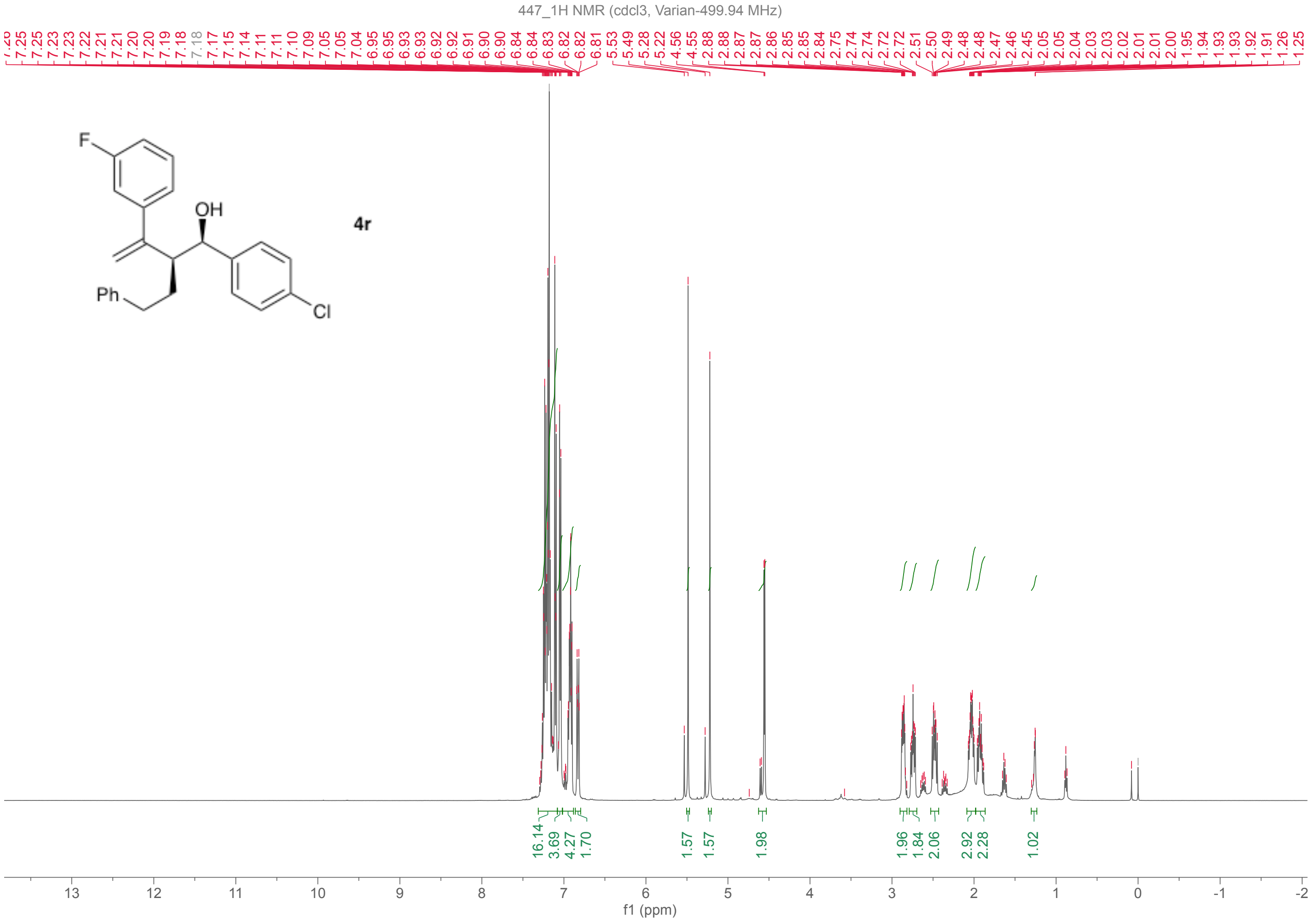
447_1H NMR (cdcl3, Varian-499.94 MHz)
7.25 7.25 7.23 7.23 7.22 7.21 7.21 7.20 7.19 7.18 7.17 7.15 7.14 7.11 7.10 7.09 7.05 7.05 7.04 6.95 6.95 6.93 6.93 6.92 6.92 6.91 6.90 6.84 6.84 6.83 6.82 6.82 6.81 5.53 5.49 5.28 5.22 4.56 4.55 2.88 2.87 2.86 2.85 2.85 2.84 2.75 2.74 2.74 2.72 2.72 2.51 2.50 2.49 2.48 2.48 2.47 2.46 2.45 2.05 2.05 2.04 2.03 2.03 2.02 2.01 2.01 2.00 1.95 1.94 1.93 1.93 1.92 1.91 1.26 1.25
F
OH
Ph
Cl
4r
16.14 3.69 4.27 1.70 1.57 1.57 1.98 1.96 1.84 2.06 2.92 2.28 1.02
f1 (ppm)

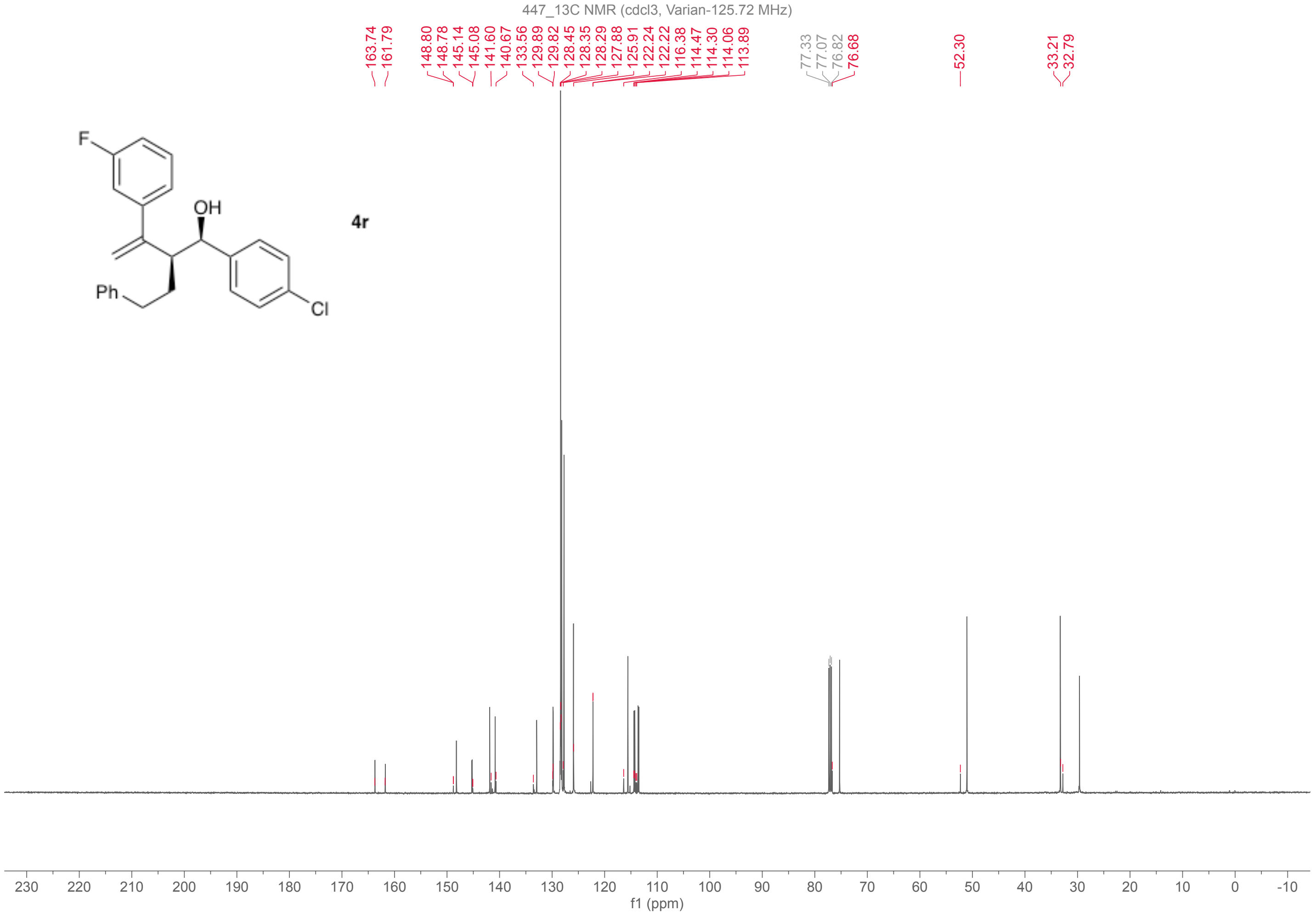
447_13C NMR (cdcl3, Varian-125.72 MHz)
163.74 161.79 148.80 148.78 145.14 145.08 141.60 140.67 133.56 129.89 129.82 128.45 128.35 128.29 127.88 125.91 122.24 122.22 116.38 114.47 114.30 114.06 113.89 77.33 77.07 76.82 76.68 52.30 33.21 32.79
F
OH
Ph
Cl
4r
f1 (ppm)

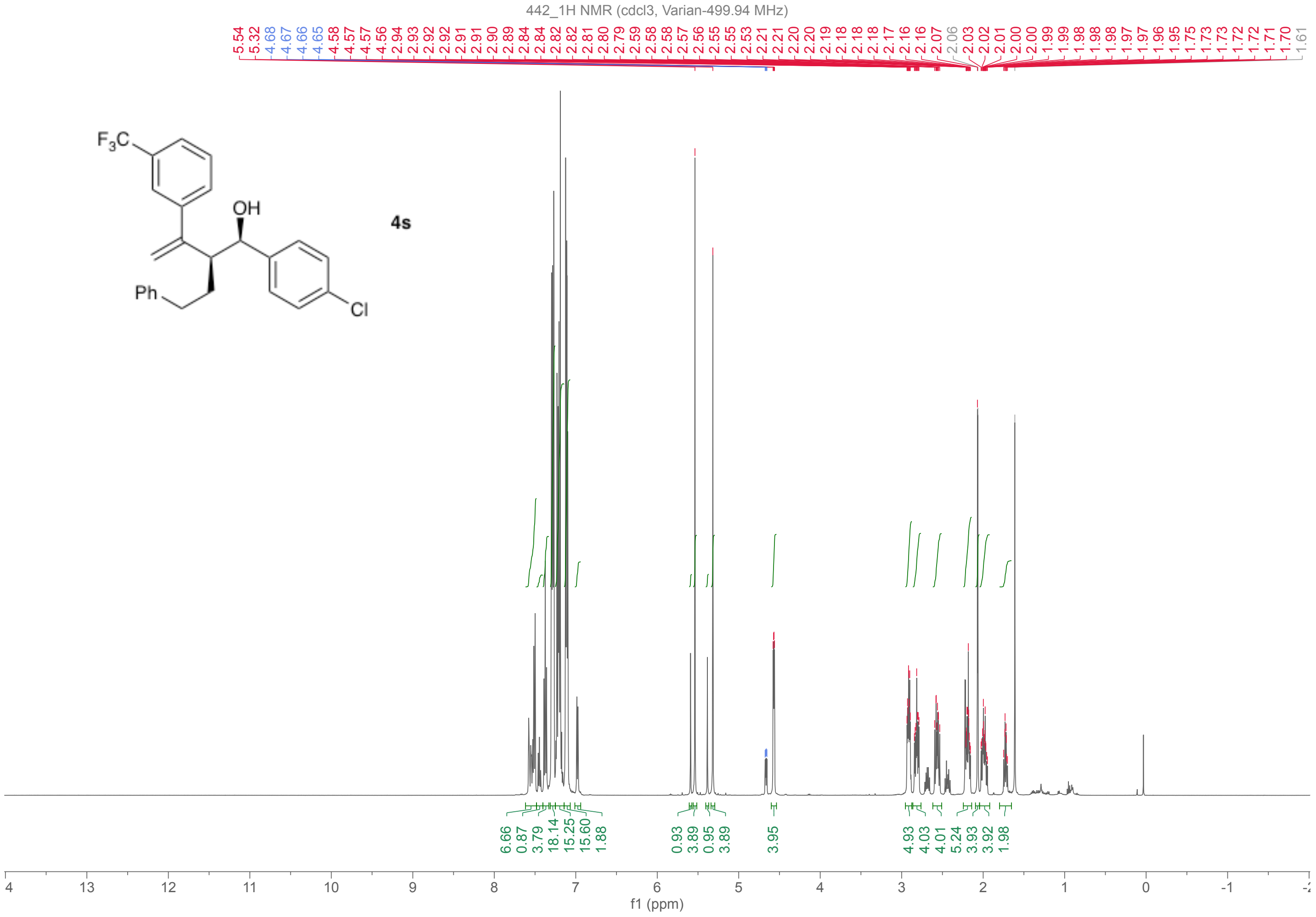
442_1H NMR (cdcl3, Varian-499.94 MHz)
5.54 5.32 4.68 4.67 4.66 4.65 4.58 4.57 4.57 4.56 2.94 2.93 2.92 2.92 2.91 2.91 2.90 2.89 2.84 2.84 2.82 2.82 2.81 2.80 2.79 2.59 2.58 2.58 2.57 2.56 2.55 2.55 2.53 2.21 2.21 2.20 2.20 2.19 2.18 2.18 2.18 2.17 2.16 2.16 2.07 2.06 2.03 2.02 2.01 2.00 1.99 1.99 1.98 1.98 1.98 1.97 1.96 1.95 1.75 1.73 1.73 1.72 1.72 1.71 1.70 1.61
F3C
OH
Ph
Cl
4s
6.66 0.87 3.79 18.14 15.25 15.60 1.88
0.93 3.89 0.95 3.89
3.95
4.93 4.03 4.01 5.24 3.93 3.92 1.98
14 13 12 11 10 9 8 7 6 5 4 3 2 1 0 -1 -2
f1 (ppm)

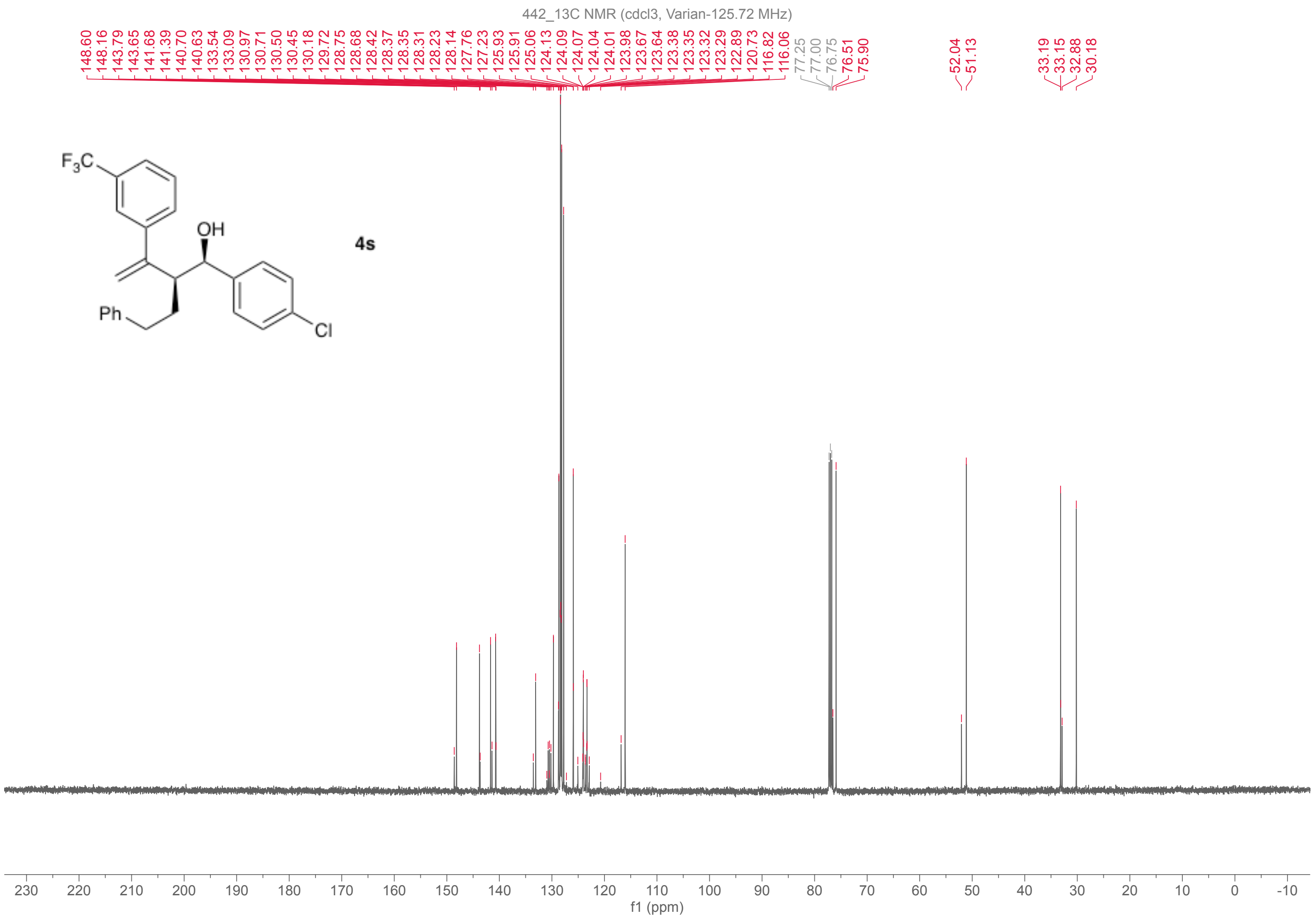
442_13C NMR (cdcl3, Varian-125.72 MHz)
148.60 148.16 143.79 143.65 141.68 141.39 140.70 140.63 133.54 133.09 130.97 130.71 130.50 130.45 130.18 129.72 128.75 128.68 128.42 128.37 128.35 128.31 128.23 128.14 127.76 127.23 125.93 125.91 125.06 124.13 124.09 124.07 124.04 124.01 123.98 123.67 123.64 123.38 123.35 123.32 123.29 122.89 120.73 116.82 116.06 77.25 77.00 76.75 76.51 75.90
52.04 51.13
33.19 33.15 32.88 30.18
F3C
OH
Ph
Cl
4s
230 220 210 200 190 180 170 160 150 140 130 120 110 100 90 80 70 60 50 40 30 20 10 0 -10
f1 (ppm)

481_1H NMR (cdcl3, 499.94 MHz)

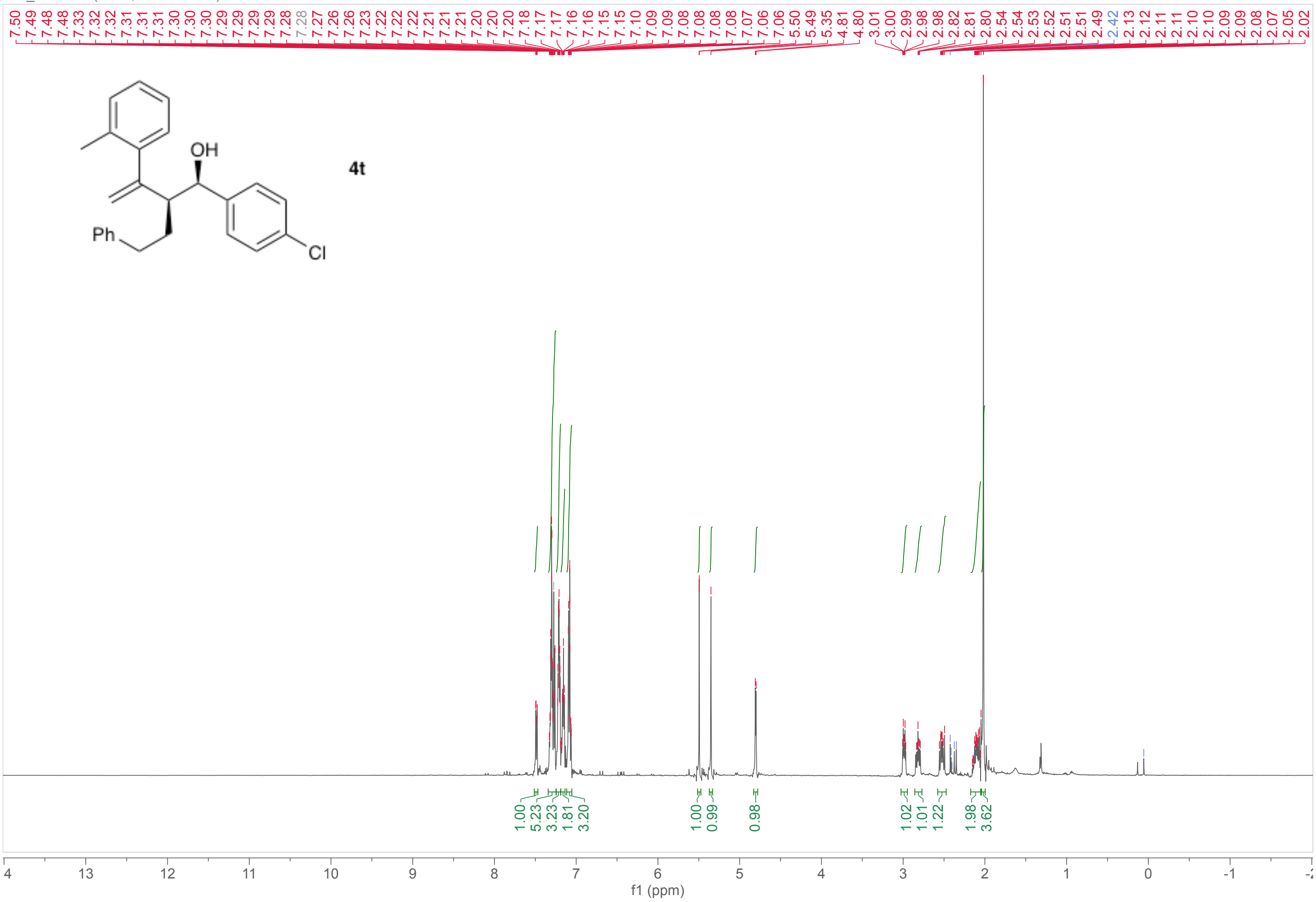

481_13C NMR (cdcl3, 125.72 MHz)

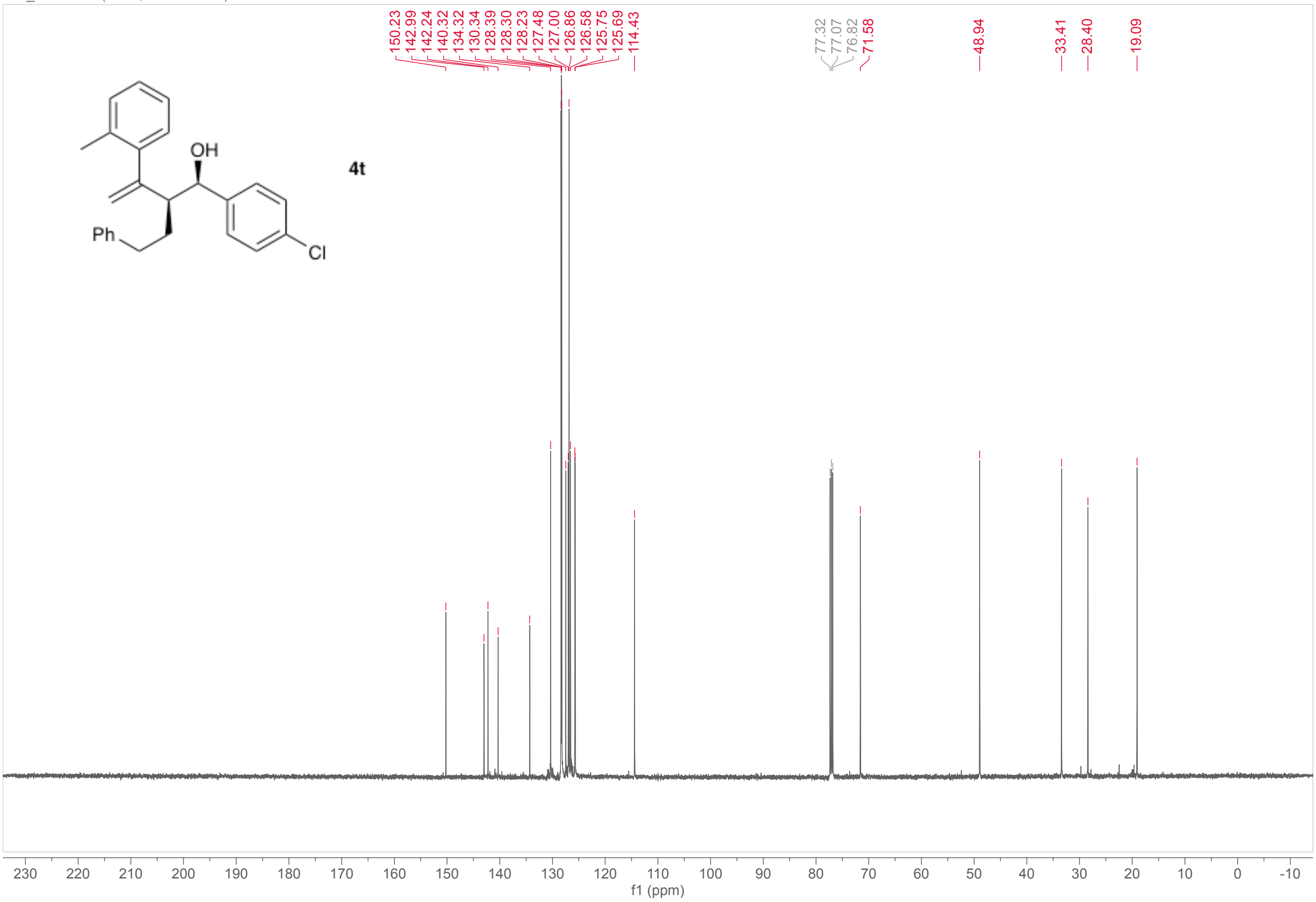

147_1H NMR (cdcl3, 499.94 MHz)

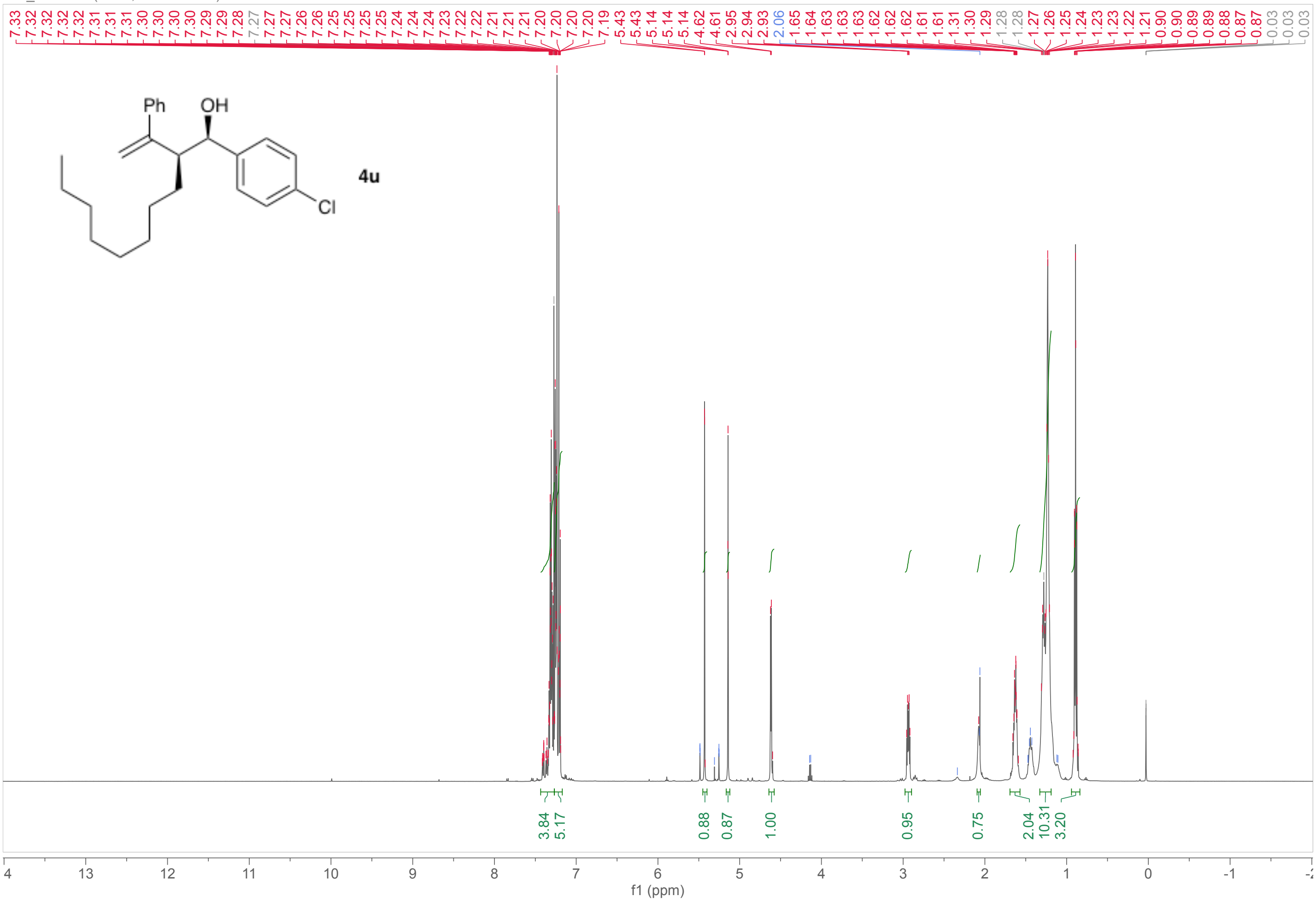

147_13C NMR (cdcl3, 100.57 MHz)

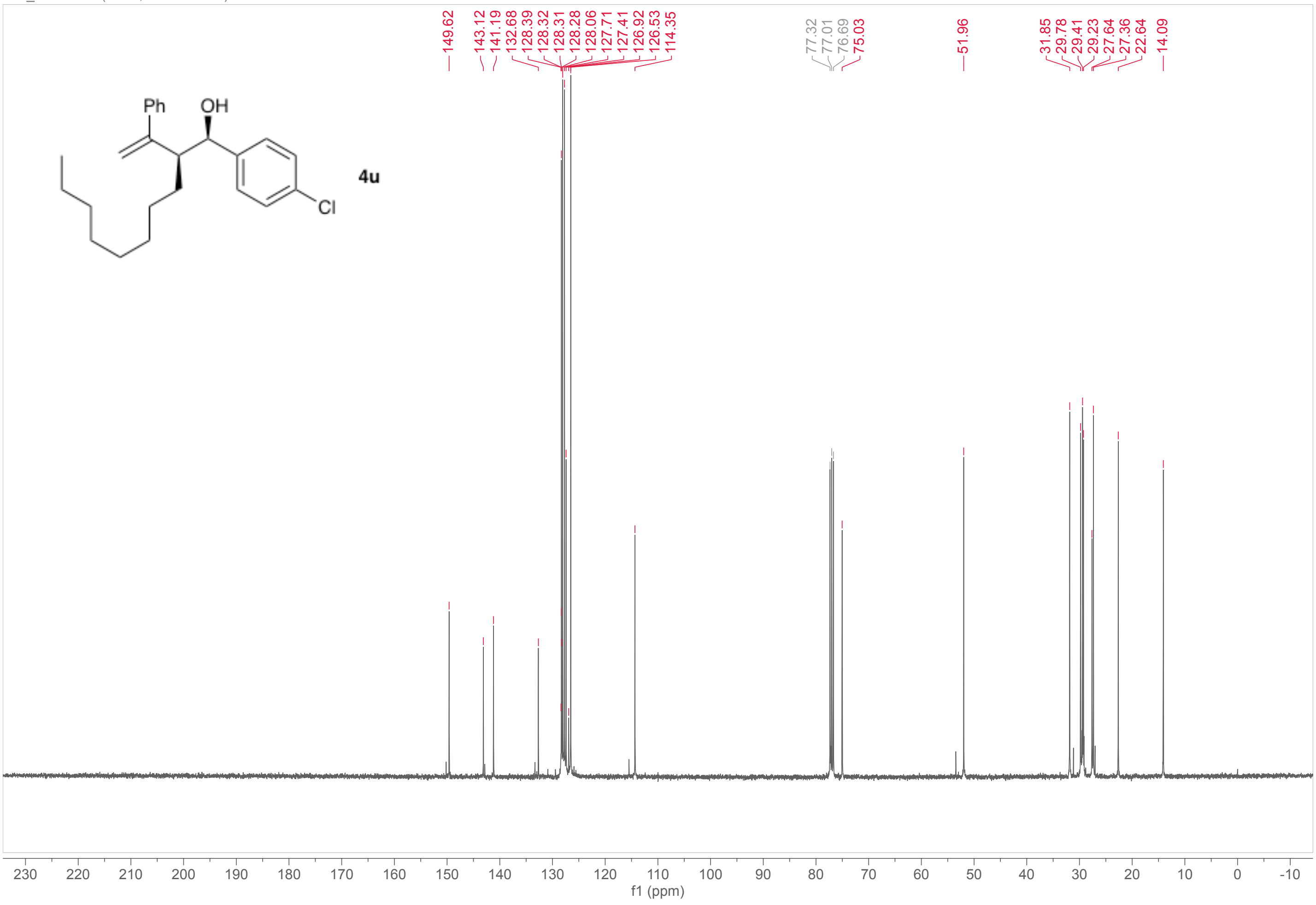

142_1H NMR (cdcl3, 399.90 MHz)

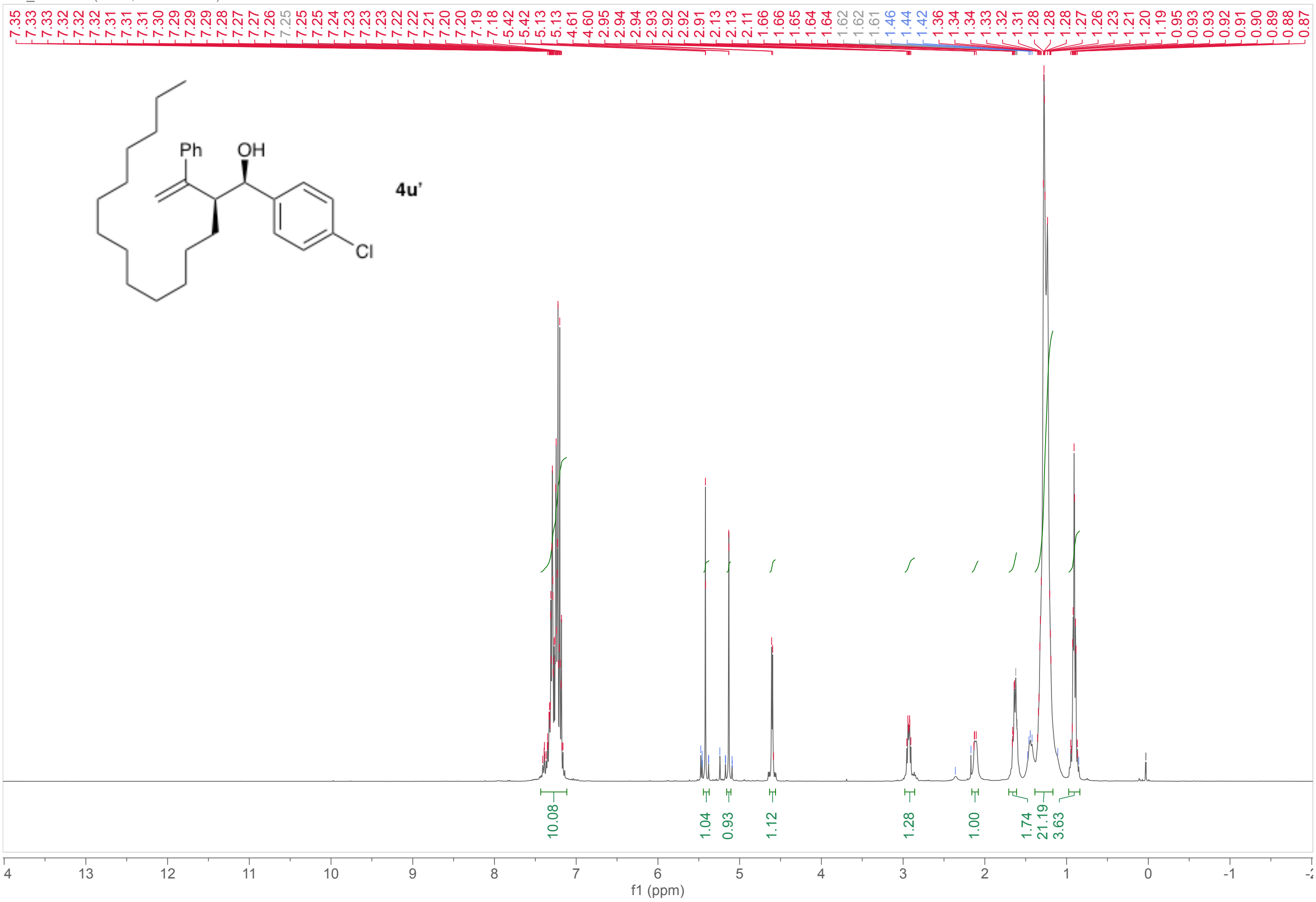

142_13C NMR (cdcl3, 100.57 MHz)

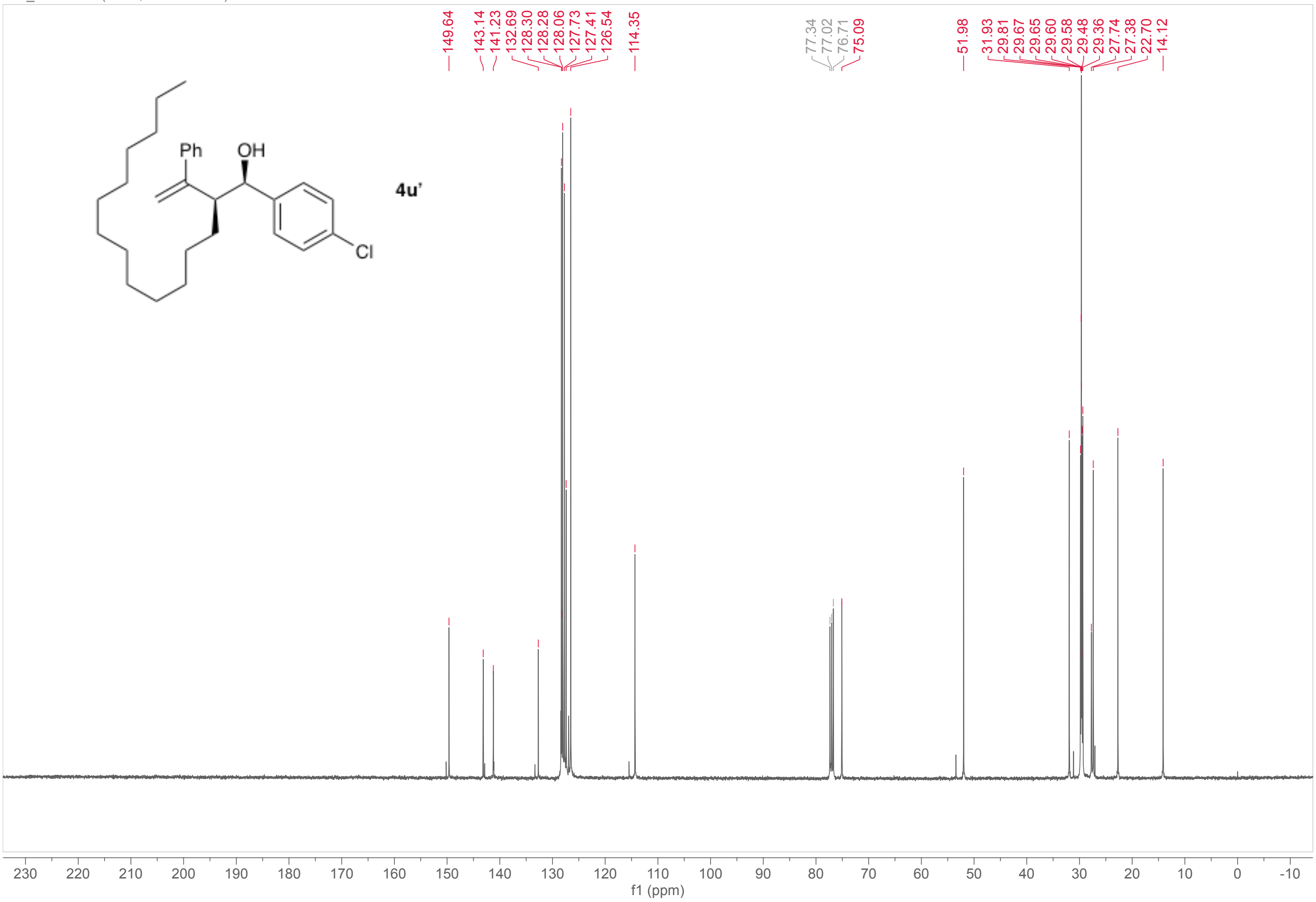

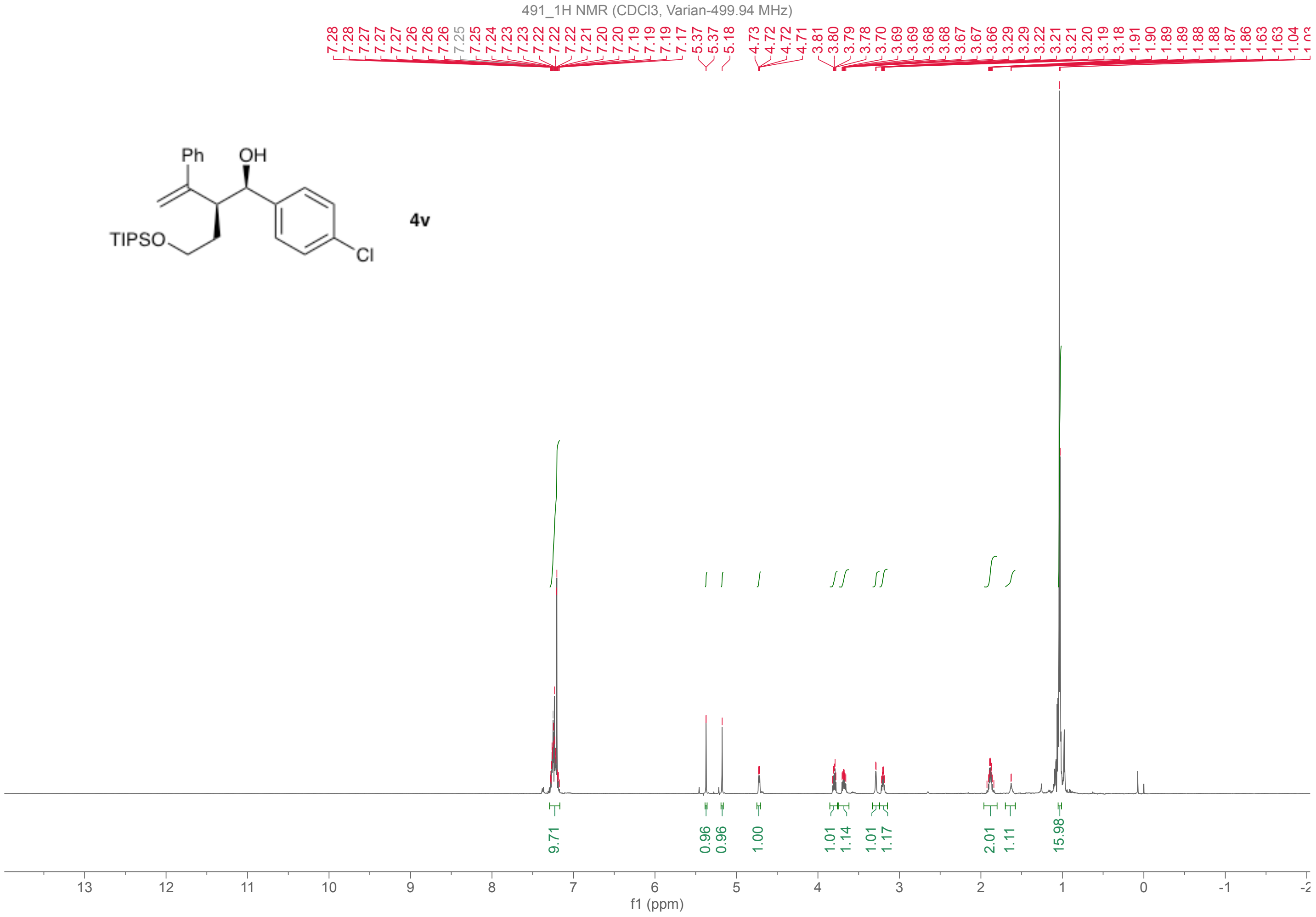

491_1H NMR (CDCl3, Varian-499.94 MHz)
7.28 7.28 7.27 7.27 7.27 7.26 7.26 7.25 7.25 7.24 7.23 7.23 7.22 7.22 7.22 7.21 7.20 7.20 7.19 7.19 7.17 5.37 5.37 5.18 4.73 4.72 4.72 4.71 3.81 3.80 3.79 3.78 3.70 3.69 3.69 3.68 3.68 3.67 3.67 3.66 3.29 3.29 3.22 3.21 3.21 3.20 3.19 3.18 1.91 1.90 1.89 1.88 1.88 1.87 1.86 1.63 1.04 [illegible]
Ph
OH
TIPSO
Cl
4v
9.71
0.96
0.96
1.00
1.01
1.14
1.01
1.17
2.01
1.11
15.98
13 12 11 10 9 8 7 6 5 4 3 2 1 0 -1 -2
f1 (ppm)

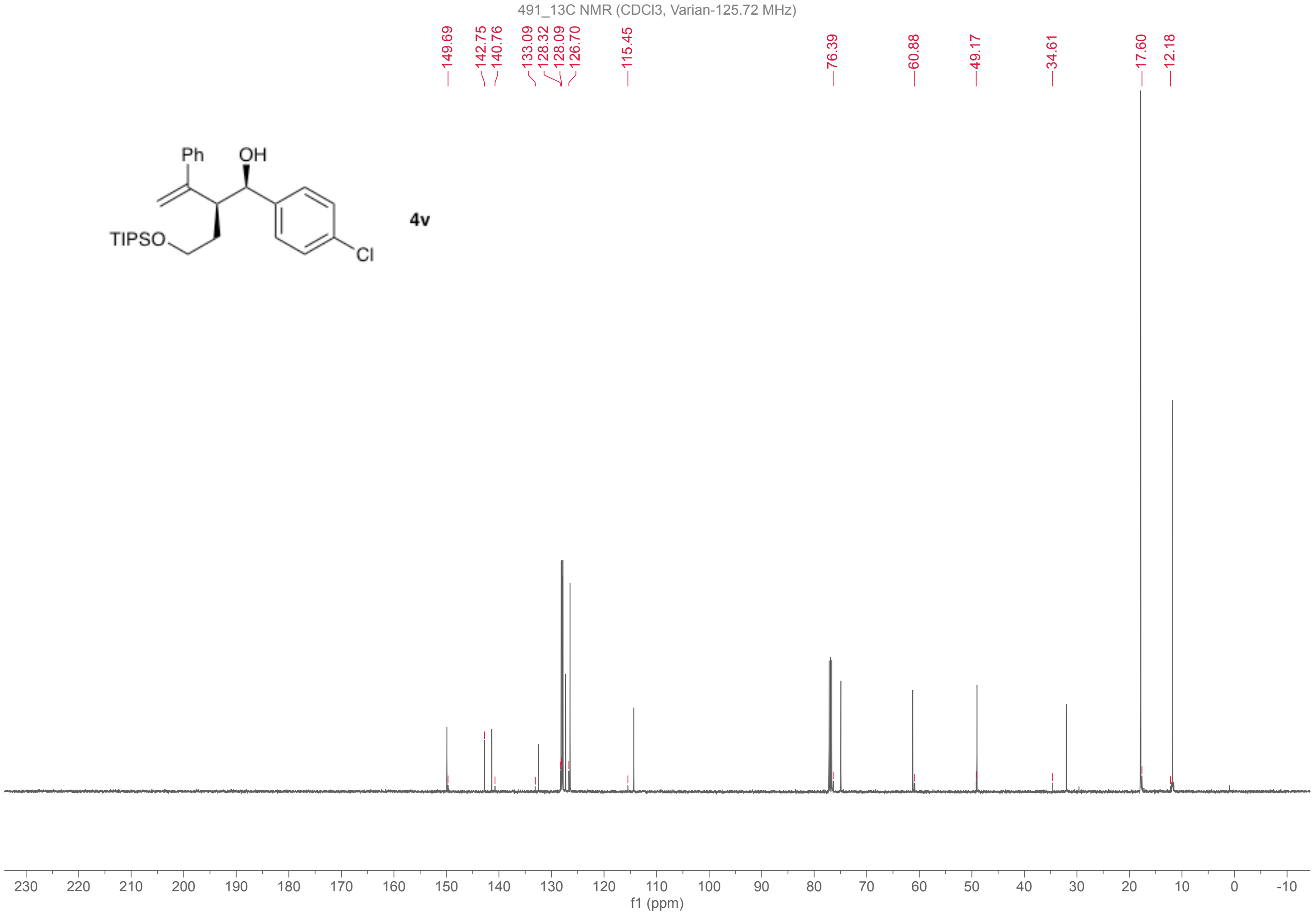

491_13C NMR (CDCl3, Varian-125.72 MHz)
149.69
142.75
140.76
133.09
128.32
128.09
126.70
115.45
76.39
60.88
49.17
34.61
17.60
12.18
Ph
OH
TIPSO
Cl
4v
230 220 210 200 190 180 170 160 150 140 130 120 110 100 90 80 70 60 50 40 30 20 10 0 -10
f1 (ppm)

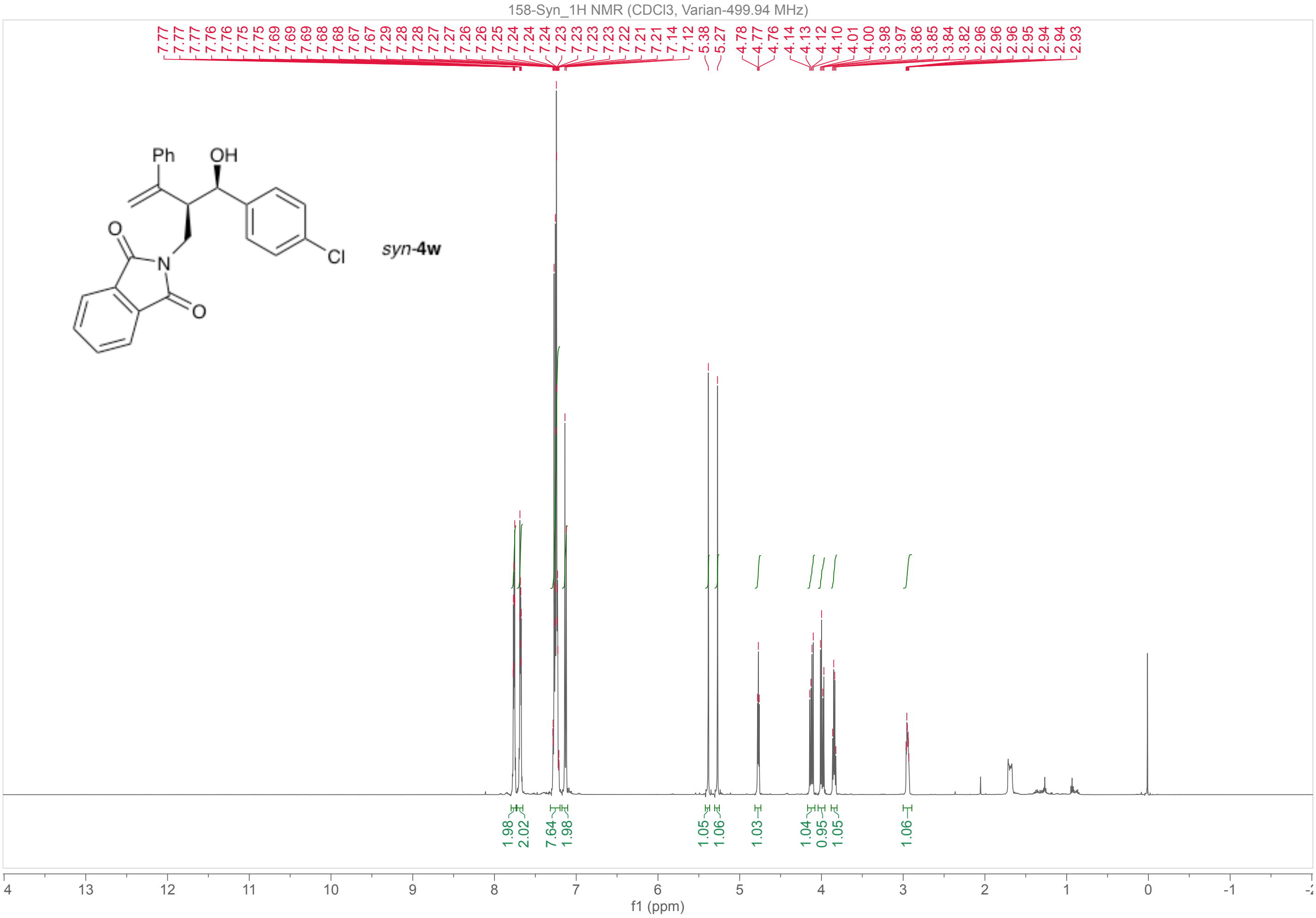
158-Syn_1H NMR (CDCl3, Varian-499.94 MHz)
7.77 7.77 7.76 7.75 7.75 7.69 7.69 7.69 7.68 7.68 7.67 7.67 7.29 7.28 7.28 7.27 7.27 7.26 7.26 7.25 7.24 7.24 7.24 7.23 7.23 7.23 7.23 7.22 7.22 7.21 7.21 7.14 7.12
5.38 5.27
4.78 4.77 4.76
4.14 4.13 4.12 4.10 4.01 4.00 3.98 3.97 3.86 3.85 3.84 3.82
2.96 2.96 2.96 2.95 2.94 2.94 2.93
Ph
OH
O
N
O
Cl
syn-4w
1.98 2.02 7.64 1.98 1.05 1.06 1.03 1.04 0.95 1.05 1.06
f1 (ppm)

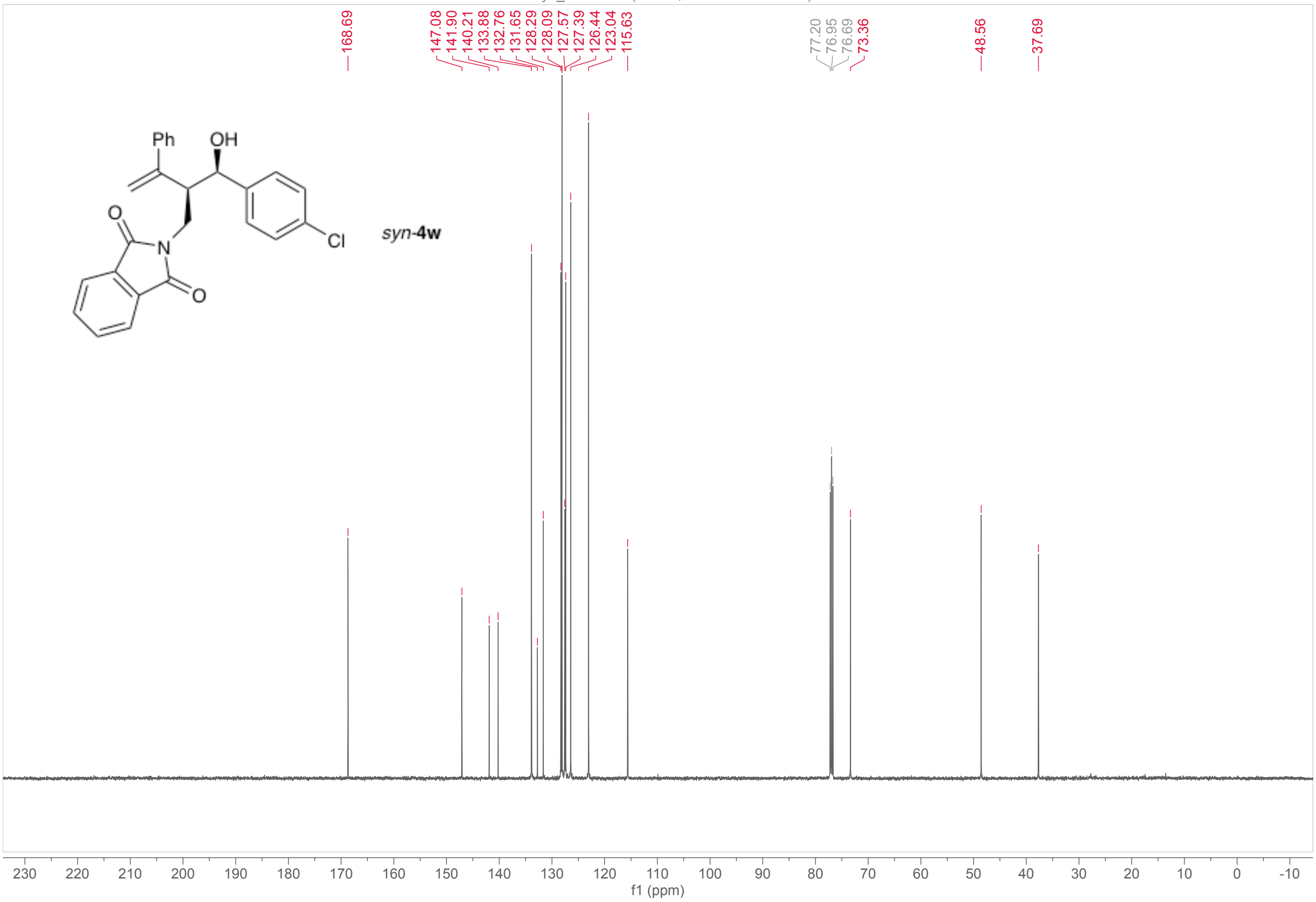
158-Syn_13C NMR (CDCl3, Varian-125.72 MHz)
168.69
147.08 141.90 140.21 133.88 132.76 131.65 128.29 128.09 127.57 127.39 126.44 123.04 115.63
77.20 76.95 76.69 73.36
48.56
37.69
Ph
OH
O
N
O
Cl
syn-4w
f1 (ppm)

158-Anti_1H NMR (CDCl3, Varian-499.94 MHz)

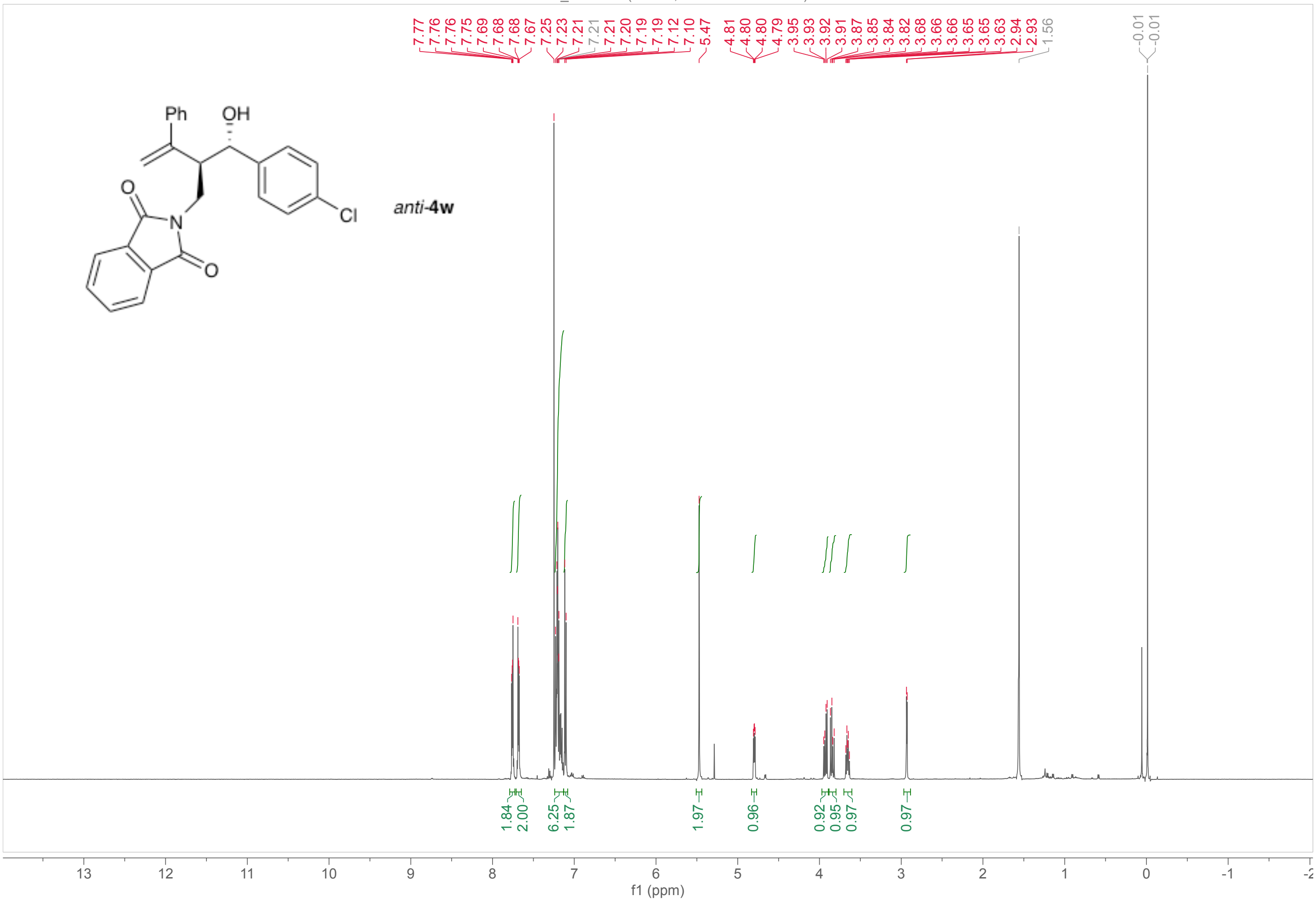

158-Anti_13C NMR (CDCl3, Varian-125.72 MHz)

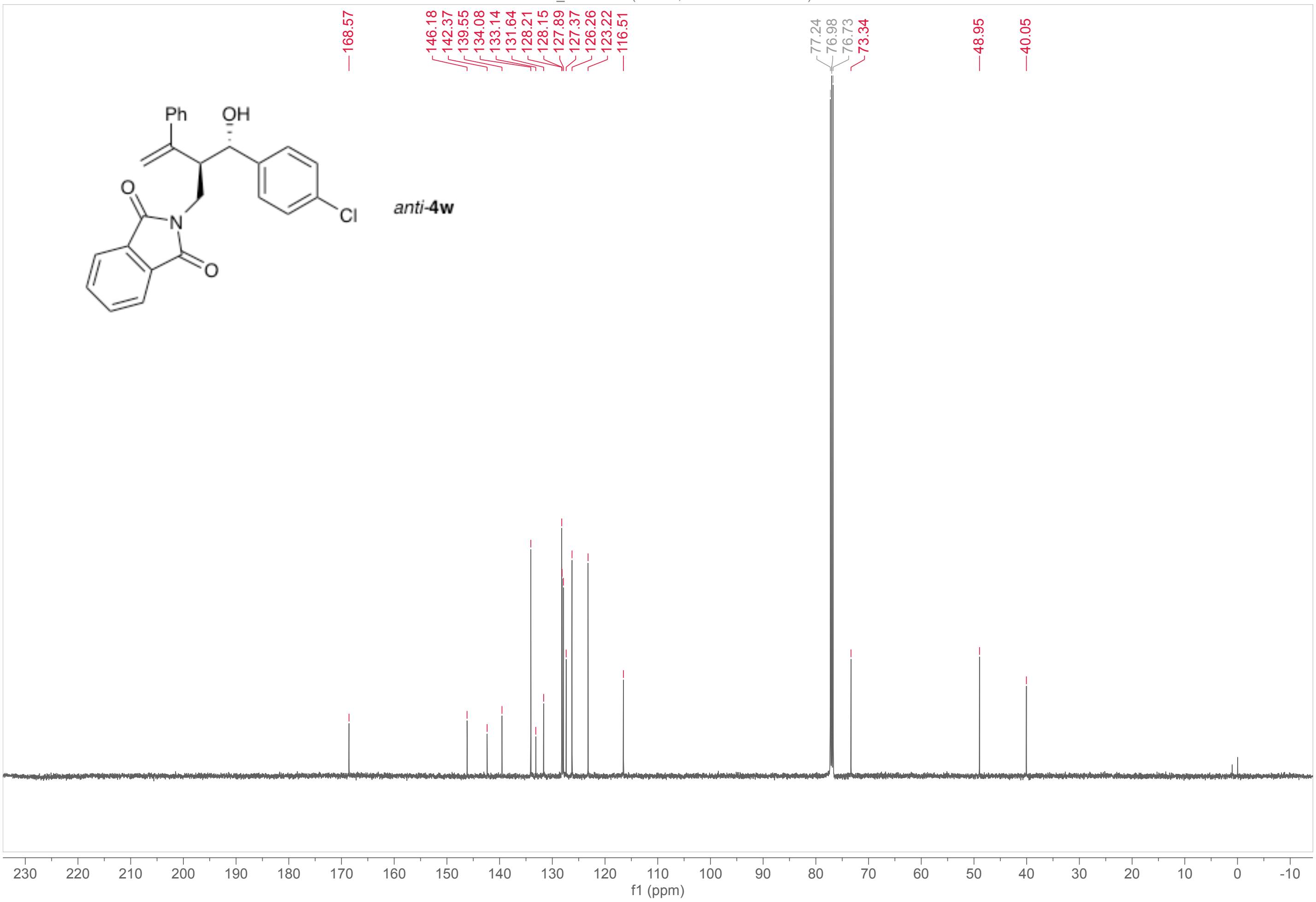

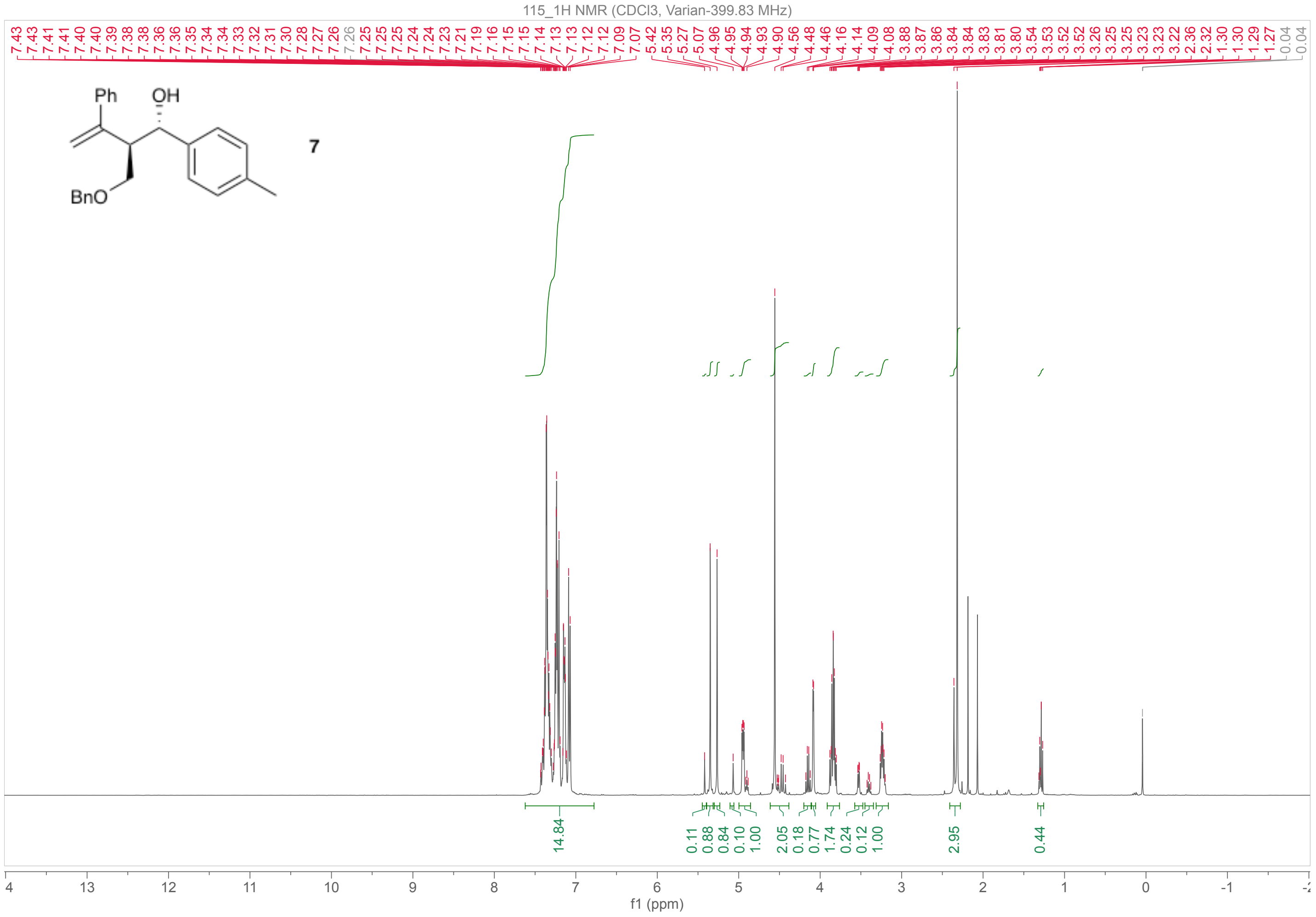
115_1H NMR (CDCl3, Varian-399.83 MHz)
7.43 7.43 7.41 7.41 7.40 7.40 7.39 7.38 7.38 7.36 7.35 7.34 7.34 7.33 7.32 7.31 7.30 7.28 7.27 7.26 7.25 7.25 7.25 7.24 7.24 7.23 7.21 7.19 7.16 7.15 7.15 7.14 7.13 7.13 7.12 7.09 7.07 5.42 5.35 5.27 5.07 4.96 4.95 4.94 4.93 4.90 4.56 4.48 4.46 4.16 4.14 4.09 4.08 3.88 3.87 3.86 3.84 3.84 3.83 3.81 3.80 3.54 3.53 3.52 3.52 3.26 3.25 3.23 3.23 3.22 2.36 2.32 1.30 1.30 1.29 1.27 0.04 0.04
Ph
OH
BnO
7
14.84 0.11 0.88 0.84 0.10 1.00 2.05 0.18 0.77 1.74 0.24 0.12 1.00 2.95 0.44
f1 (ppm)

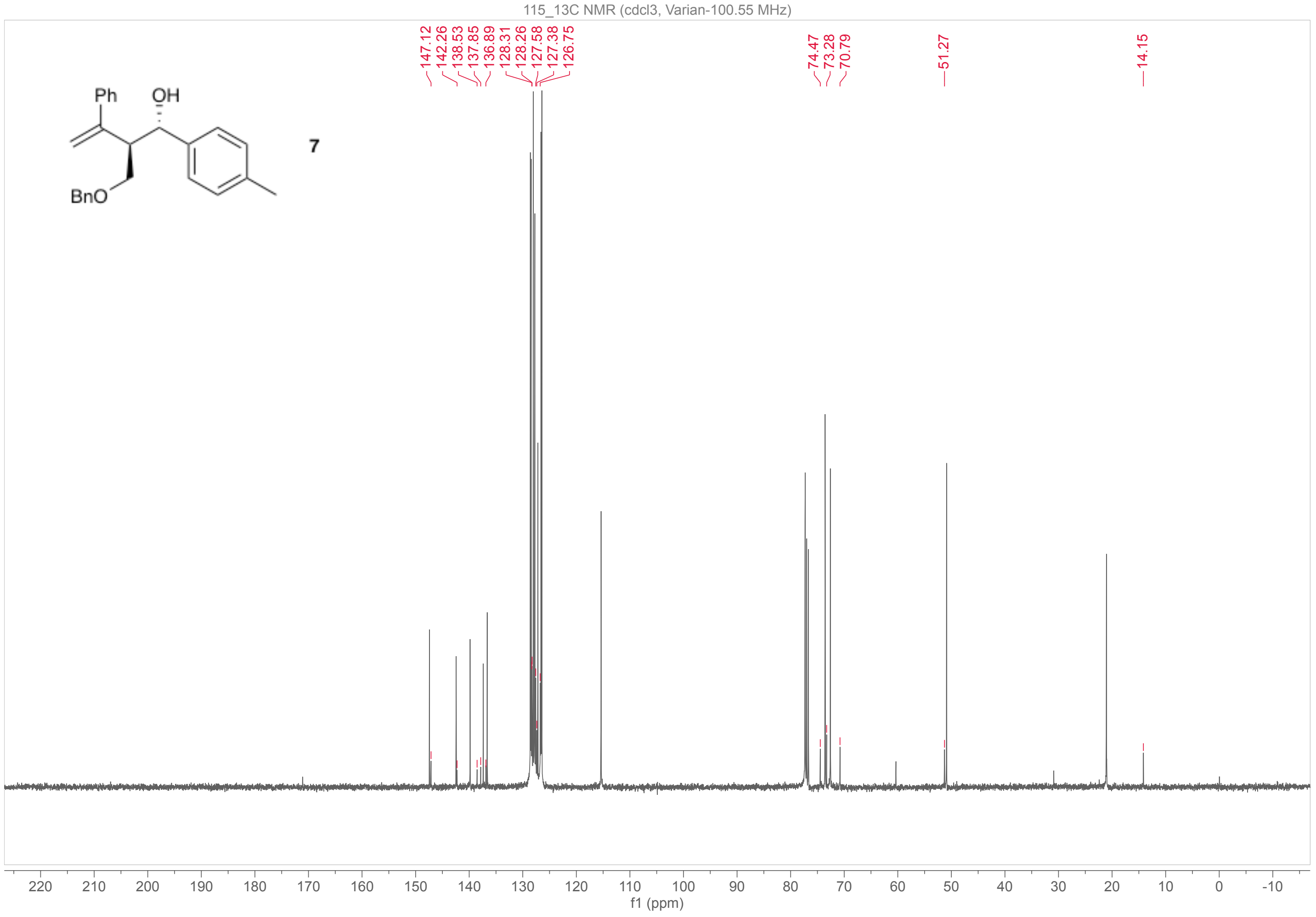
115_13C NMR (cdcl3, Varian-100.55 MHz)
147.12 142.26 138.53 137.85 136.89 128.31 128.26 127.58 127.38 126.75
74.47 73.28 70.79
51.27
14.15
Ph
OH
BnO
7
f1 (ppm)

116_1H NMR (CDCl3, Varian-399.83 MHz)

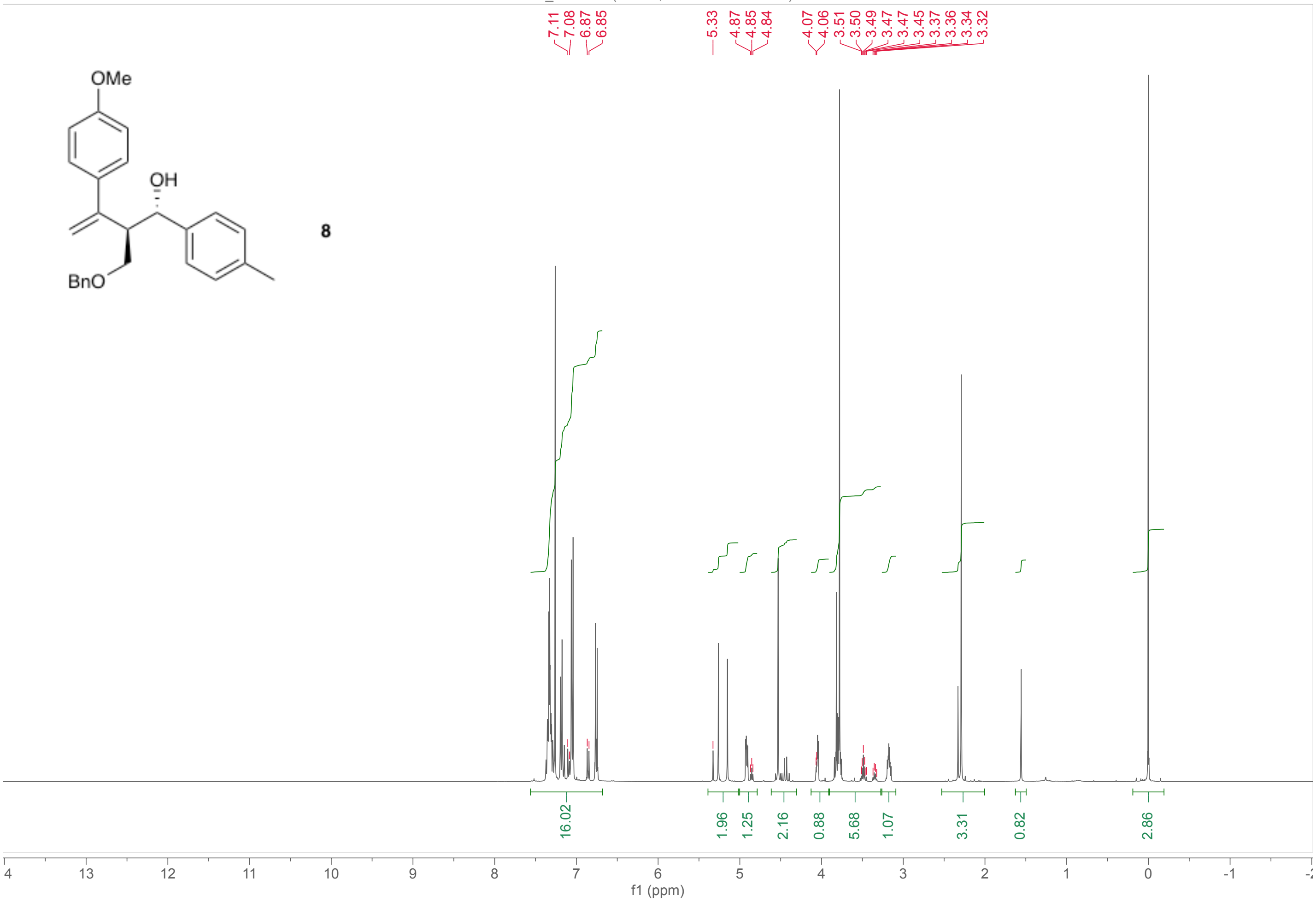

116_13C NMR (CDCl3, Varian-100.55 MHz)

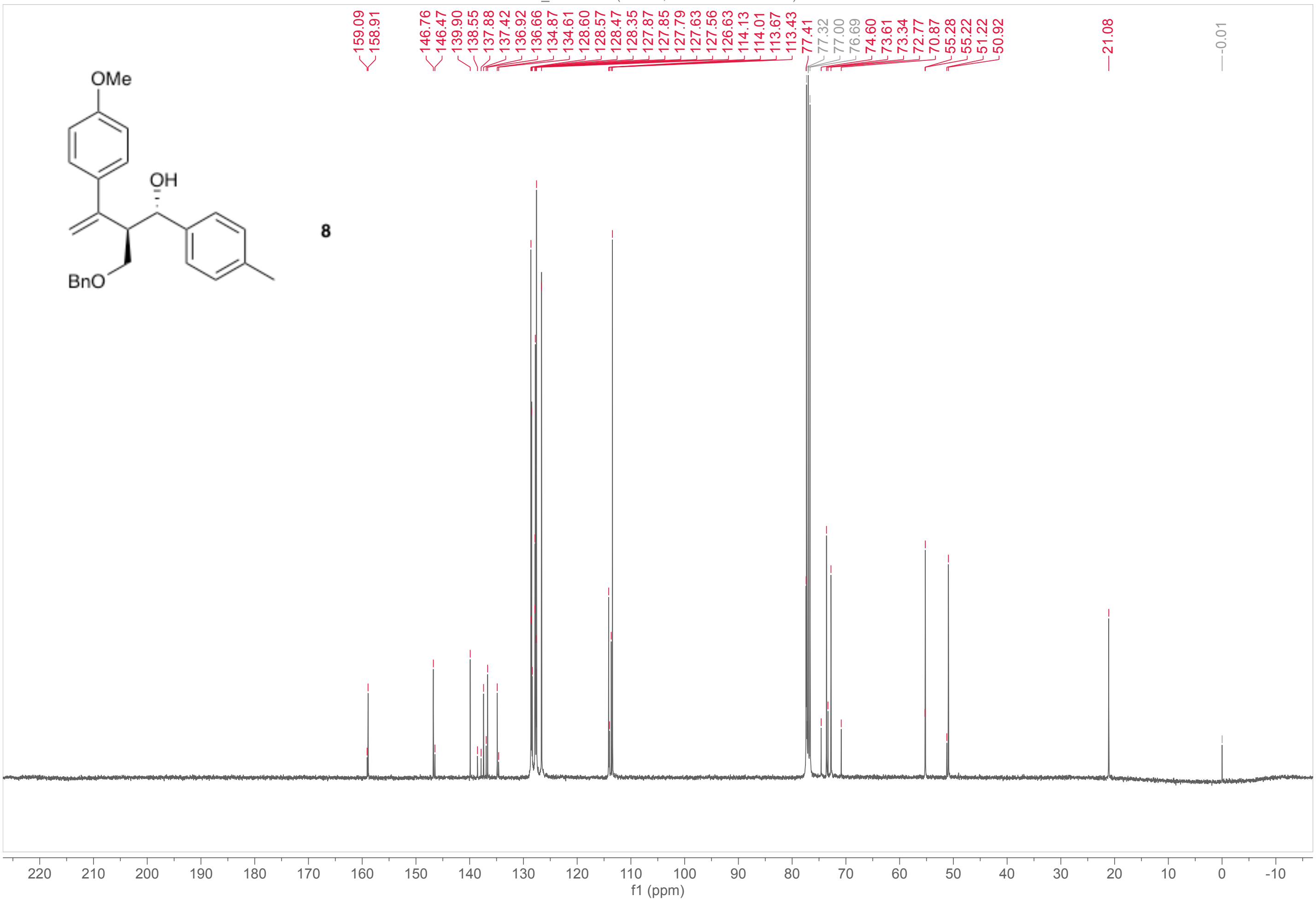

122_1H NMR (CDCl3, 499.94 MHz)

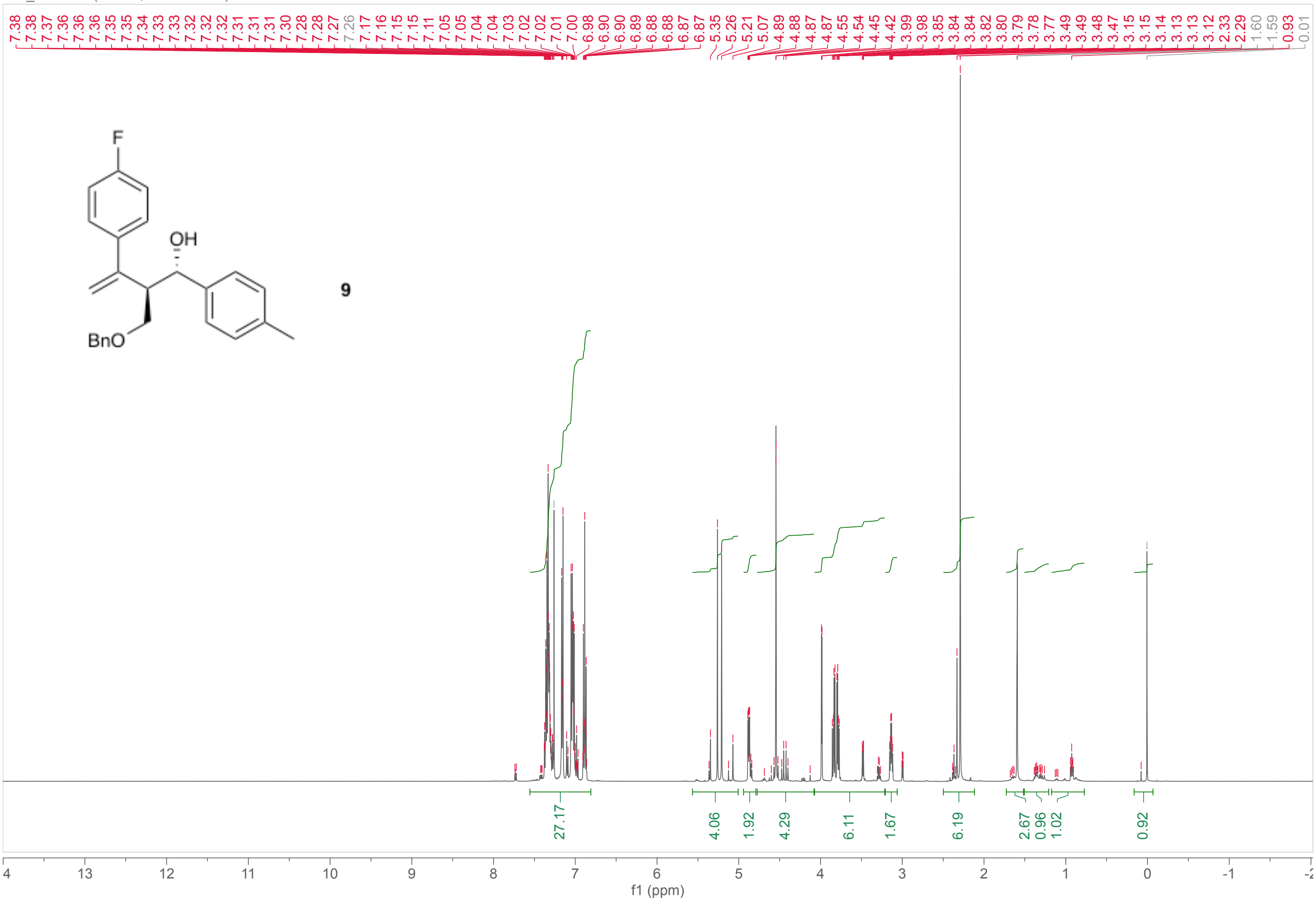

112_13C NMR (CDCl3, Varian-125.72 MHz)

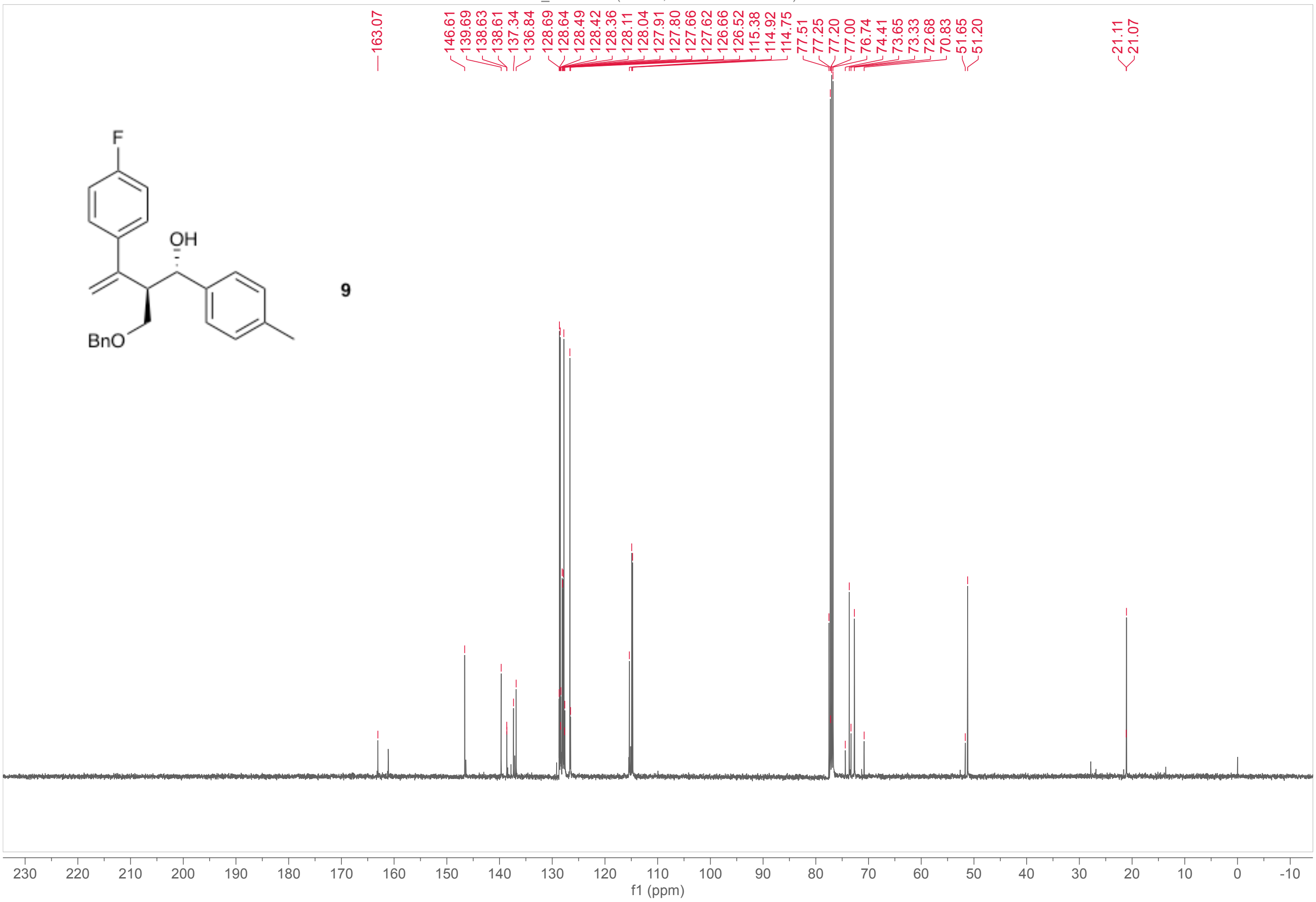

398_1H NMR (cdcl3, Varian-499.94 MHz)

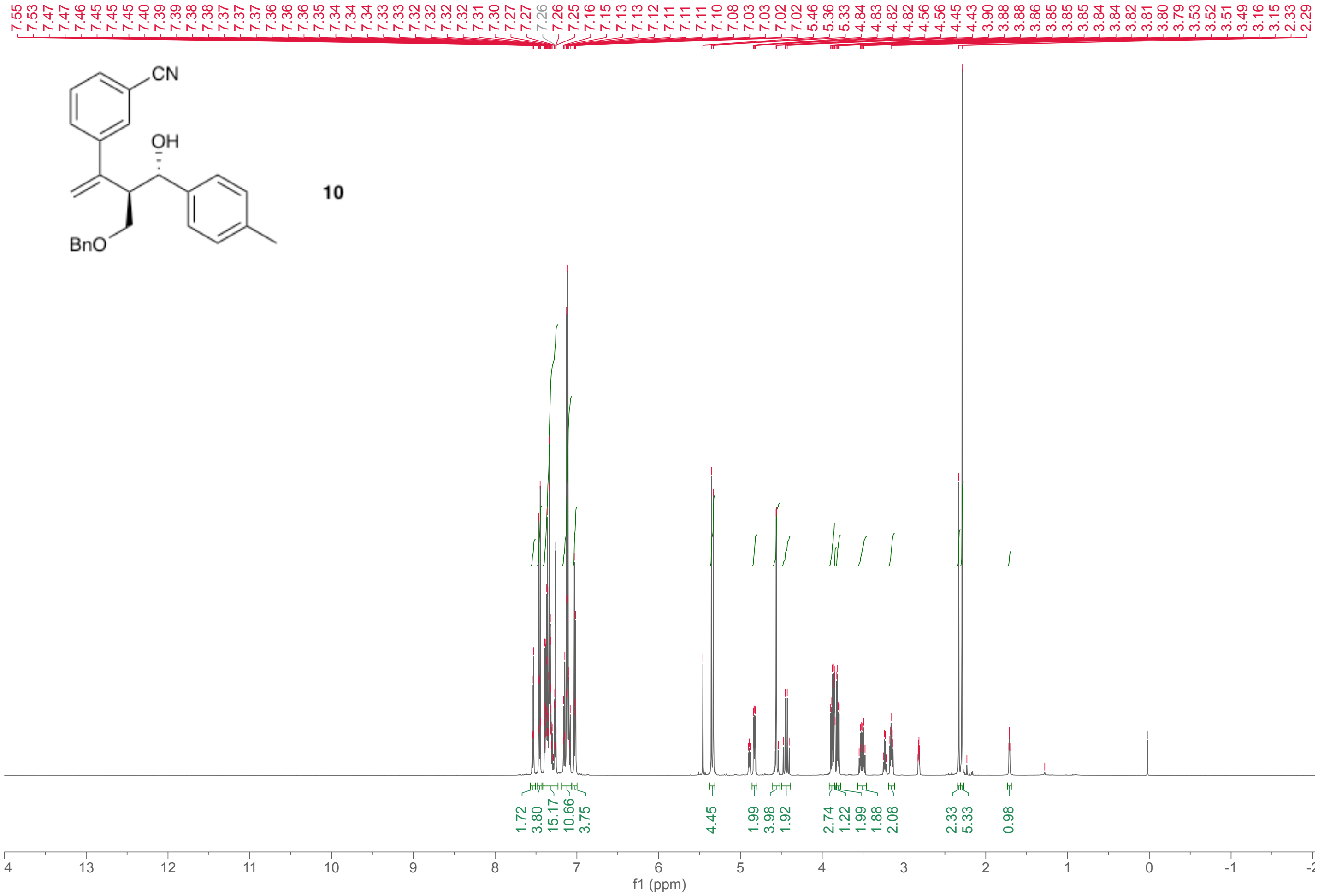

398_13C NMR (cdcl3, Varian-125.72 MHz)

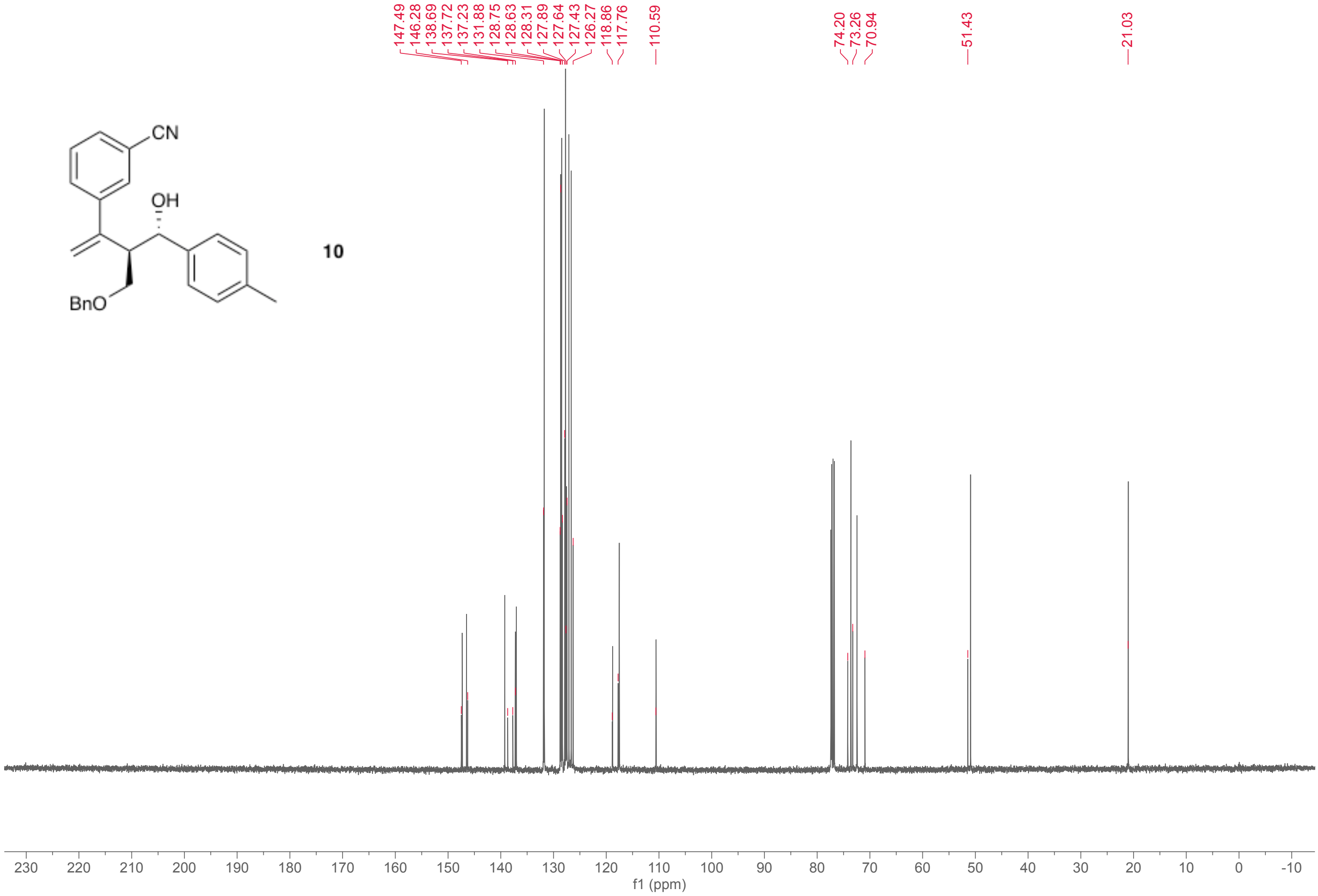

557_1H NMR (cdcl3, 499.94 MHz)

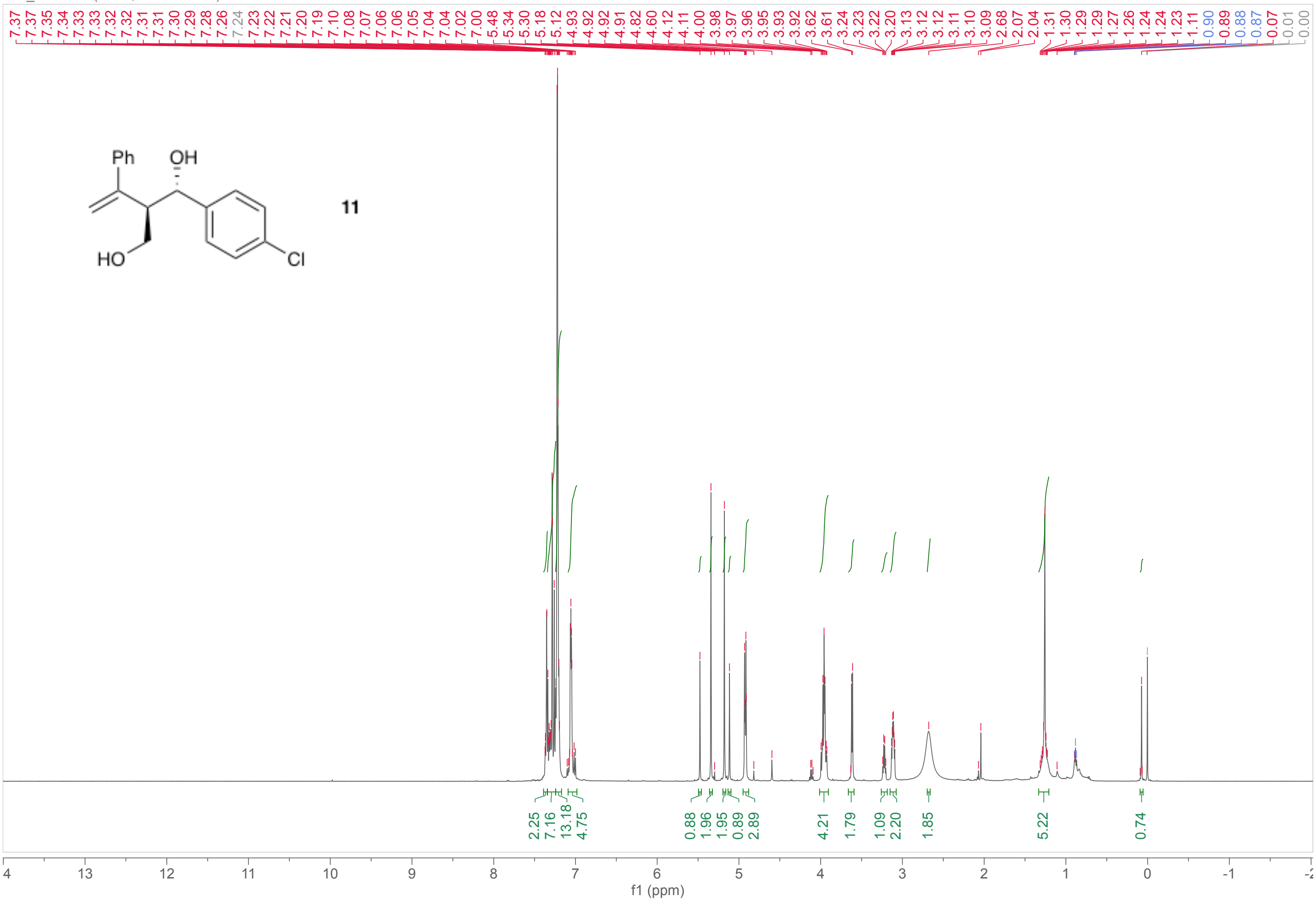

557_13C NMR (cdcl3, Varian-125.72 MHz)

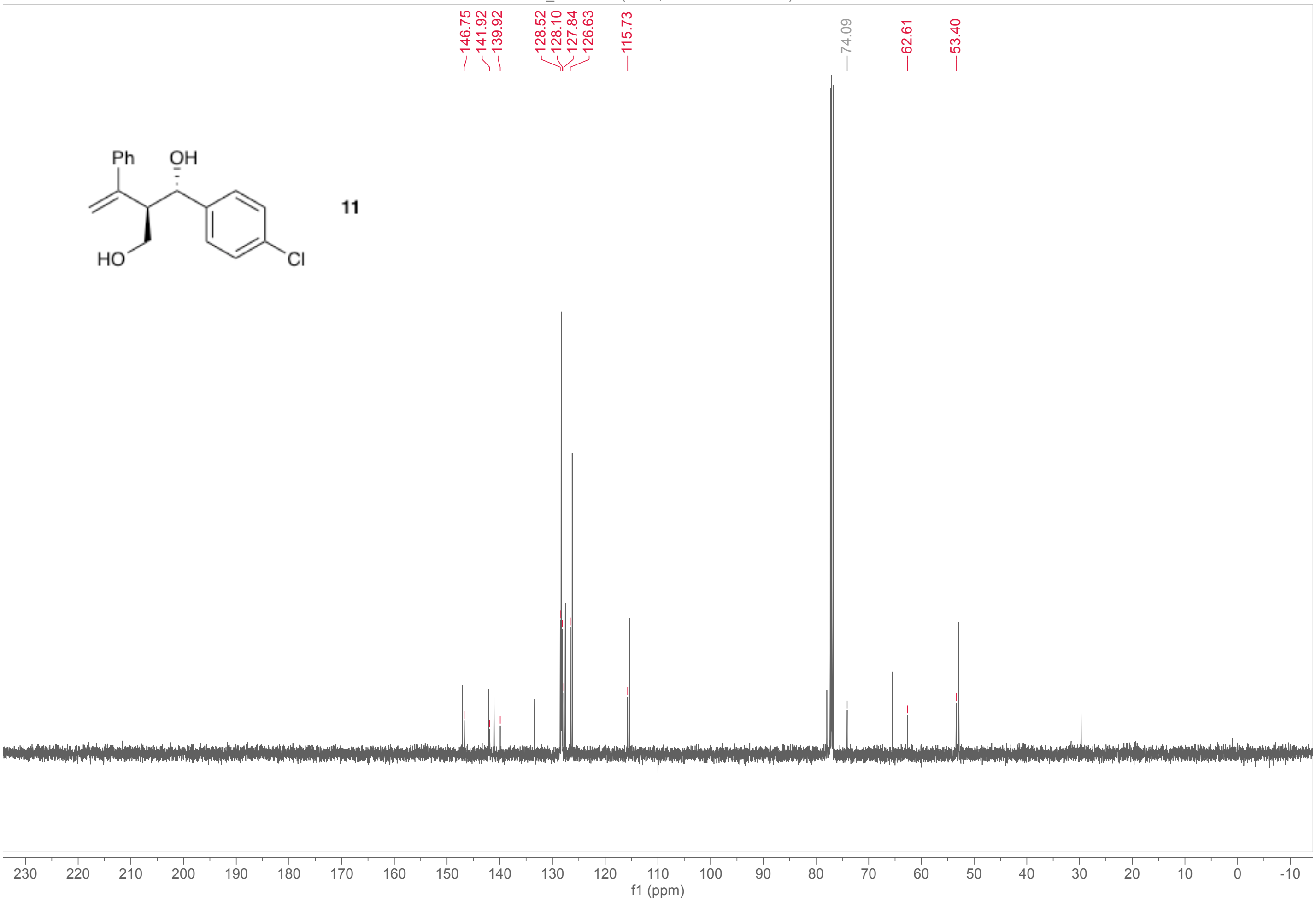

Supplement: File 1 — Experimental part. [file Beilstein_J_Org_Chem-14-1413-s001.pdf]
